# A Small Change in Structure, a Big Change in Flexibility †

**DOI:** 10.3390/molecules28248004

**Published:** 2023-12-08

**Authors:** Nikolay G. Vassilev, Ivo C. Ivanov

**Affiliations:** 1 Institute of Organic Chemistry with Centre of Phytochemistry, Bulgarian Academy of Sciences, Acad. G. Bontchev Str. Bl. 9, 1113 Sofia, Bulgaria; 2 Faculty Chemistry, Aalen University of Applied Sciences, Beethovenstraße 1, 73430 Aalen, Germany; ivo43bg@yahoo.co.uk

**Keywords:** rotational barrier energy, amide bond, enamine bond, dynamic NMR, reference deconvolution, DFT calculations

## Abstract

Studies of the rotational barrier energy of the amide bond using quantum computing and nuclear magnetic resonance (NMR) are focused mainly on its use as a model of the peptide bond. The results of these studies are valuable not only in terms of the fundamental conformational properties of amide bonds, but also in the design of molecular machines, which have recently attracted interest. We investigate the fluxionality of the amide and enamide bonds of compound 3-[(*E*)-(dimethylamino)methylidene]-1,1-dimethylurea using advanced dynamic NMR experiments and a theoretical evaluation of the density functional theory (DFT) calculation. The dynamic NMR study shows restricted rotation around the amide group (16.4 kcal/mol) and a very high barrier around the enamine group (18.6 kcal/mol). In a structurally similar compound, (*E*)-3-(dimethylamino)-*N*,*N*-dimethylacrylamide (N atom is replaced by CH), the amide barrier is 12.4 kcal/mol and the enamine barrier is 11.7 kcal/mol. The DFT studies of both compounds reveal the electronic origin of this phenomenon. Theoretical calculations reveal the origin of the higher enamine barrier. The better delocalization of the lone pair of electrons on the end nitrogen atom into the antibonding orbital of the neighboring C–N double bond leads to the better stabilization of the ground state, and this leads to a greater increase in the enamine barrier.

## 1. Introduction

The amide bond is a basic unit of proteins and is characterized by unique spatial and energetic properties [1,2]. Over several decades, the dynamics of these bonds were studied to understand protein dynamics. Internal rotation about the amide C–N bond in amides and thioamides has been studied experimentally by NMR spectroscopy in both the gas [3,4,5,6,7,8,9] and liquid phases [10]. Experimental results have been used to critically evaluate theoretical methods for the calculation of barrier heights. The origin of the C–N rotational barrier and its relation to the amide resonance has been discussed in recent years [11,12,13,14,15,16].

Recently, the electronic effect of polar substituents on the barrier of internal rotation around the amide bond in *p*-substituted acetanilides [17,18] and thioacetanilides [19] was studied at the B3LYP/6-31G(d,p) level. Several linear relationships were established linking the barrier heights with the structural and electronic parameters that characterize the amide and thioamide groups. The results obtained are consistent with the view of classical amide resonance being the origin of the higher rotational barriers in thioamides than in amides.

Recently, ab initio studies [20] of an internal rotation barrier around a C–N bond in *N*,*N*-dimethylcinnamamides, which were previously investigated by dynamic NMR spectroscopy [21,22], have been published. Scientific interest in cinnamamides and, in particular, coumaric amides has increased in recent years due to their potential antioxidant activity [23,24]. The free energy of activation of substituted cinnamamides is very well reproduced using MP2(fc)/6-31+G*//6-31G* energies and the PCM/6-31G* energy change from gas phase to chloroform. For all compounds studied, the *anti* transition state (*anti* TS) is more stable and determines the rotational barrier. The remote effect of the phenyl substituents in the studied compounds has a purely electronic origin, which was shown by the correlation between the C–N bond order difference and the calculated energy barrier [20].

**Figure 1:**
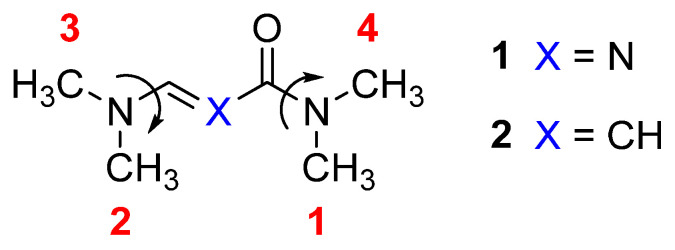
Structures of studied *N*,*N*-dimethylamide derivatives. **1**—3-[(*E*)-(dimethylamino)methylidene]-1,1-dimethylurea, **2**—(*E*)-3-(dimethylamino)-*N*,*N*-dimethylacrylamide.

Conformational studies of amide bonds have been extensively carried out also in view of their potential applications in the field of molecular machines [25,26] (molecular gear systems, single-molecule motors, and single-molecule devices [27]). Here, we present a combined dynamic nuclear magnetic resonance (DNMR) spectroscopy and density functional theory (DFT) study of the C–N bond rotation of the amide and the enamine groups in compound **1** in order to understand the unexpectedly high energy barrier of enamine group. At the same time, to understand the origin of this phenomenon, we present a DFT study of the C–N bond rotation of amide and enamine groups in compound **2**, which is an analogue of **1**, in which the N atom is substituted by a CH group (Figure 1).

## 2. Results

### 2.1. Interpretation of VT-NMR and Characterization of the Studied Compound **1**

Compound **1** was synthesized recently by an alternative synthetic approach [28,29,30]. Its structure has been confirmed by NMR and X-ray analyses. However, this compound shows flexibility, whose rate falls in the NMR time scale. We studied the amide and enamine rotational barriers at both the CC–N and C(O)–N bonds (Figure 1). Before investigating the rotational barriers using ^1^H VT NMR, the four N-Me signals were assigned in order to understand which rotations occur. If we number the methyl groups by increasing chemical shift, then the methyl signals 1 and 4 are exchanging, whereas signals 2 and 3 are exchanging as well, albeit slightly more slowly (Figures S1 and S6). The assignment of the methyl signals in the proton spectrum is supported by the following observations: the NOESY spectrum shows proximity between methyl 3 and CH (Figure S2); the ^1^H,^15^N-HMBC spectrum shows the proton–nitrogen correlations between the methine proton and those of enamine methyl groups (Figure S5). A coupling constant of 0.4 Hz between protons from *N*-methyl 2 and the methine proton is observed. The signals of amide methyls 1 and 4 were determined using the anisotropy effect of the carbonyl group. The assignments of the methyl signals in the carbon spectrum are based on the HSQC correlations (Figure S4).

### 2.2. Dynamic NMR Studies

**Figure 2:**
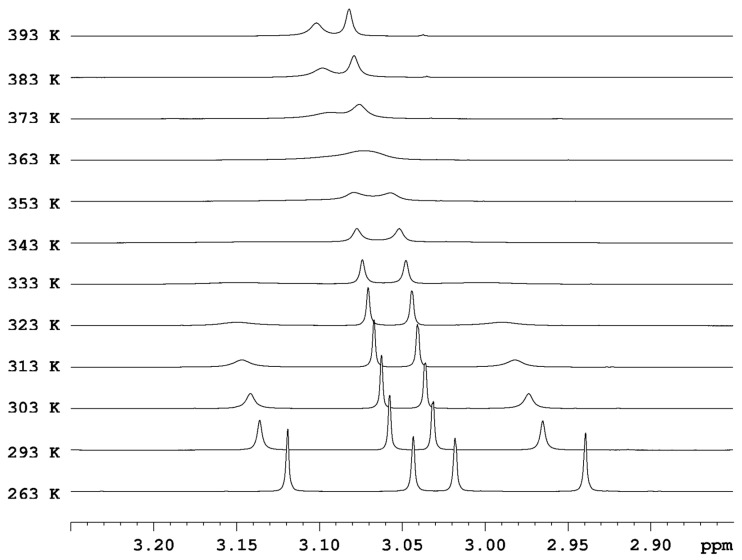
Stacked plot of ^1^H NMR spectra of compound **1** in TCE-d2 at different temperatures.

We measured the VT spectra of compound **1** in two aprotic solvents: in CDCl_3_ in steps of 5 K between 283 and 328 K and in TCE-d2 in steps of 10 K between 263 and 393 K. The *N*-methyl signals of the amide group (denoted as 1 and 4 in Figure 1) are more sensitive to the increase in the temperature and in CDCl_3_ become broadened at 313 K, while the *N*-methyl signals of the enamine group (denoted as 2 and 3 in Figure 1) remain in a slow range of exchange until 323 K (Figure S8). In TCE-d2, the signals of both exchanging methyl pairs shift from a slow range of exchange to a fast range of exchange (Figure 2 and Figure S11). The methyl signals of the amide group merge into a lower-field signal (3.10 ppm) at 393 K, while the methyl signals of the enamine group merge into a single stronger-field signal (3.08 ppm) at 393 K.

The VT spectra of compound **1** in CDCl_3_ are in the slow range of exchange and therefore they are suitable for study using 2D EXSY, 1D EXSY, or magnetization–transfer experiments. We prefer to adopt 2D EXSY experiments as a well-established approach and an easy way to derive rate constants from 2D integrals.

**Figure 3:**
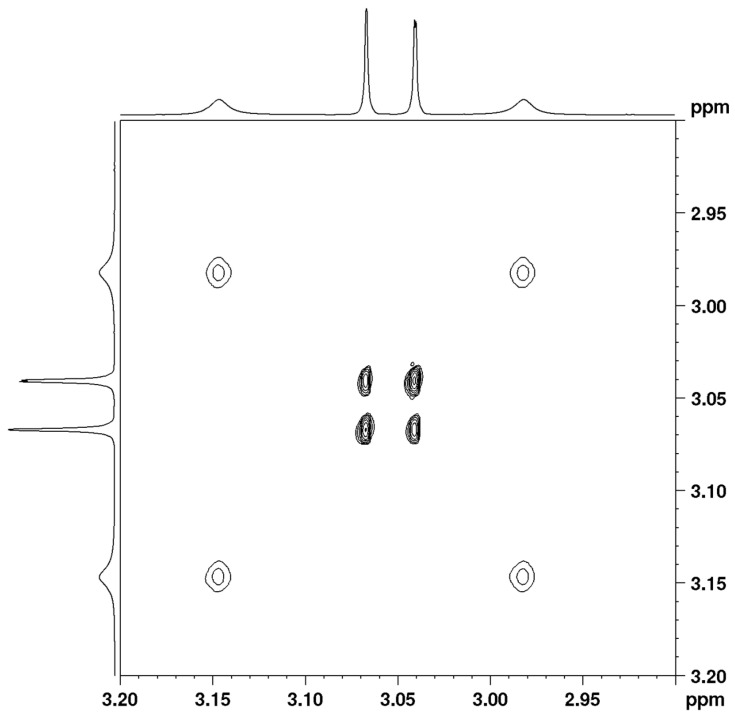
EXSY spectrum of complex **1** in TCE-d2 at 313 K using mixing time of 1.0 s.

The VT spectra of compound **1** in TCE-d2 were also studied using 2D EXSY spectra in a slow range of exchange (Figure 3 and Figure S12) and using complete line shape analysis (CLSA) in the entire temperature range (Figure S30).

The 2D EXSY results in both solvents are almost equal and show no solvent dependence of the rotational barriers (Table 1 and Table 2). The unexpected result is that the enamine rotational barrier is 18.6 kcal/mol, a much higher value than the amide rotational barrier of 16.4 kcal/mol. The analogue of compound **1** in which the X is CH (compound **2**) was studied in the mid-1970s by ^13^C NMR spectra, and the rotational barriers of the amide and enamine groups were estimated using the coalescence method [31]. The experiment was performed in acetone-d6 and the enamine rotational barrier (11.7 kcal/mol) was lower than the amide rotational barrier (12.4 kcal/mol). The small structural change in the molecule causes a big change in the flexibility. This fact led us to study compound **1** in depth and find an answer to the question of why the two compounds (**1** and **2**) have such different rotational flexibility.

The experiment in TCE-d2 was designed with the idea of studying compound **1** in the most rigorous way possible. The sample was prepared by adding one drop of anisole [32,33] (a compound with a singlet signal at 3.81 ppm), which is a suitable compound for the application of the reference deconvolution procedure [34,35]. The relaxation times T_1_ and T_2_ of the reference signal at different temperatures were measured (Table S5) and their temperature dependence was determined (Figures S14 and S15). From this dependence, the values of T_2_ were calculated, which were used in the reference deconvolution procedure in order to remove both line distortion and the signals that we were interested in to be of natural half-width. The newly developed signal-to-noise ratio enhancement program [36] was used to process the FIDs, instead of the previously used LP [32] and MEM [37]. Slow exchange chemical shifts were extrapolated to high temperatures. Thus, all that remained was to fit the rate constants in the procedure of CLSA. The CLSA results are very close to the 2D EXSY results and are complementary (Table 1 and Table 2). In general, 2D EXSY is applicable for slow exchange and the line shape analysis is most sensitive at intermediate exchange.

**Figure 4:**
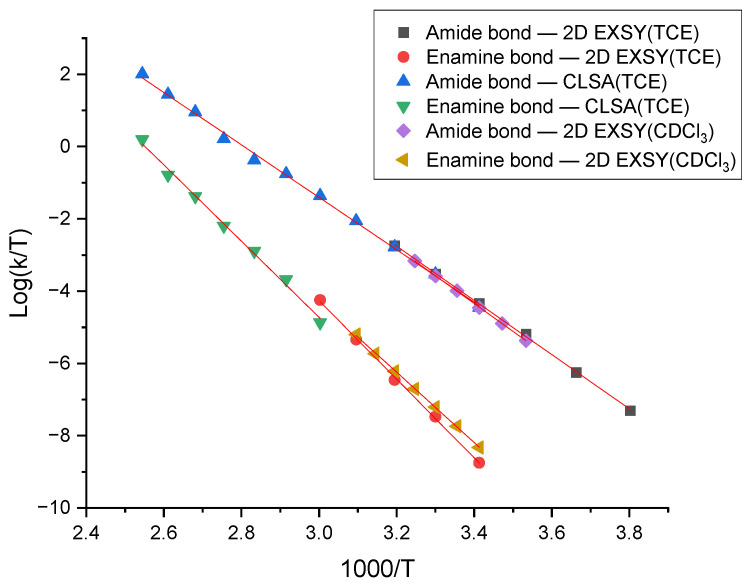
Eyring plot of rate constants of restricted rotation around amide and enamine bonds using 2D EXSY and CLSA rate constants. The origin of the data is given in the inset.

Figure 4 presents the Eyring plot of the rate constants obtained by different methods. These plots demonstrate very good linear dependence for all sets of kinetic data. The correlation coefficients of all plots are very good and are presented in Tables S2, S4, S7, and S8. 

### 2.3. DFT Calculations

**Figure 5:**
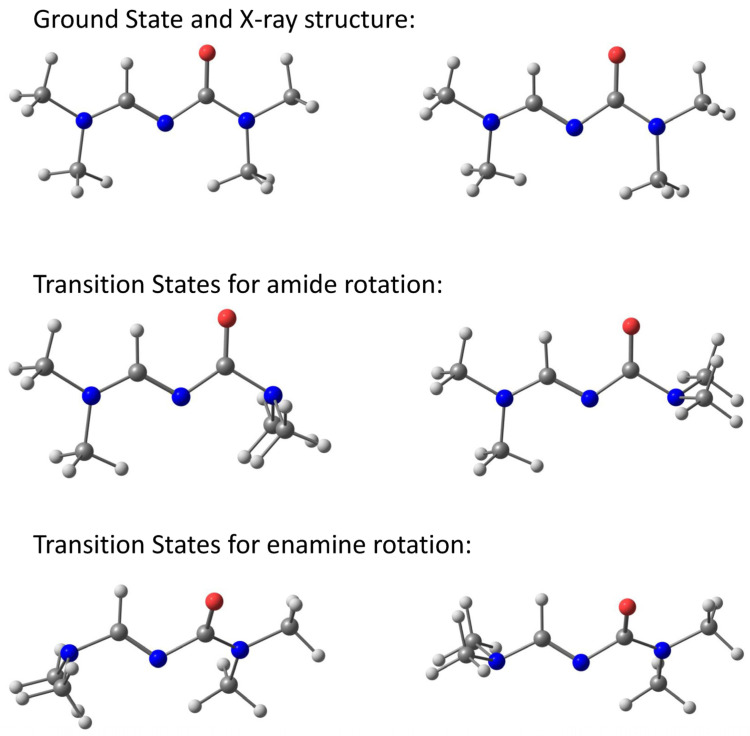
SMD (CDCl_3_)/B3LYP/6-311++G (d,p) optimized GS and TS structures of compound **1**. Top row: the ground state and X-ray structures; middle: the *syn* and *anti* transition state structures for the rotation of the *N*,*N*-dimethylamido group; bottom: the *syn* and *anti* transition state structures for the rotation of the *N*,*N*-dimethylenamino group. Red balls—oxygen, blue balls—nitrogen, big grey balls—carbon and small grey balls—hydrogen.

A natural continuation of the experiments in the analysis of the observed phenomenon is the DFT study of the hindered rotation around the amide and enamine bond (Figure 2). In addition to the ground state (GS) geometry for both compounds, transition structures (TS) were localized as well. For each rotation, two TSs are possible (Figure 5). Popular functionals B3LYP [38] and M06-2X [39,40] with the 6-311++G (d,p) basis set [41] were used in the DFT calculations. The SMD [42] model was used to account for the solvent effect. Table 1, Table 2, Table 3 and Table 4 show that the theoretically calculated rotational barriers of both compounds and both rotations are in very good agreement with the experimental data.

### 2.4. Origin of Rotational Barriers

**Figure 6:**
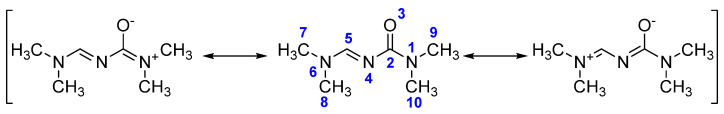
The resonance structures of compound **1**.

The studied compound **1** is a push–pull molecular system, for which several resonance structures can be written (Figure 6). Even from these structures, it can be concluded that there is a large delocalization over the heavy atom framework and the C–N enamine single bond has a partial double bond character.

The computed geometrical parameters of **1** are in excellent agreement with the X-ray crystal structure [30] (Table 5). The heavy atom frameworks of **1** and **2** are planar in GS. When comparing the structural parameters of **1** and **2**, only the bond lengths of X4 with neighboring C2 and C5 differ significantly, which is to be expected. 

The C2–N1 amide bond was studied intensively in the past, and the heavy atom framework of the studied amides in the GS was found to be essentially planar and close to C_S_ symmetry. The transition state geometries of the studied amides can be optimized in C_S_ symmetry and yield two transition states: *syn* and *anti*. The most significant structural changes in the process of the rotation of amides towards the transition states are that the nitrogen is pyramidalized and the C–N bond lengthens by 0.07–0.08 Å. However, the C=O bond length shortens by 0.01–0.02 Å only. This indicates that the carbonyl group is relatively unaffected by this rotation. The origin of the C–N amide rotational barrier and its relation to amide resonance has also received much attention in the last few years [11,12,13,14,15,16] and therefore we will discuss here mainly the enamine rotational barrier, which has only been discussed once in the literature [43]. The X4–C5 bond (Figure 3) of both molecules does not change appreciably from GS to TSs. However, the change in bond length in the GS and TS geometries is significant for the C5–N6 bond. In **1**, it increases in *syn*-TS with 0.091 Å and in *anti*-TS with 0.083 Å. In **2**, it increases in *syn*-TS with 0.087 Å and in *anti*-TS with 0.078 Å. In the GS, the C5–N6 bond adopts a partial double bond character (as indicated by the Wiberg Bond Index values of 1.298 and 1.237 for **1** and **2**, respectively), but in the rotated TS, it becomes a single bond (Table 6). Moreover, the enamine nitrogen atom adopts a planar geometry in the GS, but it is pyramidalized in the TS. This is further supported by the calculated and observed C–N–H and H–N–H bond angles around the enamine nitrogen atom, which correspond to the sp^2^ hybridized nitrogen atom in the GS (Table 6). However, in the TS, the hybridization changes to sp^3^, as indicated by the respective bond angles (Figure 5). The above data indicate that the actual ground state geometry can be envisaged as a resonance hybrid of three structures (Figure 6). The left and middle structures represent the resonance of the amide bond, while the middle and right structures represent the resonance of the enamine bond. The left structure contains a double bond between C2 and N1 and this partial double bond is responsible for the observed barrier of the amide group. The right structure contains a double bond between C5 and N6 and this partial double bond is responsible for the observed barrier of the enamine group.

The insight obtained about the nature of the C5–N6 bond (Figure 3) from the NBO analysis agrees well with those acquired from the geometrical parameters. Our discussion of the NBO analysis of **1** and **2** repeats most of the conclusions made earlier on the origin of the enamine rotational barrier [43]. In GS, this bond has a partial double bond character, while, in TSs, it is a single bond and the nitrogen atom changes from sp^2^ in GS to a sp^3^ hybridized atom in TSs. The lone pair of electrons on N6 has reduced occupancy in the GS than in the TSs (Table 6) because, in the GS, a part of the electron density of the lone pair is delocalized into the antibonding orbital of the adjacent C–N double bond. Such delocalization is not possible in the TS as the participating orbitals are orthogonal to each other. The orbital housing the lone pair in the GS is an in-plane p orbital, but it attains some s character in the TS, thereby making the nitrogen atom more electronegative in the TS. 

Natural charges computed at the same level of theory predict a higher negative charge for the N6 atom in the TSs (Table 7), which correlates well with the increased occupancy of N6 in the TS (Table 6). However, the charge on the C5 atom does not change appreciably in both the GS and TSs. On the other hand, the N4 atom, which is double bonded to C5, carries a higher negative charge in the GS.

The stabilization energies resulting from the delocalization of the N6 lone pair of electrons into the antibonding orbital of the neighboring C5–N4 double bond decrease from **1** to **2** (Table 8). Along with them, the rotational barrier also decreases from **1** to **2**. This stabilizing interaction is responsible for the partial double bond character of the C5–N6 single bond in the GS (right structure of Figure 6) and therefore the barrier height. 

The calculated main steric exchange energies (Table 9) for the GS and TS structures of **1** and **2** did not show significant differences that could explain the significant difference in the enamine barrier of **1** and **2**.

All these data are consistent with our resonance-based explanation of the origin of the rotational barrier about the C5–N6 bond. This barrier has a purely electronic origin, which recently was demonstrated by the relationship between the C–N bond order difference and calculated energy barrier [20]. The proportion of experimentally determined enamine to amide barriers in **1** (18.6/16.4 = 1.13) is in very good agreement with the proportion of Wiberg bond orders (Table 10) of enamine to amide C–N bonds in GS of **1** (1.298/1.169 = 1.11).

## 3. Materials and Methods

### 3.1. Sample Preparation

The studied compound **1** was synthesized according to the previously described synthetic procedure [30].

### 3.2. Experimental Methods

^1^H and ^13^C spectra were recorded on a Bruker II+ 600 spectrometer (BBO probe, Bruker BioSpin GmbH, Ettlingen, Germany) at 600.13 for ^1^H NMR and 150.92 MHz for ^13^C NMR, with TMS as an internal standard for chemical shifts (δ, ppm). Two NMR samples (0.1 M solution of **1** in CDCl_3_ and 0.1 M solution of **1** in 1,1,2,2-tetrachloroethane-d2 (TCE)) were degassed under a vacuum and sealed. One drop of anisole was added to the second sample, the methyl group signal at 3.78 ppm of which was used as a reference for reference deconvolution (RD). The spectra in CDCl_3_ were recorded in steps of 5 K between 283 and 323 K, while the spectra in TCE-d2 were recorded in steps of 10 K between 263 K and 393 K. Temperature calibration was done with a B-VT 3000 unit (it was checked and calibrated with methanol and ethylene glycol reference samples). ^1^H NMR spectra were acquired using a spectral width of 10 kHz, an acquisition time of 3.4 s, and 32 scans, zerofilled to 64 k datapoints (0.15 Hz per point), and processed without apodization.

The 2D EXSY spectra (noesygpphzs) in CDCl_3_ were recorded on a BBO probe in steps of 5 K between 283 and 323 K. The spectra were acquired using a spectral width of 4.2 kHz, 2048 × 256 complex time domain datapoints, mixing times in the range of 0.03 to 1.5 s, and 2 scans in about 45 min. The spectra were zerofilled to 4096 × 4096 datapoints and processed with a shifted square sine bell apodization in both dimensions.

The 2D EXSY spectra in TCE-d2 were recorded on a BBO probe in steps of 10 K between 263 and 333 K. The spectra were acquired using a spectral width of 0.3 kHz, 2048 × 256 complex time domain data points, mixing times in the range of 0.03 to 1.5 s, and 2 scans in about 50 min. Linear prediction (32 coefficients and 256 points) in F1 was applied. The spectra were zerofilled to 4096 × 4096 data points and processed with a shifted square sine bell apodization in both dimensions. The measurement details and processing of the studied samples can be found in the Supporting Information. Exchange rates were calculated from diagonal and crosspeak integrals using EXSYCalc (MestreLab Research S.L.).

The relaxation rates *T*_1_ and *T*_2_ of the methyl group of the reference compound were measured in the temperature range 263–333 K (Table S5) using the *tlir* (inversion–recovery, *T*_1_) and *cpmg* (Carr–Purcell–Meiboom–Gill, *T*_2_) programs of the standard Topspin 3.6pl4 Bruker software. The measured *T*_2_ values were smoothed with an exponential temperature function (Figure S15). The estimated values were used in the reference deconvolution procedure in order to obtain spectra free from inhomogeneity broadening. An automatic correction of noise spikes was applied to the deconvoluted FIDs (Figure S16).

Reference deconvolution [34,35] was performed using a line broadening factor Δ*v*_1/2_ = (π*T*°_2_)^−1^, where *T*°_2_ is the natural spin–spin relaxation time of the reference signal.

The spectra of the methyl groups of **1** at the lowest five temperatures were fitted with the DNMR module of the Topspin program, fixing the rate constants, known from the 2D EXSY experiments, and adjusting the *T*°_2_ values only. The calculated relaxation times *T*°_2_ were then fitted to an exponential function [44] and extrapolated for higher temperatures. In the following CLSA calculations, for the whole temperature range, only the rate constants were adjusted.

Error analysis: Usually, the presented errors in the activation parameters are statistical errors based on the scattering of the data points around the Eyring straight line only. The errors in this analysis are due to inaccuracies in both the calculated rate constants, *k*, and the measured temperatures, *T*, and are computed according to the error propagation equations of Binsch [45] and Heinzer and Oth [46]. The absolute error in temperature is assumed to be not more than ±0.5 K. The relative errors in *k* are estimated to be not more than ±10% at all temperatures according to the precision of the volume integration of peaks. The error analysis was performed using a self-made computer program using the cited equations.

### 3.3. Computational Methods

All calculations were performed by means of quantum chemical calculations at the density functional theory (DFT) level using the Gaussian16 Rev. C.01 program package [47] with tight optimization criteria.

The geometries of all compounds were fully optimized and the corresponding transition states were localized using the B3LYP [38] or M06-2X [39,40] functional with the 6-311++G (d,p) basis set [41]. The solvent effect was included implicitly in the optimizations via the SMD [42] model with the built-in parameters for solvents CHCl_3_ and acetone. As TCE is not, by default, parametrized within the SMD model implemented in Gaussian, the following parameters were used: eps = 8.42 (https://www.stenutz.eu/chem/dielectric_ri.php, accessed on 5 December 2023); epsinf = 2.028346 (n^D^_20_ = 1.495, epsinf = n^2^ = 2.235025); HBondAcidity = 0.16 [48]; HBondBasicity = 0.12 [48]; SurfaceTensionAtInterface = 52.031418 (https://www.stenutz.eu/chem/dielectric_ri.php, accessed on 5 December 2023); ElectronegativeHalogenicity = 0.667; CarbonAromaticity = 0.00. The nature of all critical points was confirmed by means of vibrational analysis.

The ∆*H*, ∆*S*, and ∆*G* values were calculated at T = 298.15 K at the same level of theory including zero-point energy in the particular solvent environment (represented by relative permittivity) and vibrational, rotational, and translational thermal energy corrections.

The NBO calculations were performed as they were implemented in the Gaussian16 Rev. C.01 program package [47]. We applied the 7.0 version of the NBO program to study the steric effects (keyword steric) [49]. The pyramidalization angles were determined according to the scheme proposed by Haddon [50].

## 4. Conclusions

The C–N rotational energy barriers for the *N*,*N*-dimethyl groups in two ends of compound **1** have been determined experimentally by dynamic NMR spectroscopy. As expected, the restricted rotation energy around the amide group is 16.4 kcal/mol, but an unexpectedly very high rotational barrier around the enamine group (18.6 kcal/mol) was measured.

From the classical viewpoint, the enamine barrier increases with the increasing bond order and there are good correlations between the bond order and barrier height. Theoretical calculations show that the origin of the barrier lies in the delocalization of the lone pair of electrons on the end nitrogen atom into the antibonding orbital of the adjacent C–N double bond in the ground state. In compound **1**, the stabilization energy E^(2)^ of this delocalization is much higher than the same energy in compound **2**, and this explains the much higher enamine rotational barrier in **1**. 

## Figures and Tables

**Table 1:** Activation parameters of compound **1** for the amide-restricted rotation calculated using different theoretical methods and compared with experiment.

Method	ΔH^≠^(298 K)	ΔS^≠^(298 K)	ΔG^≠^(298 K)	ΔG^≠^(298 K)^eff^
1 ^1^				17.2
2 ^2^				16.5
3 ^3^				16.6
4 ^4^				16.8
2D EXSY in CDCl_3_	15.3 ± 1.1	−3.9 ± 3.7	16.4 ± 0.1	
2D EXSY in TCE-d2	14.9 ± 0.5	−5.0 ± 1.9	16.4 ± 0.1	
CLSA in TCE-d2	14.4 ± 0.4	−6.7 ± 1.4	16.4 ± 0.1	
All data in TCE-d2	14.4 ± 0.3	−6.7 ± 1.0	16.4 ± 0.1	

^1^ SMD (CDCl_3_)/B3LYP/6-311++G (d,p). ^2^ SMD (CDCl_3_)/M062X/6-311++G (d,p). ^3^ SMD (TCE)/B3LYP/6-311++G (d,p). ^4^ SMD (TCE)/M062X/6-311++G (d,p). ΔG^≠^(298 K)^eff^ is calculated by summing up the rates through the two possible TS.

**Table 2:** Activation parameters of compound **1** for the enamine-restricted rotation calculated using different theoretical methods and compared with experiment.

Method	ΔH^≠^(298 K)	ΔS^≠^(298 K)	ΔG^≠^(298 K)	ΔG^≠^(298 K)^eff^
1 ^1^				20.7
2 ^2^				20.3
3 ^3^				21.0
4 ^4^				21.3
2D EXSY in CDCl_3_	19.3 ± 1.0	2.2 ± 2.4	18.6 ± 0.1	
2D EXSY in TCE-d2	21.6 ± 0.8	9.1 ± 2.5	18.9 ± 0.1	
CLSA in TCE-d2	20.9 ± 0.8	6.2 ± 2.0	19.1 ± 0.1	
All data in TCE-d2	19.8 ± 0.5	3.2 ± 1.2	18.8 ± 0.1	

^1^ SMD (CDCl_3_)/B3LYP/6-311++G (d,p). ^2^ SMD (CDCl_3_)/M062X/6-311++G (d,p). ^3^ SMD (TCE)/B3LYP/6-311++G (d,p). ^4^ SMD (TCE)/M062X/6-311++G (d,p). ΔG^≠^(298 K)^eff^ is calculated by summing up the rates through the two possible TS.

**Table 3:** Activation parameters of compound **2** for the amide-restricted rotation calculated using different theoretical methods and compared with experiment.

Method	ΔH^≠^(298 K)	ΔS^≠^(298 K)	ΔG^≠^(298 K)	ΔG^≠^(298 K)^eff^
A ^1^				12.5
B ^2^				12.8
C ^3^				12.5
D ^4^				14.1
Experiment in Acetone-d6 [31]			12.4 (Tc = 253 K)	

^1^ SMD (CDCl_3_)/B3LYP/6-311++G (d,p). ^2^ SMD (CDCl_3_)/M062X/6-311++G (d,p). ^3^ SMD (TCE)/B3LYP/6-311++G (d,p). ^4^ SMD (TCE)/M062X/6-311++G (d,p). ΔG^≠^(298 K)^eff^ is calculated by summing up the rates through the two possible TS.

**Table 4:** Activation parameters of compound **2** for the enamine-restricted rotation calculated using different theoretical methods and compared with experiment.

Method	ΔH^≠^(298 K)	ΔS^≠^(298 K)	ΔG^≠^(298 K)	ΔG^≠^(298 K)^eff^
A ^1^				12.4
B ^2^				12.1
C ^3^				11.8
D ^4^				13.8
Experiment in Acetone-d6 [31]			11.7 (Tc = 253 K)	

^1^ SMD (CDCl_3_)/B3LYP/6-311++G (d,p). ^2^ SMD (CDCl_3_)/M062X/6-311++G (d,p). ^3^ SMD (TCE)/B3LYP/6-311++G (d,p). ^4^ SMD (TCE)/M062X/6-311++G (d,p). ΔG^≠^(298 K)^eff^ is calculated by summing up the rates through the two possible TS.

**Table 5:** Comparison of experimental and calculated ^1^ structural parameters of compound **1** as well as calculated ^1^ structural parameters of compound **2**.

Bonds	1 (exptl.)	1 (calcd.)	2 (calcd.)
N1–C2	1.363	1.370	1.369
C2–O3	1.240	1.241	1.237
C2–X4	1.397	1.401	1.468
X4–C5	1.309	1.301	1.360
C5–N6	1.327	1.338	1.346
N1–C2–O3	120.2	121.3	120.8
O3–C2–N4	125.9	124.8	122.2
C2–N4–C5	113.6	115.0	118.0
N4–C5–N6	122.2	123.5	126.8

^1^ SMD (CDCl_3_)/B3LYP/6-311++G (d,p) geometry.

**Table 6:** Hybridization of the lone pair of electrons at enamine N, pyramidalization angle, and rotational barrier calculated at SMD (CDCl_3_)/B3LYP/6-311++G (d,p) level of theory.

Molecule	Occupancy	s (%)	p (%)	Pyramidalization Angle, *θ* (in Degrees)	Rotational Barrier (kcal/mol)
**1**-GS	1.616	0.30	99.68	2.0	20.7
**1**-*syn*-TS	1.895	13.63	86.33	16.9	
**1**-*anti*-TS	1.892	16.03	83.92	18.5	
**2**-GS	1.658	1.38	98.61	4.5	12.4
**2**-*syn*-TS	1.889	12.37	87.60	15.8	
**2**-*anti*-TS	1.894	15.43	84.53	18.4	

**Table 7:** Natural charges of the heavy atom framework calculated at the SMD (CDCl_3_)/B3LYP/6-311++G (d,p) level of theory (for numbering, see Figure 3).

Molecule	N1	C2	O3	X4	C5	N6
**1**-GS	−0.495	0.799	−0.743	−0.688	0.365	−0.436
**1**-*syn*-TS	−0.482	0.796	−0.694	−0.546	0.401	−0.606
**1**-*anti*-TS	−0.482	0.797	−0.695	−0.531	0.391	−0.592
**2**-GS	−0.503	0.652	−0.742	−0.473	0.122	−0.452
**2**-*syn*-TS	−0.479	0.653	−0.697	−0.327	0.107	−0.590
**2**-*anti*-TS	−0.479	0.653	−0.702	−0.317	0.098	−0.588

**Table 8:** NBO second-order stabilization energies E^(2)^ (kcal mol^−1^) corresponding to the main interactions in compounds **1** and **2** calculated at SMD (CDCl_3_)/B3LYP/6-311++G (d,p) level of theory.

Interactions	1-GS	1-*syn*-TS	1-*anti*-TS	2-GS	2-*syn*-TS	2-*anti*-TS
lpN6 -> π* (X4–C5)	70.30			53.31		
lpN6 -> σ* (X4–C5)		8.63	4.60		7.33	2.30
lpO3 -> π* (C2–N1)	21.53	21.59	21.66	25.49	22.48	22.51
lpO3 -> π* (C2–X4)	21.65	24.60	24.40	17.30	17.43	17.19
lpN1 -> π* (C2–O3)	28.70	37.11	36.16	45.53	58.86	65.84
Π (X4–C5) -> π* (C2–O3)	14.18	12.84	12.65	21.12	15.97	16.79

**Table 9:** Selected pairwise steric exchange energies dE (i,j) (kcal/mol) from NBO analysis for disjoint (no common atoms) interactions between NLMOs i,j.

Steric Interactions	1-GS	1-*syn*-TS	1-*anti*-TS	2-GS	2-*syn*-TS	2-*anti*-TS
lpN6 ↔ π*(X4–C5)	15.91			16.95		
lpN6 ↔ σ*(X4–C5)		7.02	3.91		7.69	5.17
lpN6 ↔ σ*(C5–H)		7.86	7.38		9.06	7.94
lpN6 ↔ σ*(C8–H)	3.59	1.87	2.29	1.14	1.93	2.40
lpN6 ↔ σ*(C8–H)	1.75	10.38	2.29	4.63	10.68	2.37
lpN6 ↔ σ*(C8–H)	10.48	2.94	9.48	10.34	3.12	9.35
lpN6 ↔ σ*(C7–H)	7.59	1.84	2.37	6.35	1.91	2.43
lpN6 ↔ σ*(C7–H)	8.74	3.01	9.56	9.97	3.18	9.37
lpN6 ↔ σ*(C7–H)		10.42	2.24		10.72	2.38
lpN6 ↔ σ*(X4–H)					0.62	1.25

**Table 10:** Calculated Wiberg bond orders of compound **1** (**2**) in the NAO basis and SMD (CDCl_3_)/B3LYP/6-311++G (d,p) geometry.

Molecule	Amide C–N	Enamine C–N	C=O	C=X
**1**-GS	1.169	1.298	1.524	1.519
**1**-*syn*-TS	1.207	0.982	1.578	1.842
**1**-*anti*-TS	1.209	0.986	1.576	1.850
**2**-GS	1.158	1.237	1.514	1.598
**2**-*syn*-TS	1.204	0.986	1.563	1.873
**2**-*anti*-TS	1.205	0.991	1.558	1.872

## Data Availability

The data are available within the article and its Supplementary Materials.

## Supplementary Materials

The following supporting information can be downloaded at: https://www.mdpi.com/article/10.3390/molecules28248004/s1, NMR spectra of compound **1**, details of dynamic NMR studies of **1** in CDCl_3_, and TCE-d2 and DFT geometries of **1** and **2**.
