# Supplementary material for: A Small Change in Structure, a Big Change in Flexibility
Source: Molecules. 2023 Dec 8;28(24):8004. doi: 10.3390/molecules28248004 (PMC10745939; doi:10.3390/molecules28248004)
Supplement: Supplementary file 1 [file molecules-28-08004-s001.zip › molecules-2723638-supplementary.pdf]

Supporting Information for "A small change in structure, a big change in flexibility"

Table of Contents

I. ^{1}H, ^{13}C and ^{15}N NMR spectra of compound 1 **2**

II. Dynamic NMR studies **9**

Compound **1** in $CDCl_3$ **9**

Compound **1** in TCE-d2 **13**

Determination of T_1 and T_2 values of the signal of the reference methyl group of anisole in sample of **1** in TCE-d2 **17**

Reference deconvolution of compound **1** in TCE-d2 **18**

CLSA of compound **1** in TCE-d2 **21**

III. DFT calculations **37**

Cartesian coordinates of the optimized GS geometries **38**

Cartesian coordinates of the optimized TS geometries **42**

Activation parameters for amide and enamine rotations of studied compounds **61**

I. ^{1}H, ^{13}C and ^{15}N NMR spectra of compound 1

Compound **1**:

^{1}H NMR (600 MHz, $CDCl_3$, 283 K): δ = 2.97 (s, 3H, CH_3), 3.00 (d, J=0.4 Hz, 3H, CH_3), 3.04 (s, 3H, CH_3), 3.11 (s, 3H, CH_3), 8.35 (s, 1H, CH).

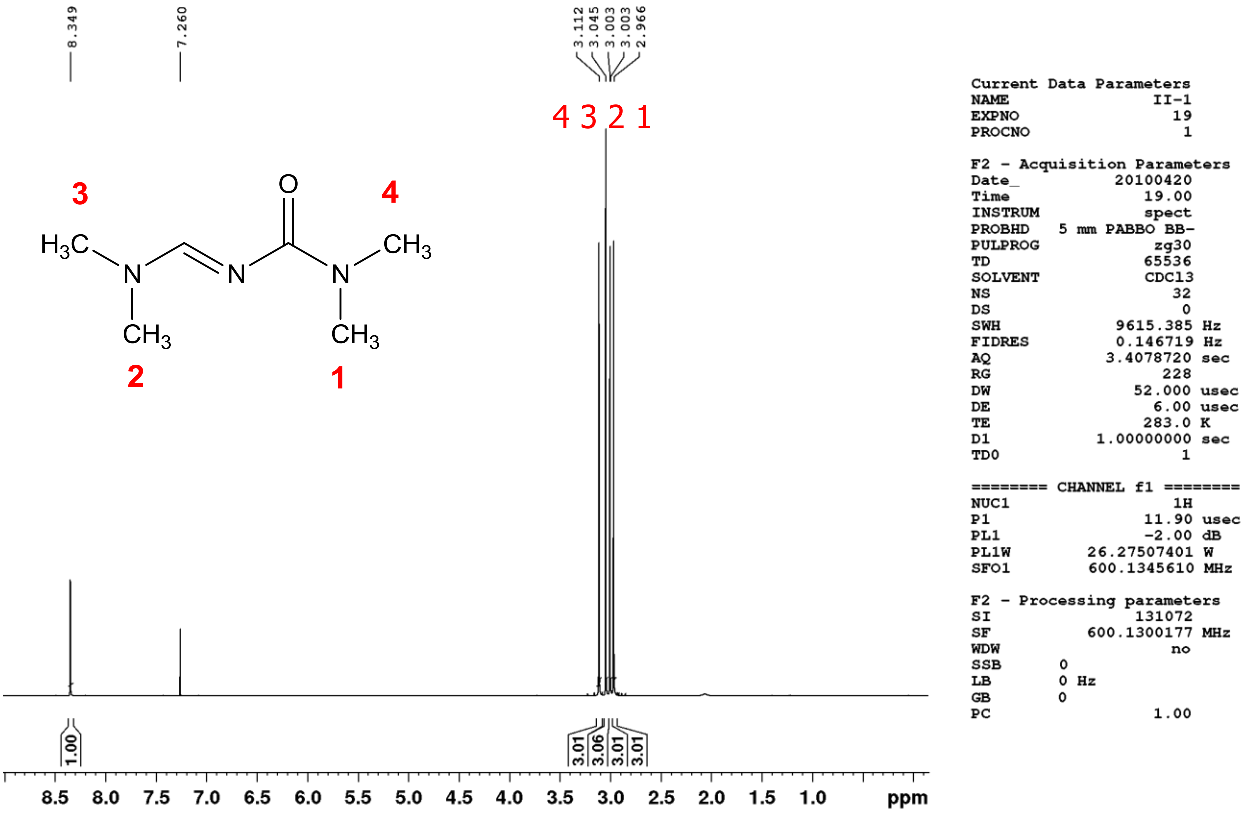

Figure S1. ^{1}H NMR spectrum of compound **1** in $CDCl_3$ at 283K showing the assignment of methyl groups.

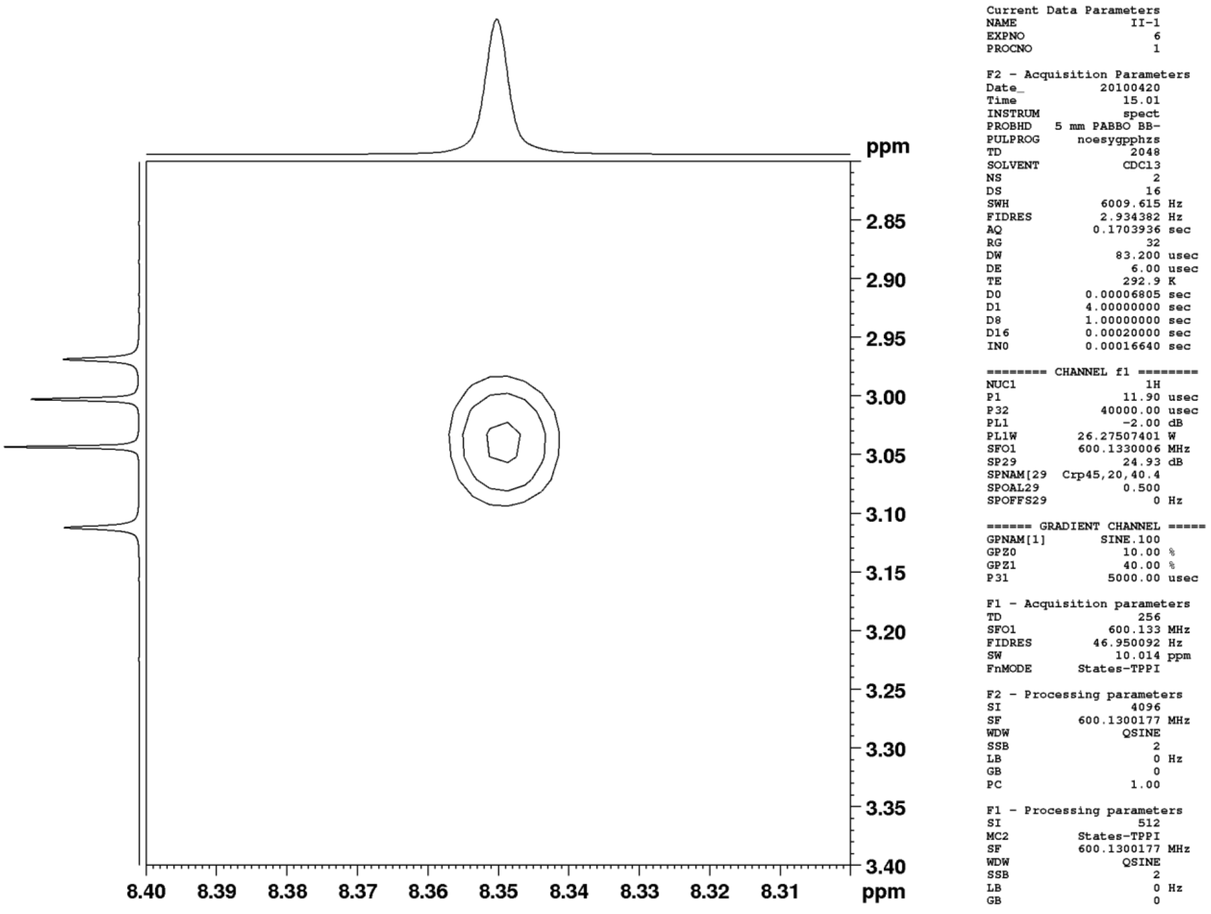

Figure S2. NOESY spectrum of compound **1** in $CDCl_3$ at 293K showing NOE effect between methyl 3 and CH.

^{13}C NMR (150.9 MHz, $CDCl_3$, 293 K): δ = 34.65 (CH_3), 35.65 (CH_3), 36.78 (CH_3), 40.77 (CH_3), 159.87 (CH), 164.30 (C=O).

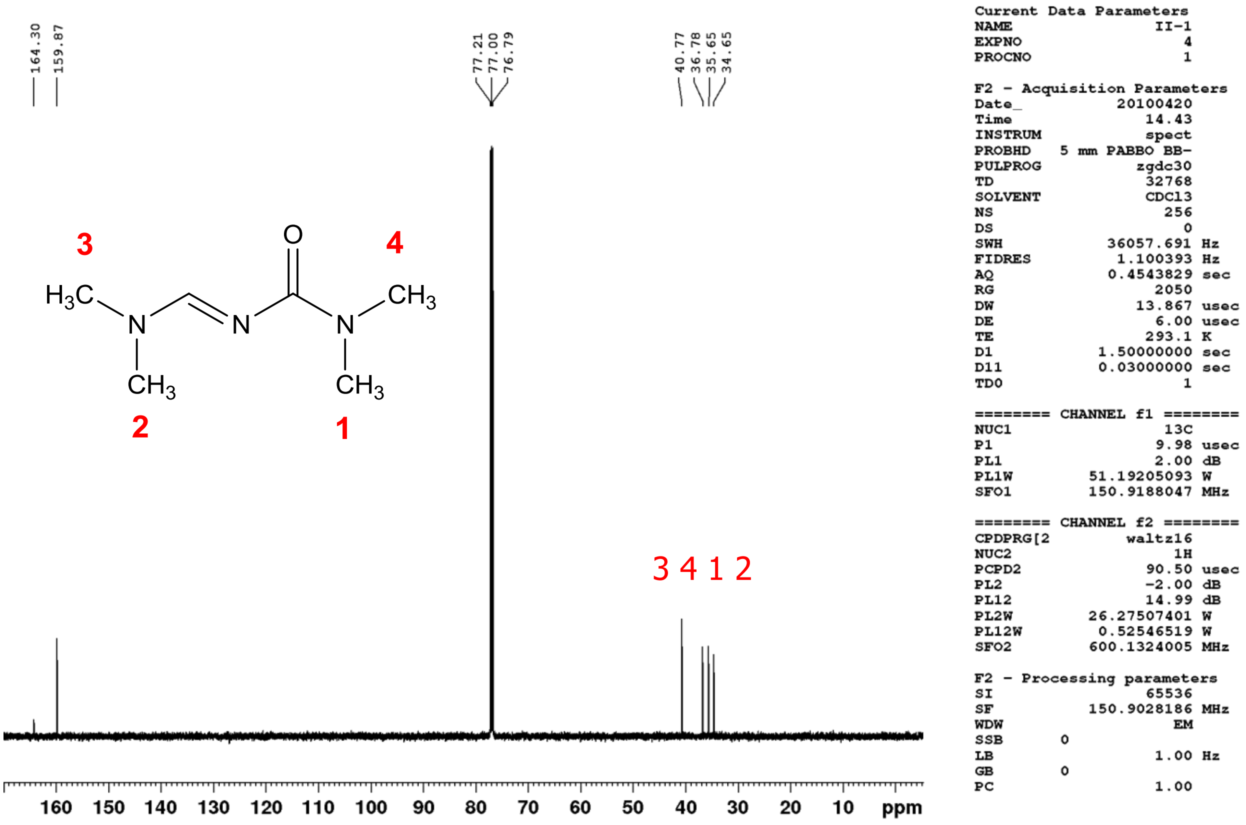

Figure S3. ^{13}C NMR spectrum of compound **1** in $CDCl_3$ at 293K showing the assignment of methyl groups.

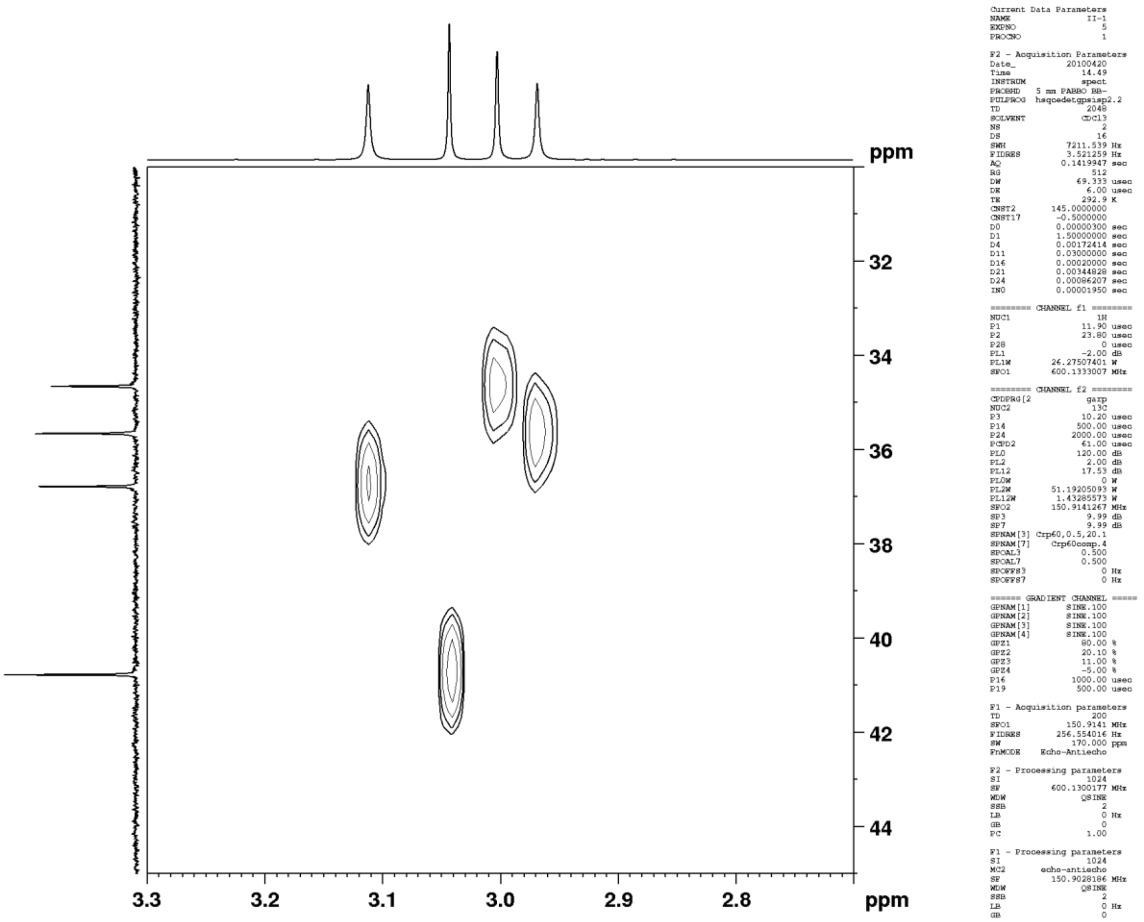

Figure S4. $^{1}H,^{13}C$-HSQC spectrum of compound **1** in $CDCl_3$ at 293K showing the proton-carbon correlations of methyl groups.

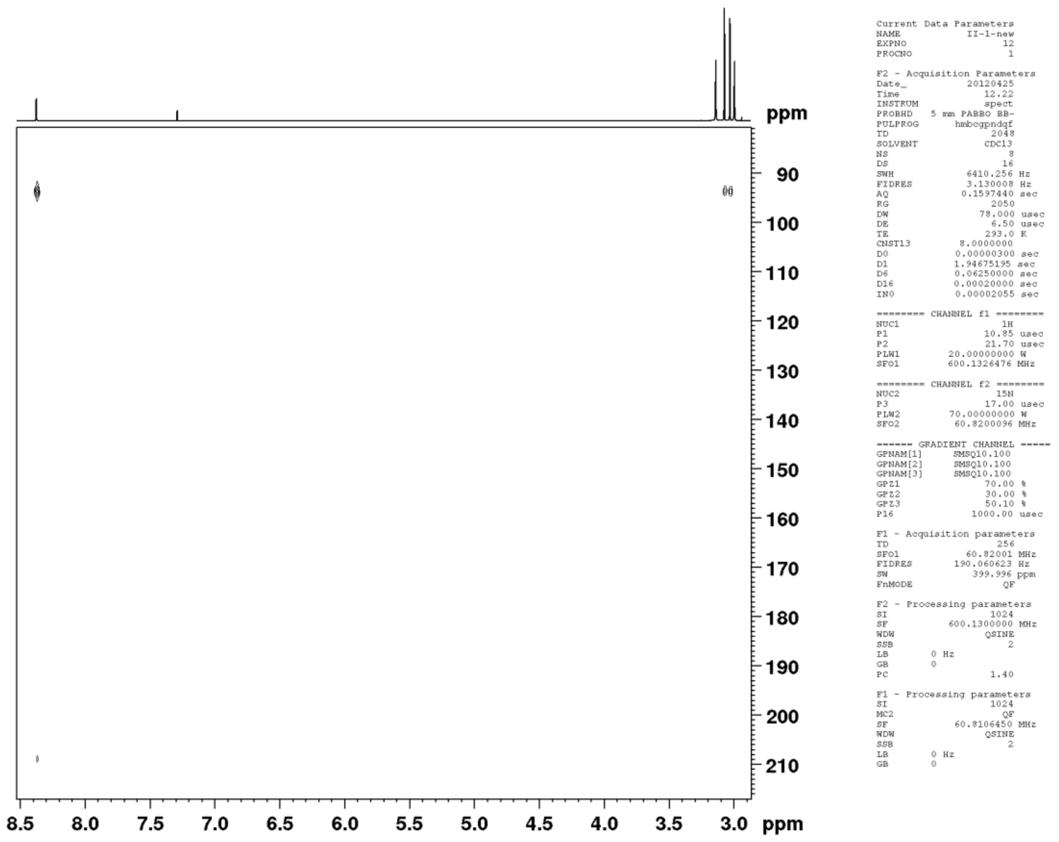

Figure S5. $^{1}H,^{15}N$-HMBC spectrum of compound **1** in $CDCl_3$ at 293K showing the proton-nitrogen correlations between CH proton and of enamine methyl groups.

^{1}H NMR (600 MHz, TCE-d2, 293 K): δ = 2.91 (s, 3H, CH_3), 2.98 (s, 3H, CH_3), 3.00 (s, 3H, CH_3), 3.08 (s, 3H, CH_3), 8.27 (s, 1H, CH).

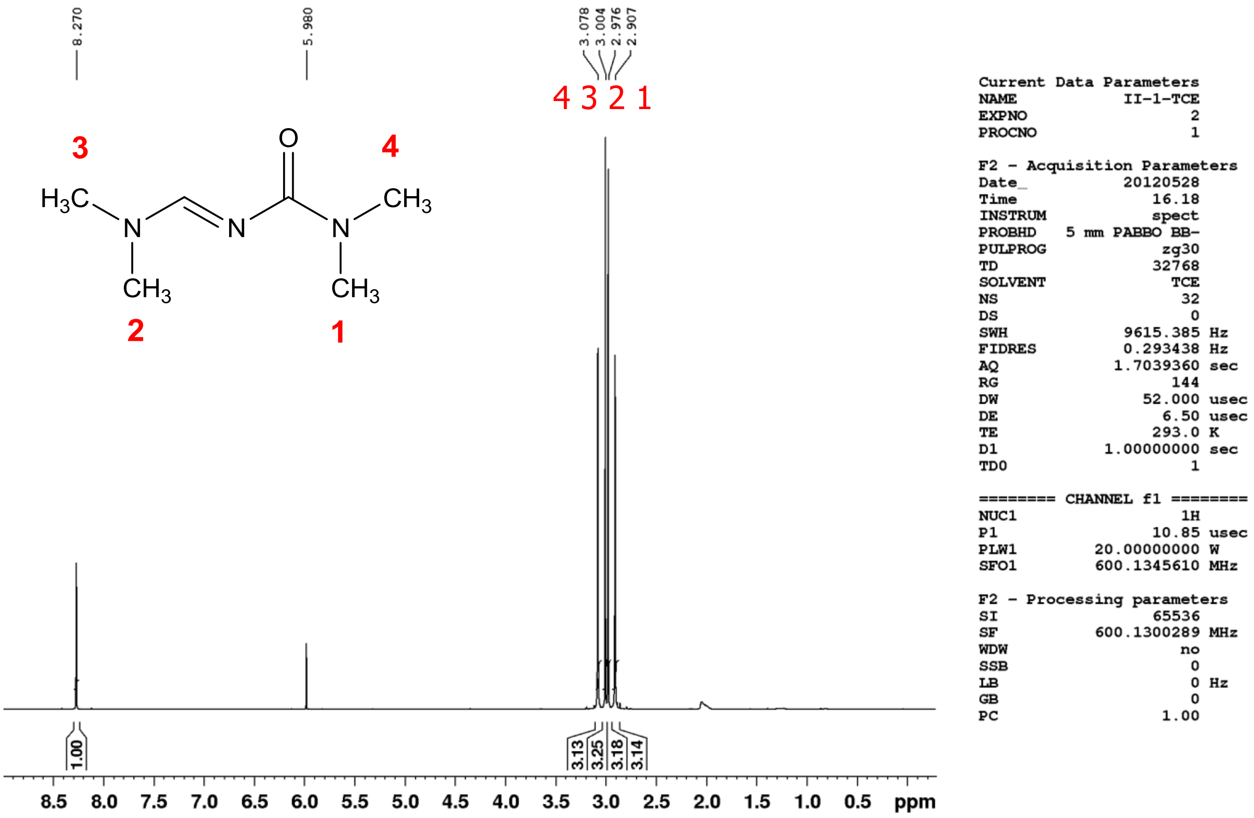

Figure S6. ^{1}H NMR spectrum of compound **1** in TCE-d2 at 293K showing the assignment of methyl groups.

^{13}C NMR (150.9 MHz, TCE-d2, 293 K): δ = 34.56 (CH_3), 35.48 (CH_3), 36.65 (CH_3), 40.64 (CH_3), 159.74 (CH), 163.86 (C=O).

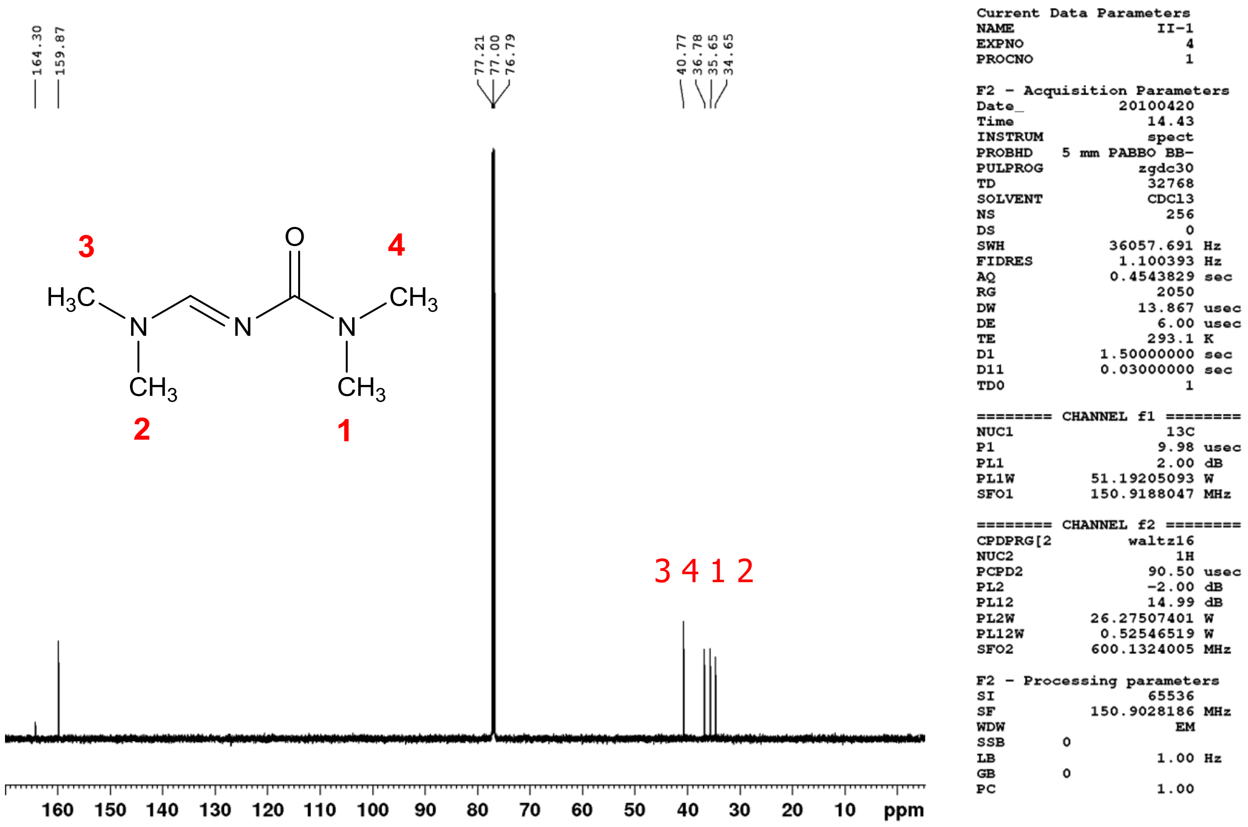

Figure S7. ^{13}C NMR spectrum of compound **1** in TCE-d2 at 293K showing the assignment of methyl groups.

II. Dynamic NMR studies

Compound **1** in $CDCl_3$

^{1}H spectra were recorded on a Bruker II+ 600 instrument (BBO probe) at 600.13 MHz in steps of 5 K between 283 and 328 K (10 mg of **1** in 0.6 ml $CDCl_3$). Temperature calibration was done with B-VT 3000 unit (it was checked and calibrated with methanol and ethylene glycol reference samples). ^{1}H NMR spectra were acquired using a spectral width of 10 kHz, an acquisition time of 1.7 s and 32 scans, zerofilled to 64k datapoints (0.15 Hz per point) and processed without apodization. Peaks were fitted to a Lorentzian lineshape.

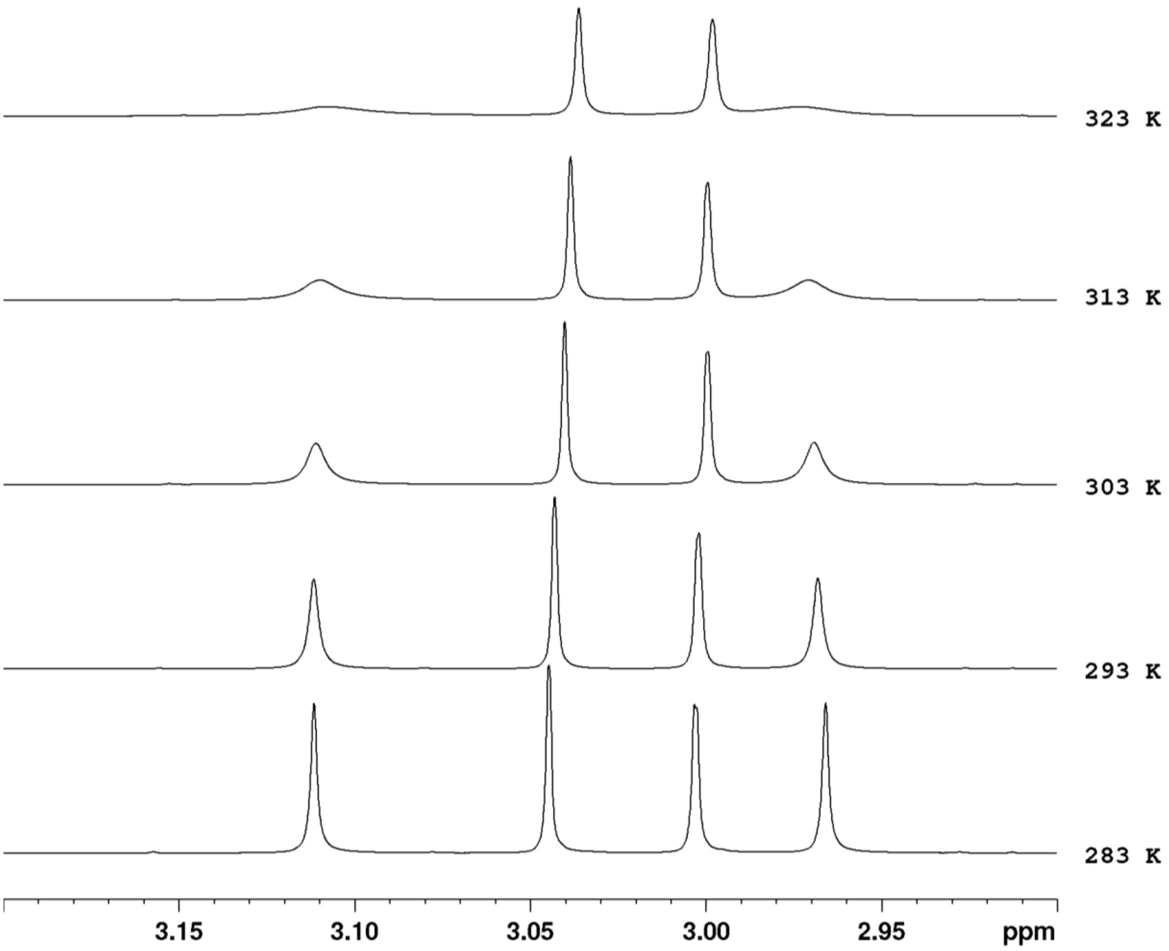

Figure S8. Stacked plots of ^{1}H NMR spectra of compound **1** in $CDCl_3$ at different temperatures.

^{1}H EXSY spectra were recorded on a BBO probe in steps of 5 K between 283 and 323 K. The spectra were acquired using a spectral width of 1.2 kHz, 2048 x 256 complex time domain data points, mixing times in the range of 0.03 to 0.6 s and 2 scans in about 45 min. Linear prediction (32 coefficients and 256 points) in F1 was applied. The spectra were zerofilled to 4096

x 4096 data points and processed with a shifted square sine bell apodization in both dimensions. The populations were obtained by integration of 1D ^{1}H signals and the exchange rates were calculated by program EXSYCalc (MestreLab Research S.L.) from diagonal- and crosspeak integrals.

In the ^{1}H EXSY spectra of **1** the intensity of following peaks were calculated by volume integration:

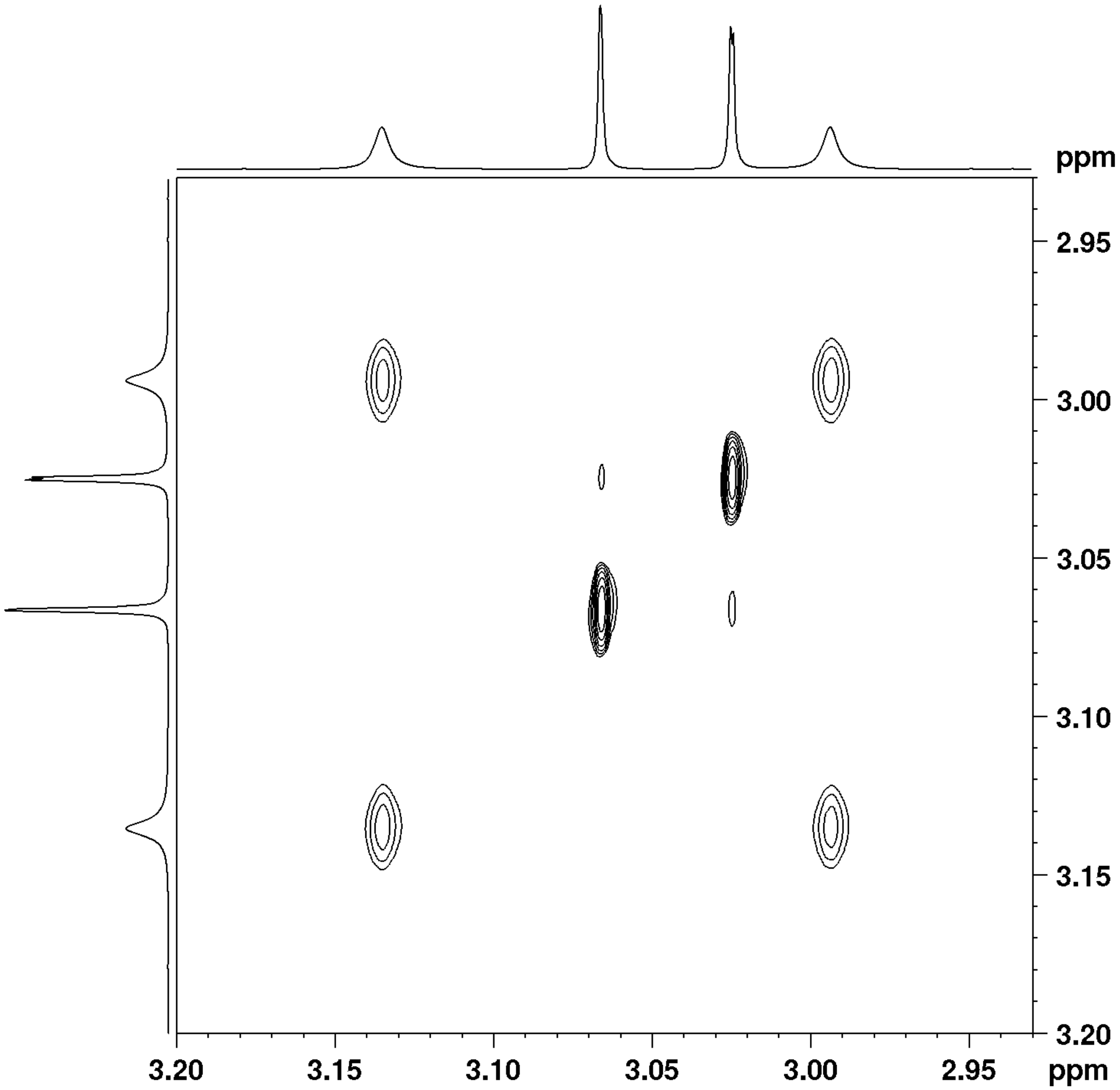

Figure S9. EXSY spectrum of complex **1** in $CDCl_3$ at 303K using mixing time of 0.2 s.

Errors analysis

The total errors ***E<u>tot</u>***, quoted in activation parameters tables are calculated according to the expression $E_{tot} = \sqrt{E_S^2 + E_{kT}^2}$, where $\boldsymbol{E_S}$ is the statistical error based on scattering of the data in the Eyring plot while $\boldsymbol{E_{kT}}$ is computed using error propagation equations as derived by Binsch [G. Binsch, in ***Dynamic NMR Spectroscopy,*** edited by **L.** M. Jackman and F. A. Cotton, **p.** 45. Academic Press, New York (1975)] and Heinzer and Oth [J. Heinzer and J. F. M. Oth, ***Helv. Chim.Acta*** 64, 258 (1981)], in which errors due to both the calculated rate constants and the measured temperature are taken into account. The absolute temperature errors were assumed to be $\boldsymbol{\sigma T}$ = ±0.5 K and the maximum relative error in rate constants was taken to be $\boldsymbol{\sigma k/k}$ = ±10%.

Table S1. Rate constants of compound **1** calculated from EXSY spectra in $CDCl_3$

T, K	*Amide (1* to *4)*,s^{-1}	*Enamine (2* to *3)*,s^{-1}
283	1.3178	
288	2.1569	
293	3.3940	0.0710
298	5.5307	0.1299
303	8.4095	0.2242
308	13.0002	0.3778
313		0.6249
318		1.0307
323		1.7847

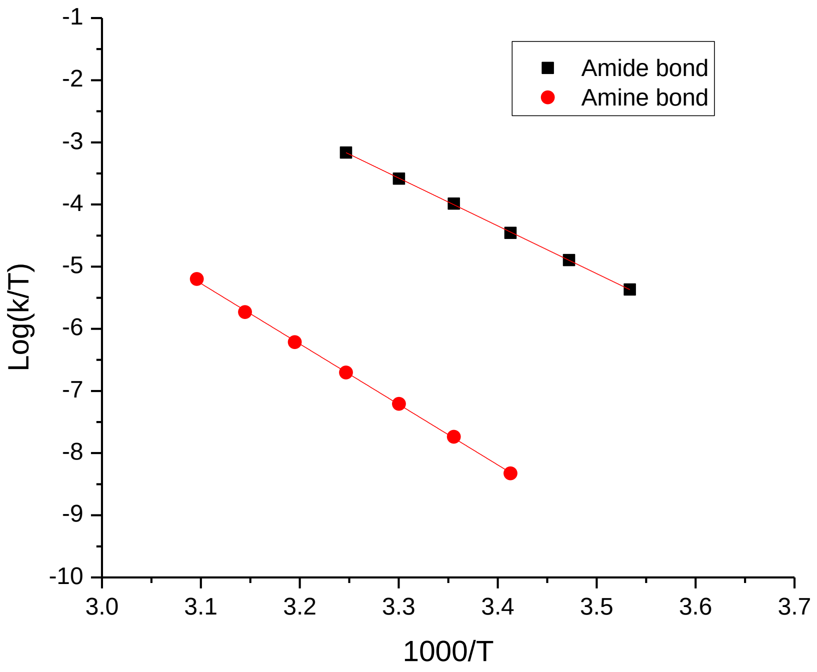

Figure S10. Eyring plot of rate constants of restricted rotation around amide and enamine bond in $CDCl_3$

Table S2. Experimental Activation Parameters for the Exchange Processes of complex **1** in $CDCl_3$

Exchange	$\Delta H^{\neq}$(298K)	$\Delta S^{\neq}$(298K)	$\Delta G^{\neq}$(298K)	R^2
Amide bond	15.3 ± 1.1	-3.9 ± 3.7	16.43 ± 0.07	0.9999
Enamine bond	19.3 ± 1.0	2.2 ± 2.4	18.65 ± 0.07	0.9998

$\Delta G^{\neq}$ and $\Delta H^{\neq}$ in kcal mol^{-1} and $\Delta S^{\neq}$ in cal mol^{-1} K^{-1}

Compound **1** in TCE-d2

^{1}H spectra were recorded on a Bruker II+ 600 instrument (BBO probe) at 600.13 MHz in steps of 10 K between 263 and 393 K (6.9 mg of **1** and 1 drop anisole in 0.6 ml TCE-d2). Temperature calibration was done with B-VT 3000 unit (it was checked and calibrated with methanol and ethylene glycol reference samples). ^{1}H NMR spectra were acquired using a spectral width of 10 kHz, an acquisition time of 1.7 s and 32 scans, zerofilled to 64k datapoints (0.15 Hz per point) and processed without apodization. Peaks were fitted to a Lorentzian lineshape.

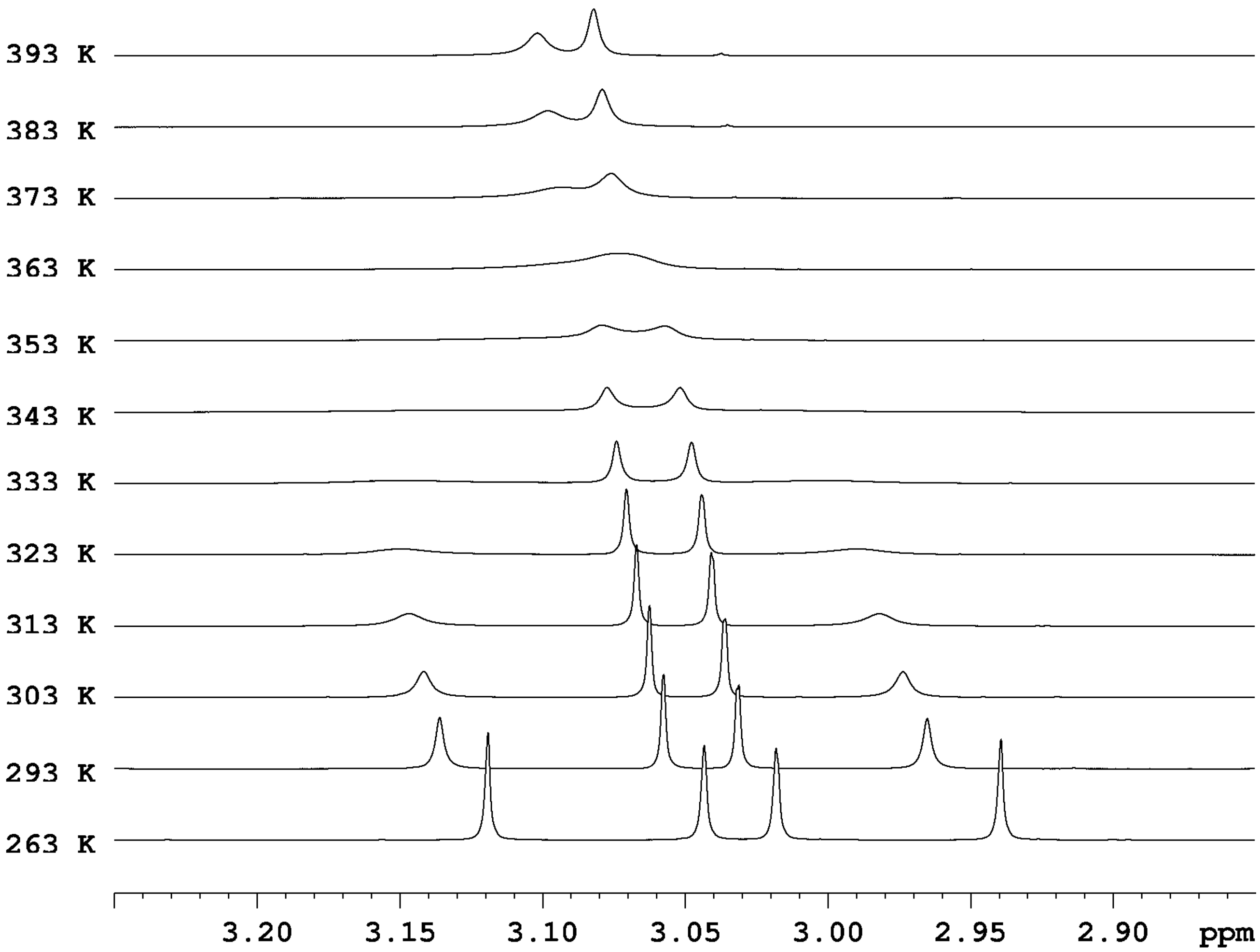

Figure S11. Stacked plots of ^{1}H NMR spectra of compound **1** in TCE-d2 at different temperatures.

^{1}H EXSY spectra were recorded on a BBO probe in steps of 10 K between 263 and 333 K. The spectra were acquired using a spectral width of 0.3 kHz, 2048 x 256 complex time domain data points, mixing times in the range of 0.03 to 1.5 s and 2 scans in about 50 min. Linear

prediction (32 coefficients and 256 points) in F1 was applied. The spectra were zerofilled to 4096 x 4096 data points and processed with a shifted square sine bell apodization in both dimensions. The populations were obtained by integration of 1D ^{1}H signals and the exchange rates were calculated by program EXSYCalc (MestreLab Research S.L.) from diagonal- and crosspeak integrals.

In the ^{1}H EXSY spectra of **1** the intensity of following peaks were calculated by volume integration:

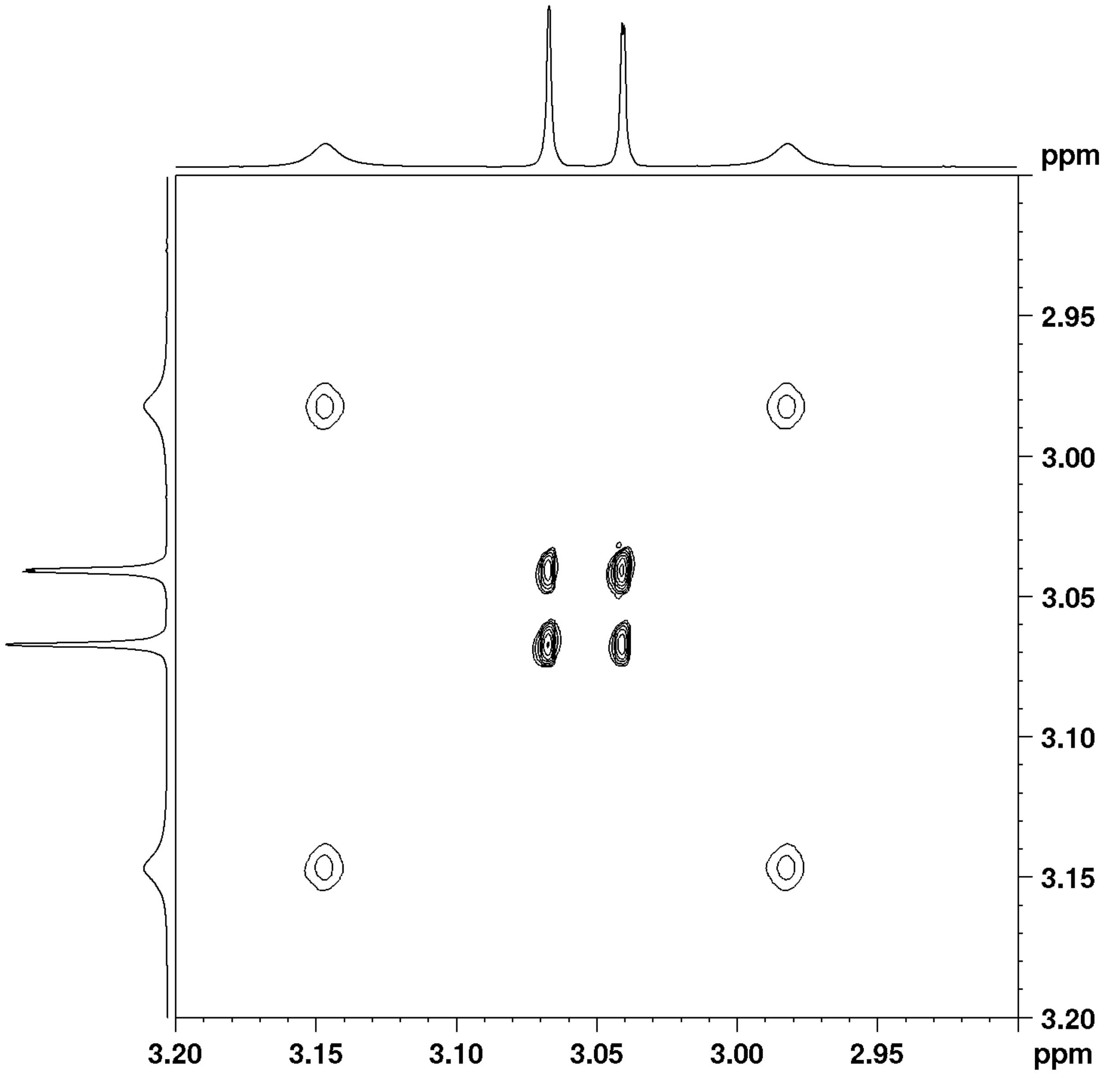

Figure S12. EXSY spectrum of complex **1** in TCE-d2 at 313K using mixing time of 1.0 s.

Errors analysis

The total errors $\underline{\boldsymbol{E_{tot}}}$, quoted in activation parameters tables are calculated according to the expression $E_{tot} = \sqrt{E_S^2 + E_{kT}^2}$, where $\boldsymbol{E_S}$ is the statistical error based on scattering of the data in the Eyring plot while $\boldsymbol{E_{kT}}$ is computed using error propagation equations as derived by Binsch [G. Binsch, in ***Dynamic NMR Spectroscopy,*** edited by **L.** M. Jackman and F. A. Cotton, **p.** 45. Academic Press, New York (1975)] and Heinzer and Oth [J. Heinzer and J. F. M. Oth, ***Helv. Chim.Acta*** 64, 258 (1981)], in which errors due to both the calculated rate constants and the measured temperature are taken into account. The absolute temperature errors were assumed to be $\boldsymbol{\sigma T}$ = ±0.5 K and the maximum relative error in rate constants was taken to be $\boldsymbol{\sigma k/k}$ = ±10%.

Table S3. Rate constants of compound **1** calculated from EXSY spectra in TCE-d2

T, K	*Amide (1* to *4)*,s^{-1}	*Enamine (2* to *3)*,s^{-1}
263	0.1769	
273	0.5291	
283	1.5837	
293	3.8444	0.0465
303	8.9296	0.1721
313	20.2103	0.4920
323		1.5512
333		4.7820

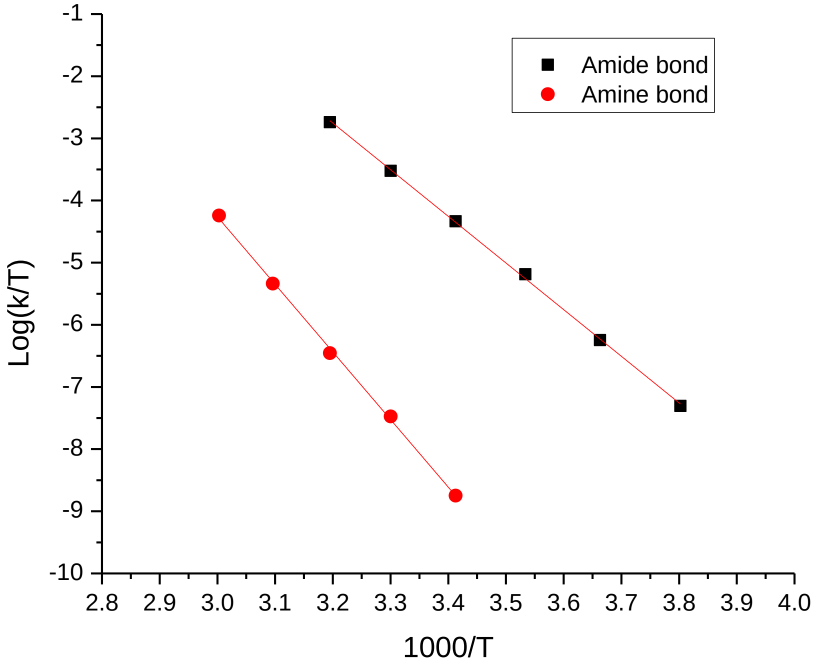

Figure S13. Eyring plot of rate constants of restricted rotation around amide and enamine bond in TCE-d2

Table S4. Experimental Activation Parameters for the Exchange Processes of complex **1** in TCE-d2

Exchange	$\Delta H^{\neq}$(298K)	$\Delta S^{\neq}$(298K)	$\Delta G^{\neq}$(298K)	R^2
Amide bond	14.9 ± 0.5	-5.0 ± 1.9	16.38 ± 0.07	0.9997
Enamine bond	21.6 ± 0.8	9.1 ± 2.5	18.87 ± 0.07	0.9995

$\Delta G^{\neq}$ and $\Delta H^{\neq}$ in kcal mol^{-1} and $\Delta S^{\neq}$ in cal mol^{-1} K^{-1}

Determination of T_1 and T_2 values of the signal of the reference methyl group of anisole in sample of **1** in TCE-d2

Table S5. Measured T_1 and T_2 values of the signal of the reference methyl group of anisole in sample of **1** in TCE-d2 [a]

T, K	T_1 (s)	T_2 (s)
263	1.062 ± 0.003	0.991 ± 0.003
273	1.346 ± 0.002	1.267 ± 0.004
283	1.688 ± 0.002	1.566 ± 0.005
293	2.064 ± 0.001	1.910 ± 0.006
303	2.571 ± 0.001	2.313 ± 0.008
313	2.977 ± 0.001	2.776 ± 0.001
333	3.949 ± 0.001	3.592 ± 0.002

[a] The reported errors for the T_1 and T_2 values are the standard deviations. The temperature errors are in the interval 0.1–0.5 K, based on the variations of the chemical shifts of the ethylene glycol internal thermometer.

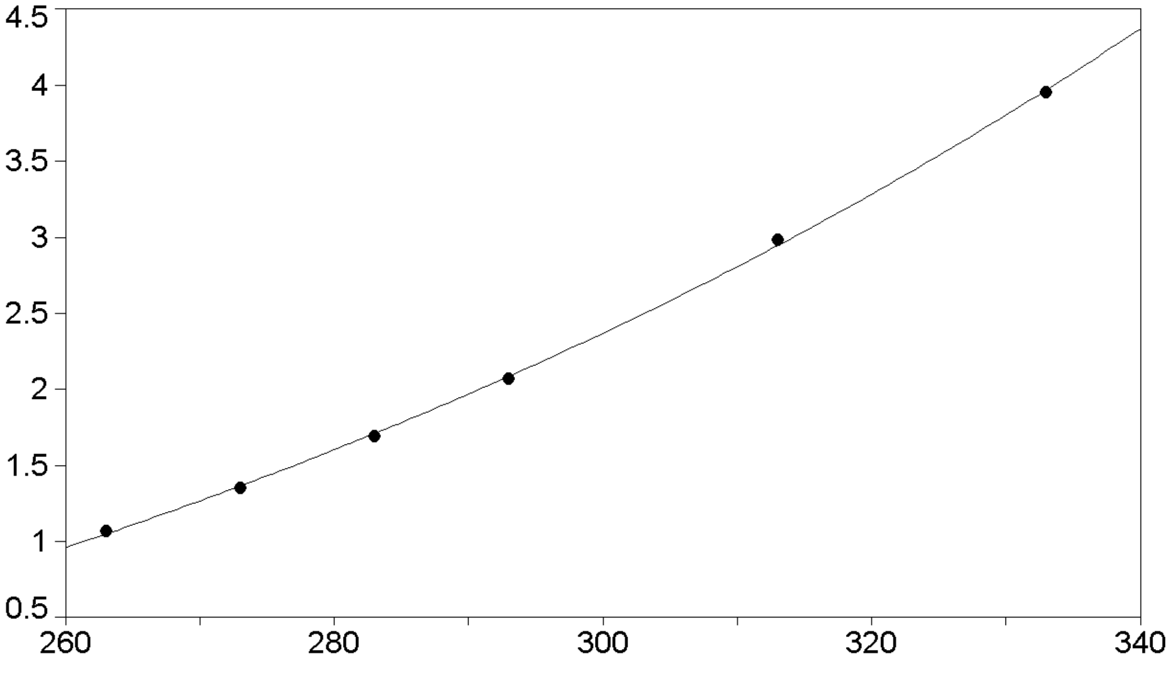

Figure S14. Temperature dependence of T_1 values of the signal of the reference methyl group of anisole in sample of **1** in TCE-d2

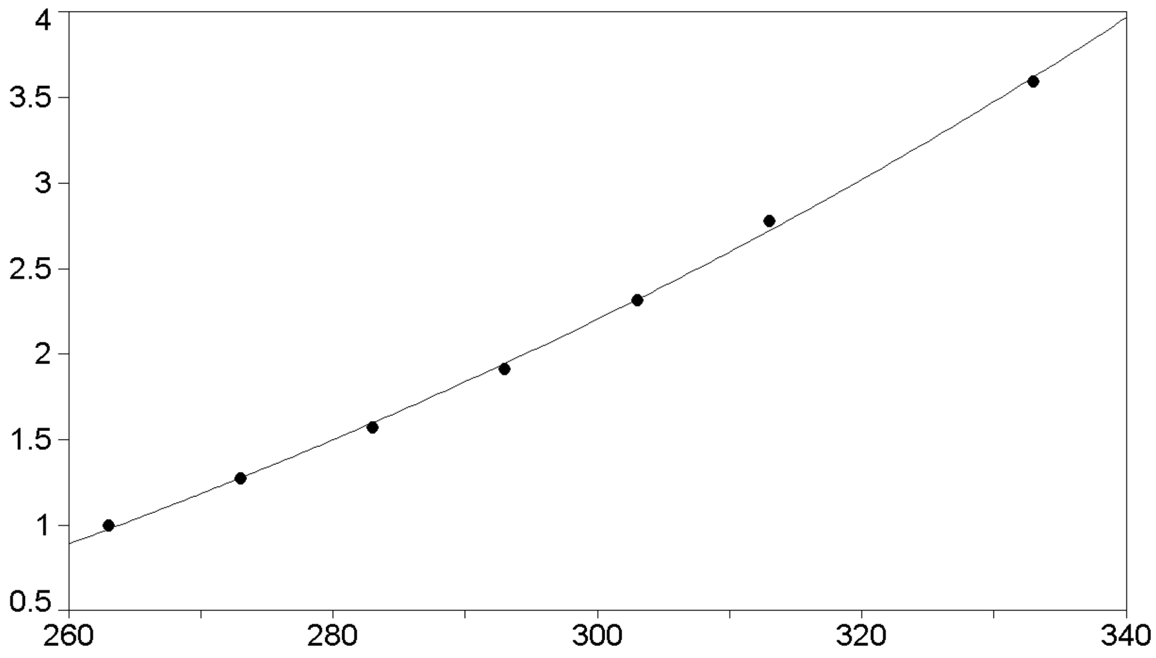

Figure S15. Temperature dependence of T_2 values of the signal of the reference methyl group of anisole in sample of **1** in TCE-d2

Reference deconvolution of compound **1** in TCE-d2

The relaxation rates T_1 and T_2 of the methyl group of the reference compound (anisole) were measured in the temperature range 263–333 K (Table S5) using the *tlir* (inversion–recovery, *T*1) and *cpmg* (Carr–Purcell–Meiboom–Gill, *T*2) programs of the standard Bruker software. The measured T_2 values were smoothed with an exponential temperature function (Fig. S15). The estimated values were used in the reference deconvolution procedure in order to obtain spectra free from inhomogeneity broadening. An automatic correction of noise spikes was applied to the deconvoluted FIDs (Fig. S16).

A

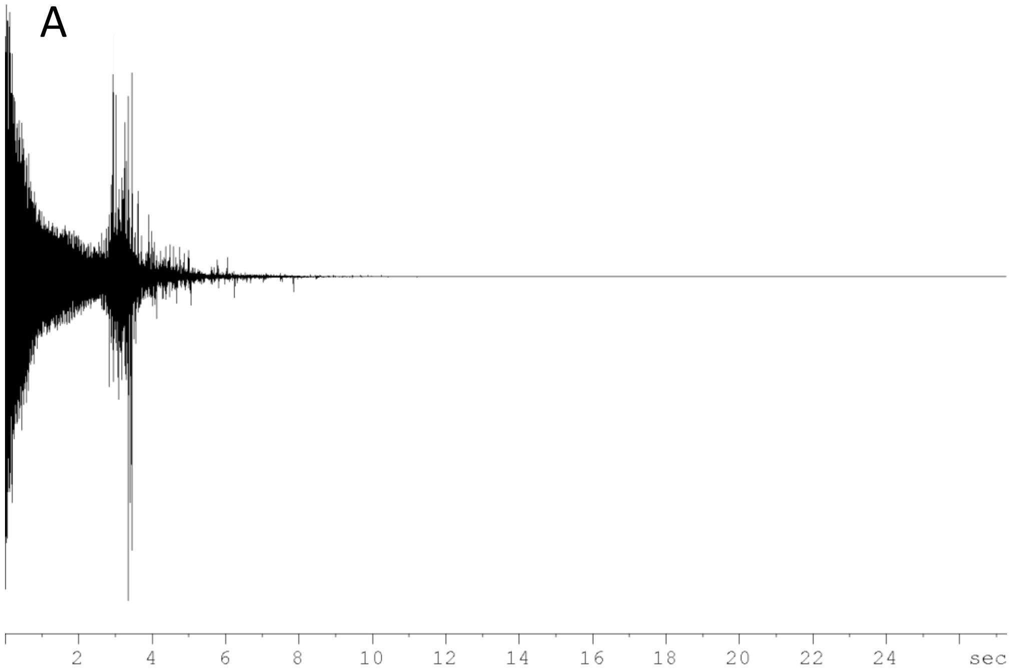
2
4
6
8
10
12
14
16
18
20
22
24
sec

B

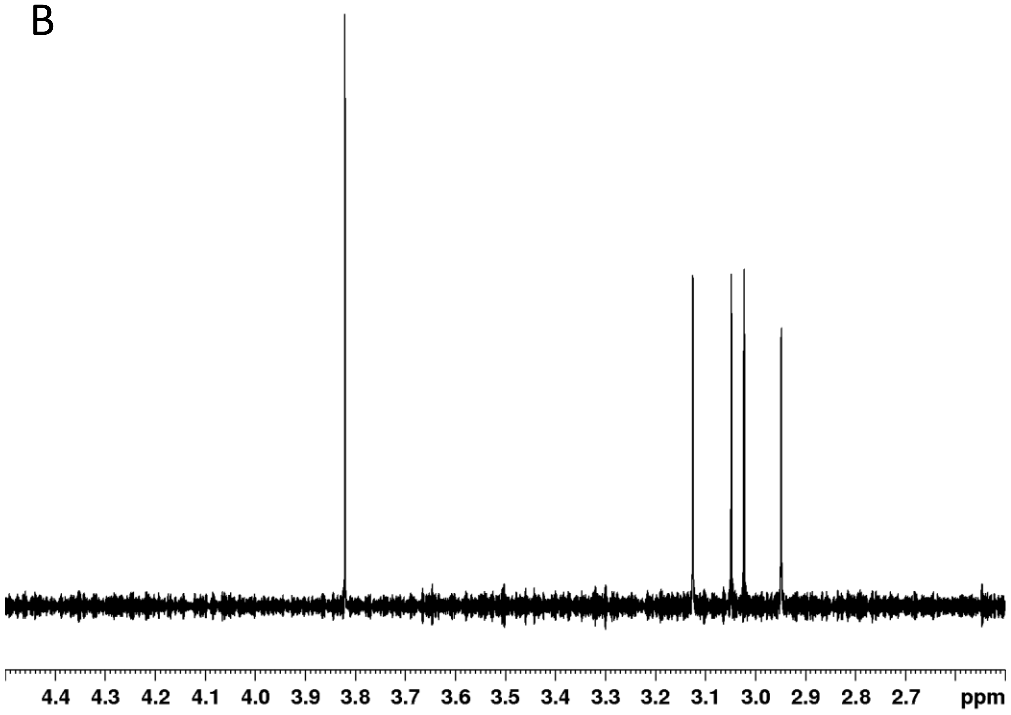
4.4
4.3
4.2
4.1
4.0
3.9
3.8
3.7
3.6
3.5
3.4
3.3
3.2
3.1
3.0
2.9
2.8
2.7
ppm

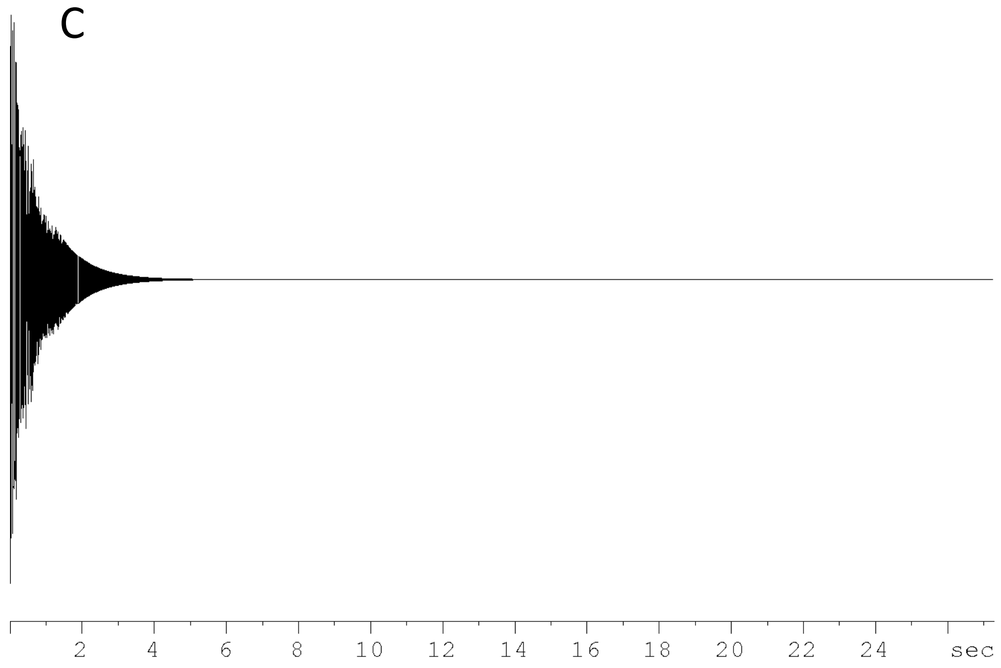

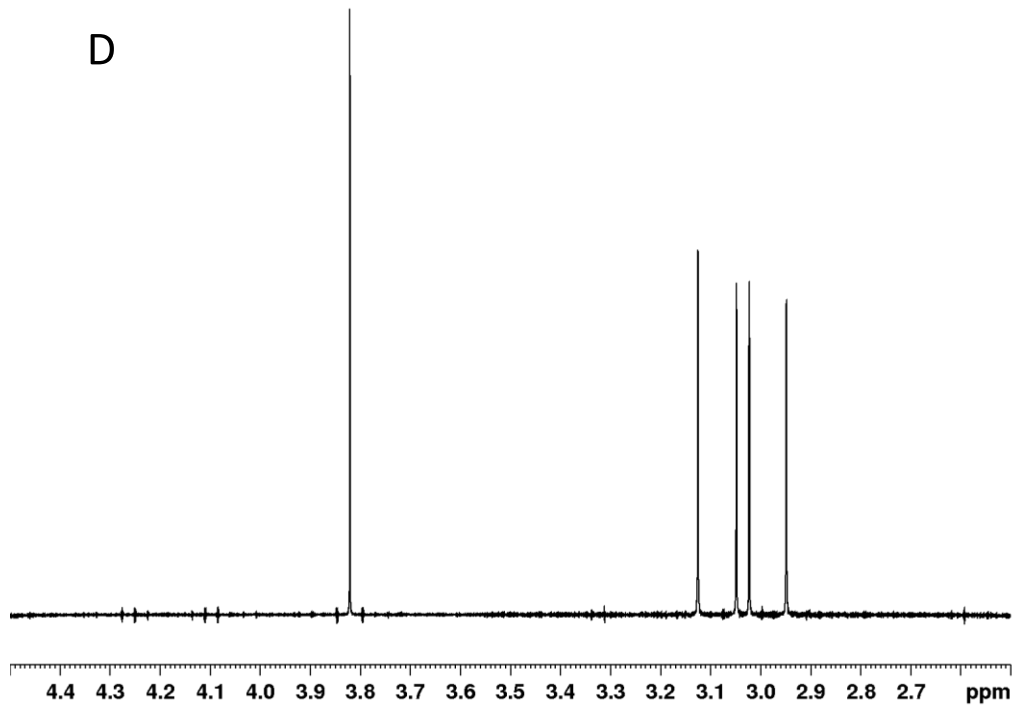

Figure S16. The automated correction of noise spikes of FIDs subjected to reference deconvolution: A – FID at 273K after reference deconvolution; B – FT of FID in A; C – FID in A after application of automatic correction of noise spikes; D – FT of FID in C.

CLSA of compound **1** in TCE-d2

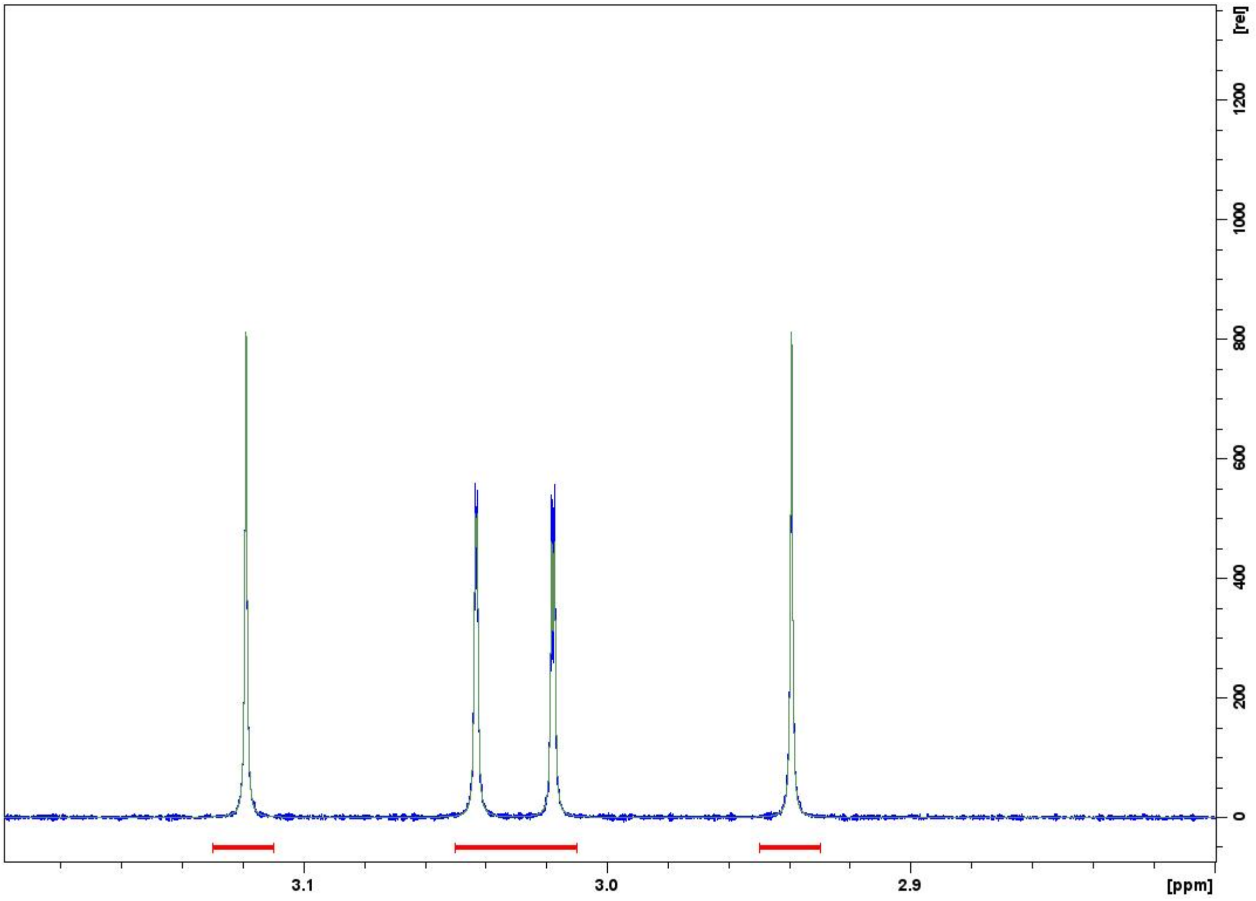

Figure S17. The experimental and the simulated spectra after the iterative parameter refinement of RD spectrum at 263 K of compound **1** in TCE-d2 using dnmr module v.1.1.2 of topspin program.

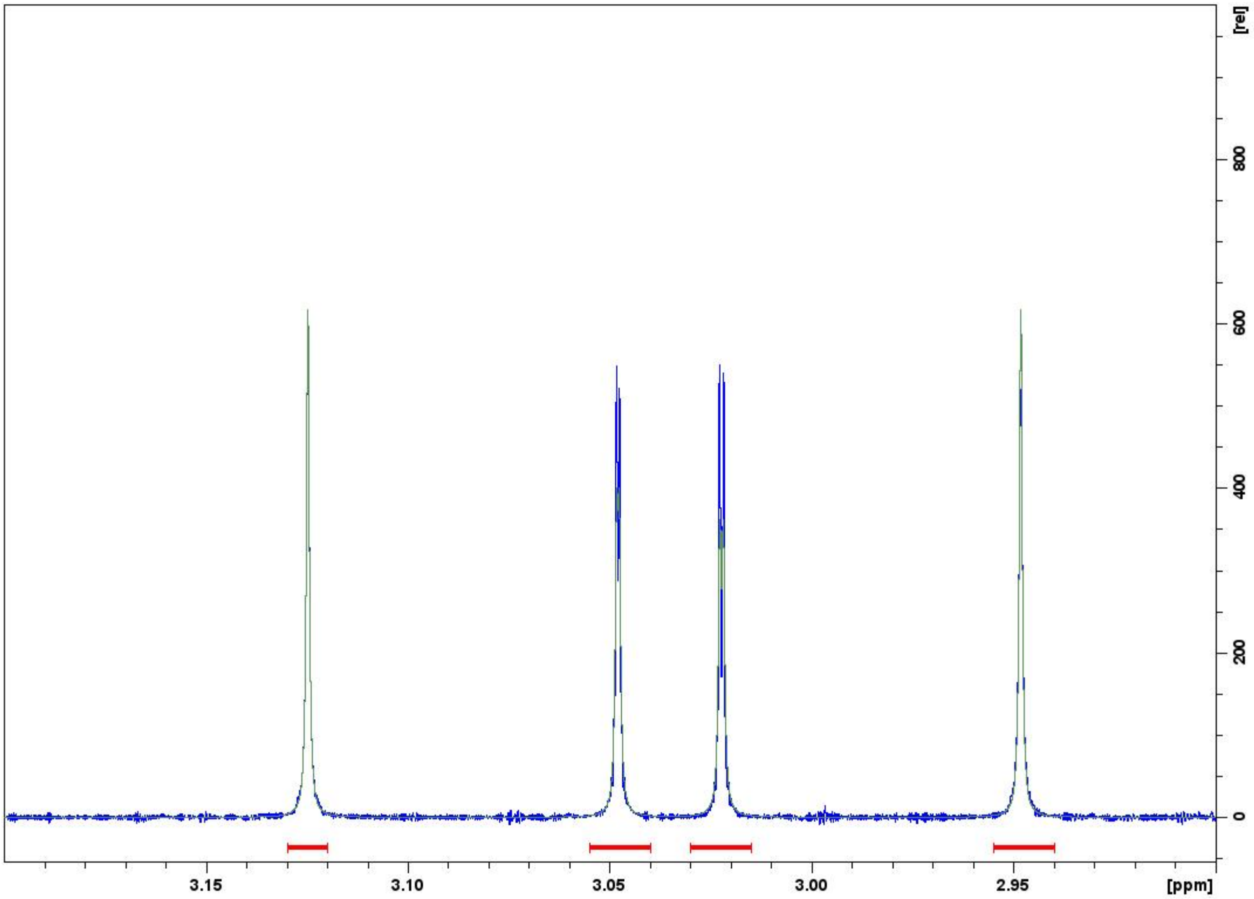

Figure S18. The experimental and the simulated spectra after the iterative parameter refinement of RD spectrum at 273 K of compound **1** in TCE-d2 using dnmr module v.1.1.2 of topspin program.

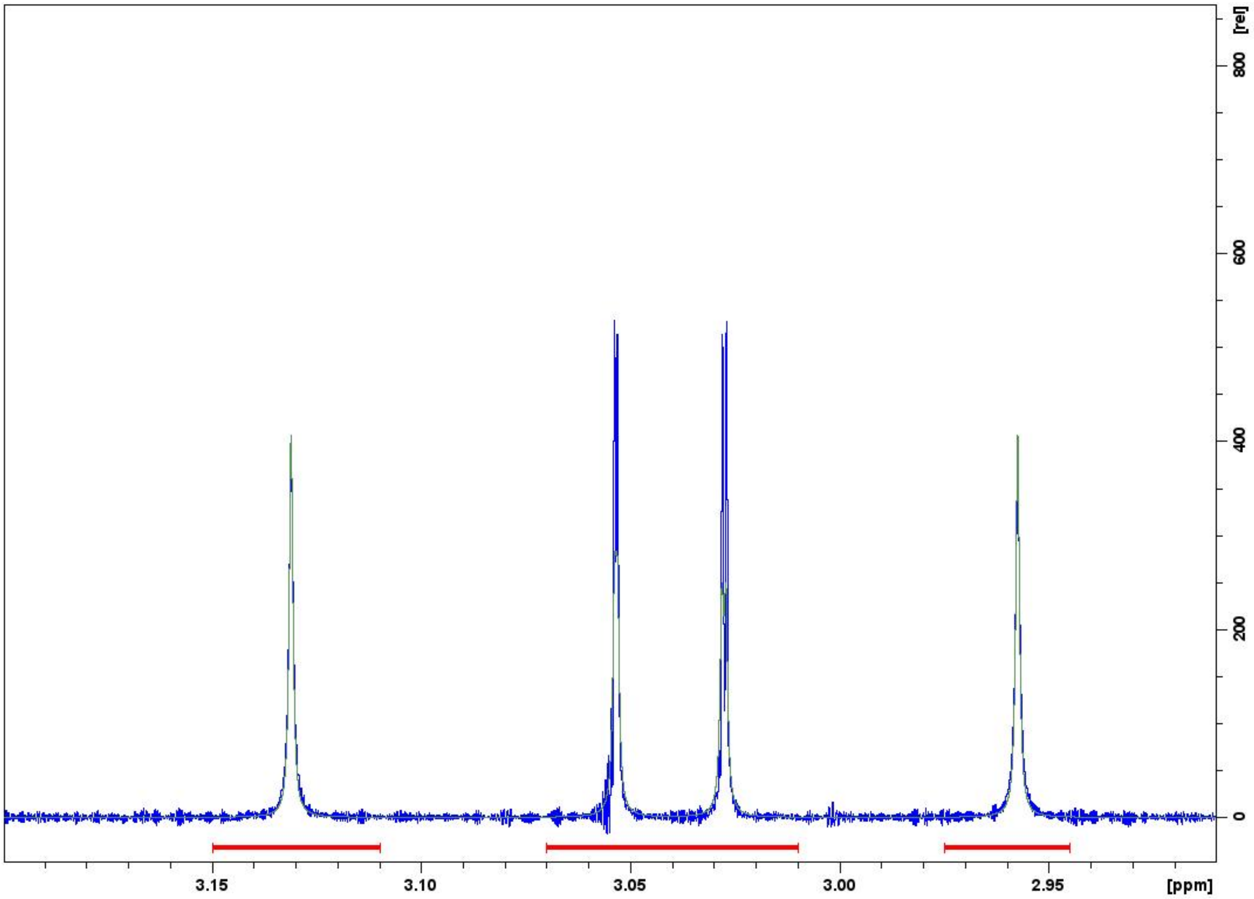

Figure S19. The experimental and the simulated spectra after the iterative parameter refinement of RD spectrum at 283 K of compound **1** in TCE-d2 using dnmr module v.1.1.2 of topspin program.

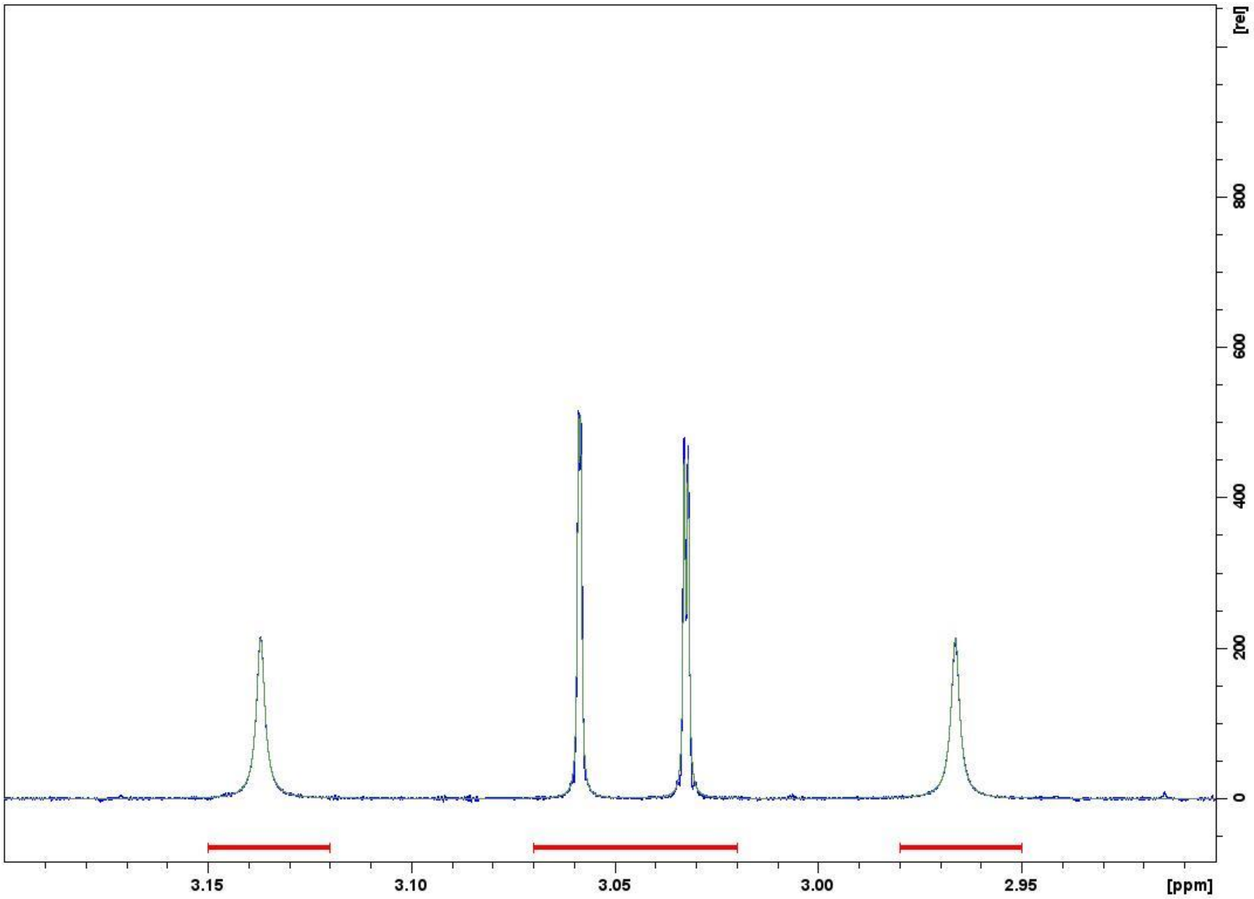

Figure S20. The experimental and the simulated spectra after the iterative parameter refinement of RD spectrum at 293 K of compound **1** in TCE-d2 using dnmr module v.1.1.2 of topspin program.

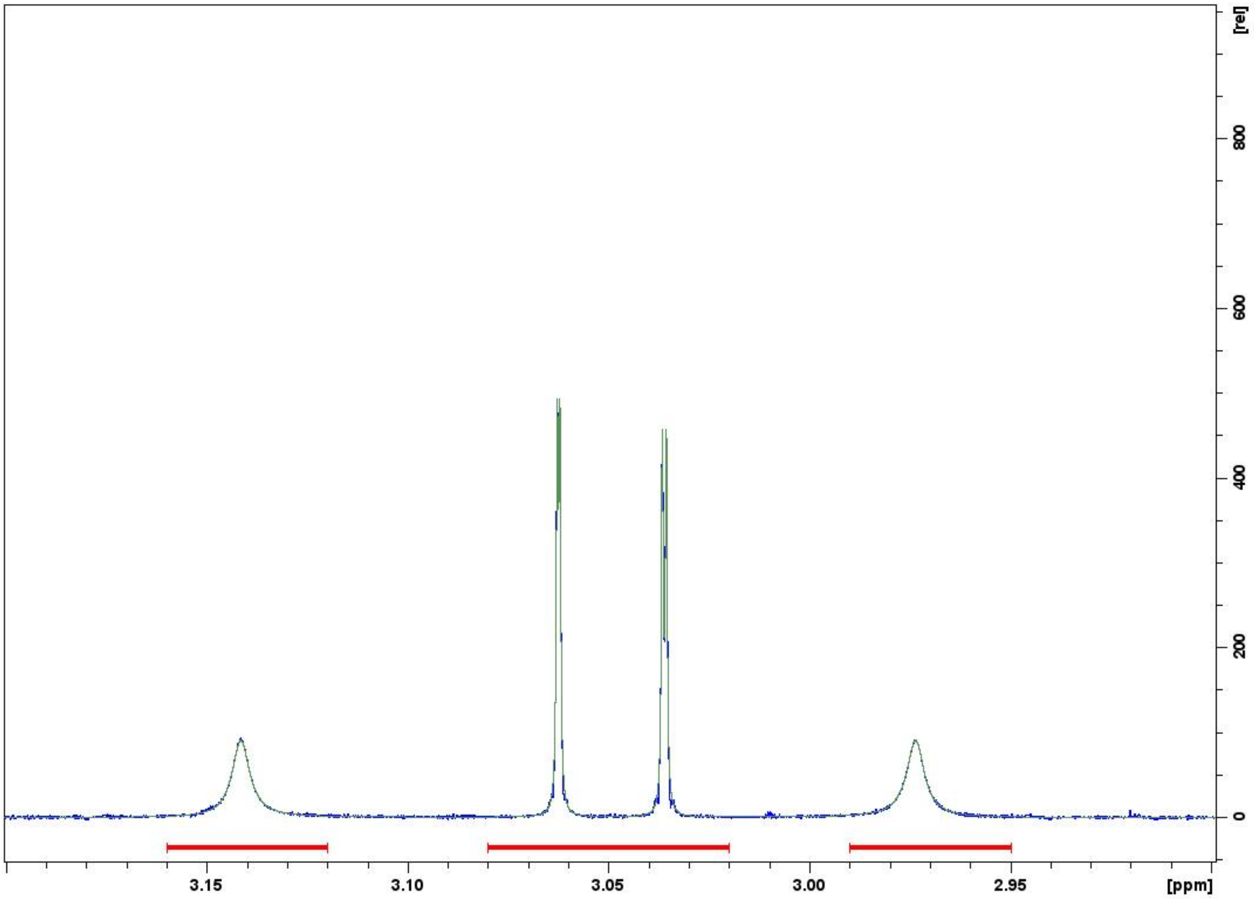

Figure S21. The experimental and the simulated spectra after the iterative parameter refinement of RD spectrum at 303 K of compound **1** in TCE-d2 using dnmr module v.1.1.2 of topspin program.

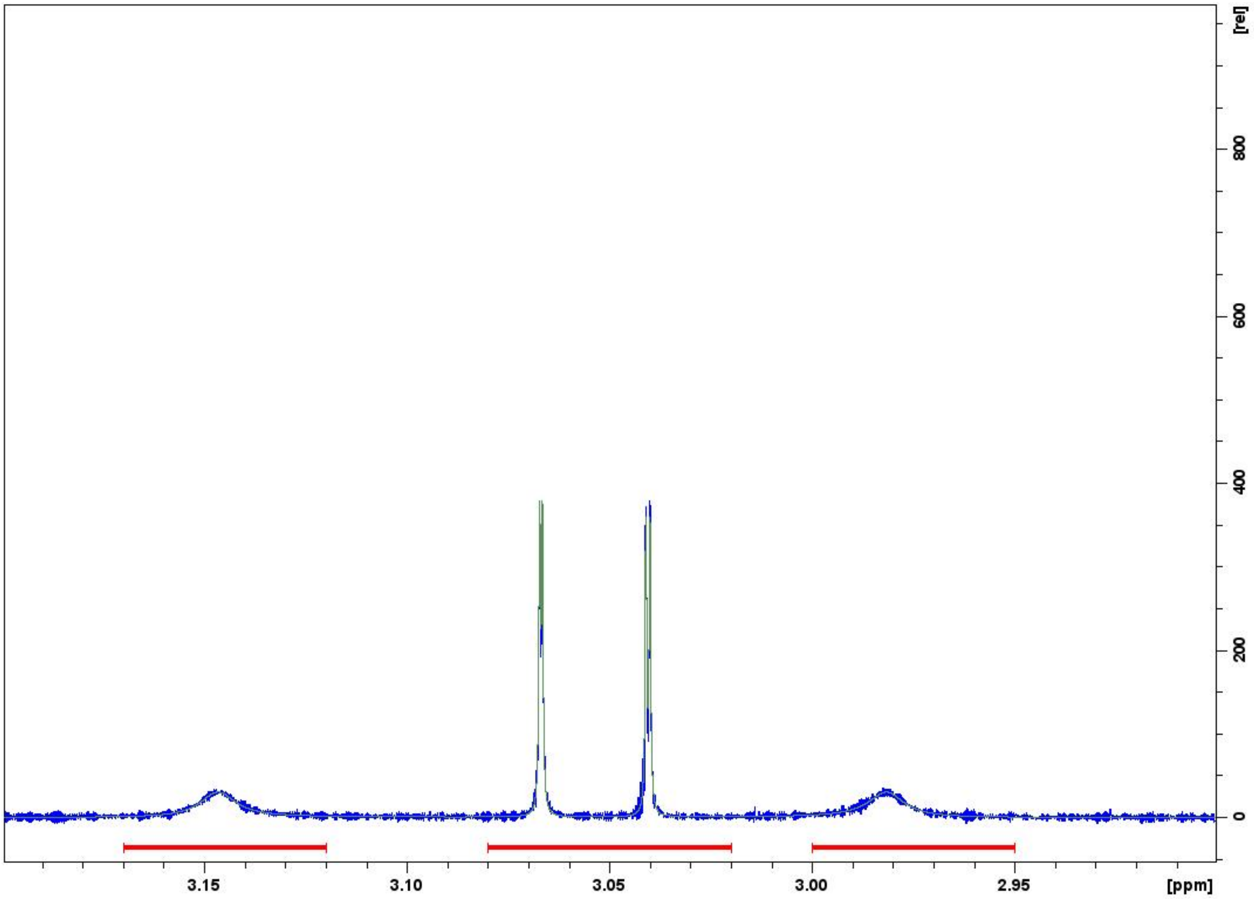

Figure S22. The experimental and the simulated spectra after the iterative parameter refinement of RD spectrum at 313 K of compound **1** in TCE-d2 using dnmr module v.1.1.2 of topspin program.

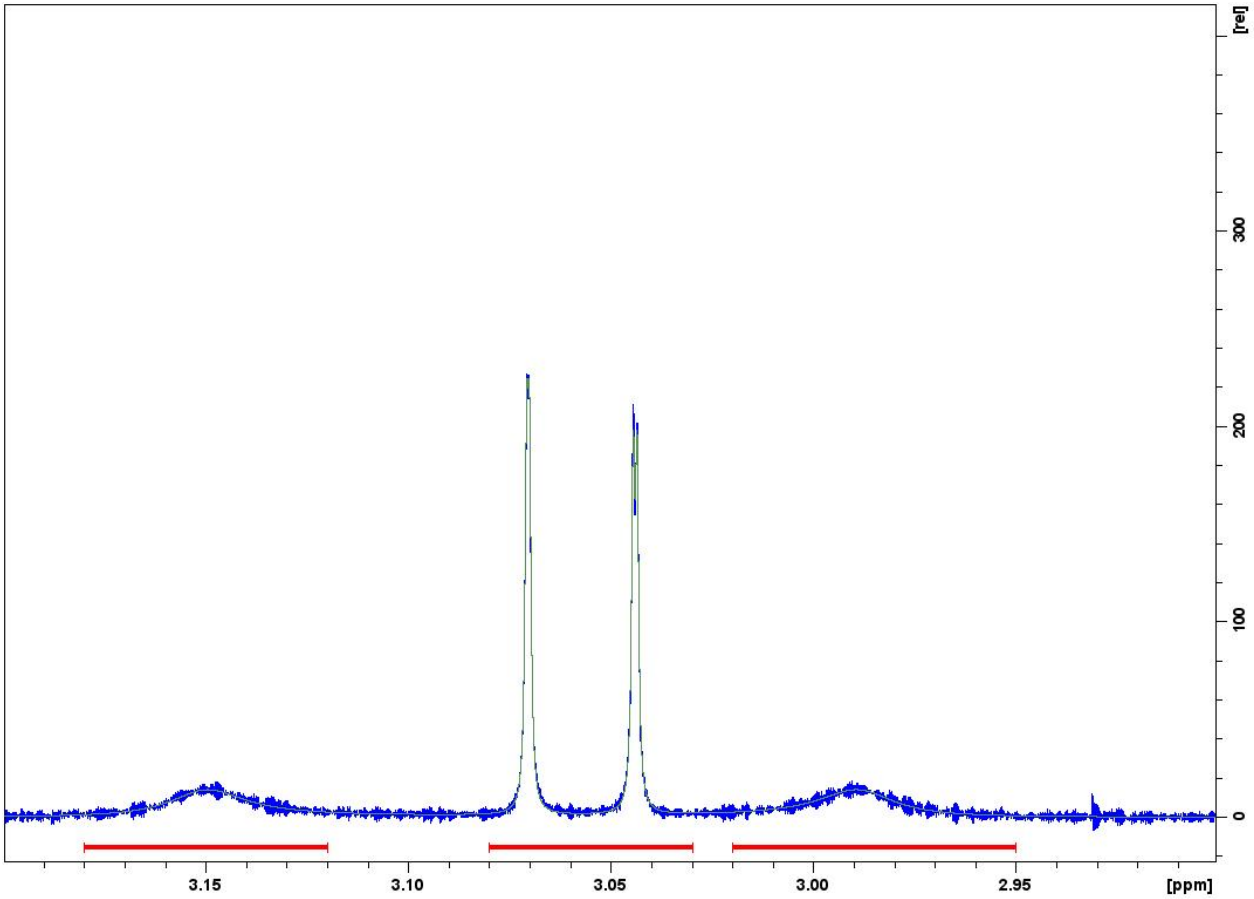

Figure S23. The experimental and the simulated spectra after the iterative parameter refinement of RD spectrum at 323 K of compound **1** in TCE-d2 using dnmr module v.1.1.2 of topspin program.

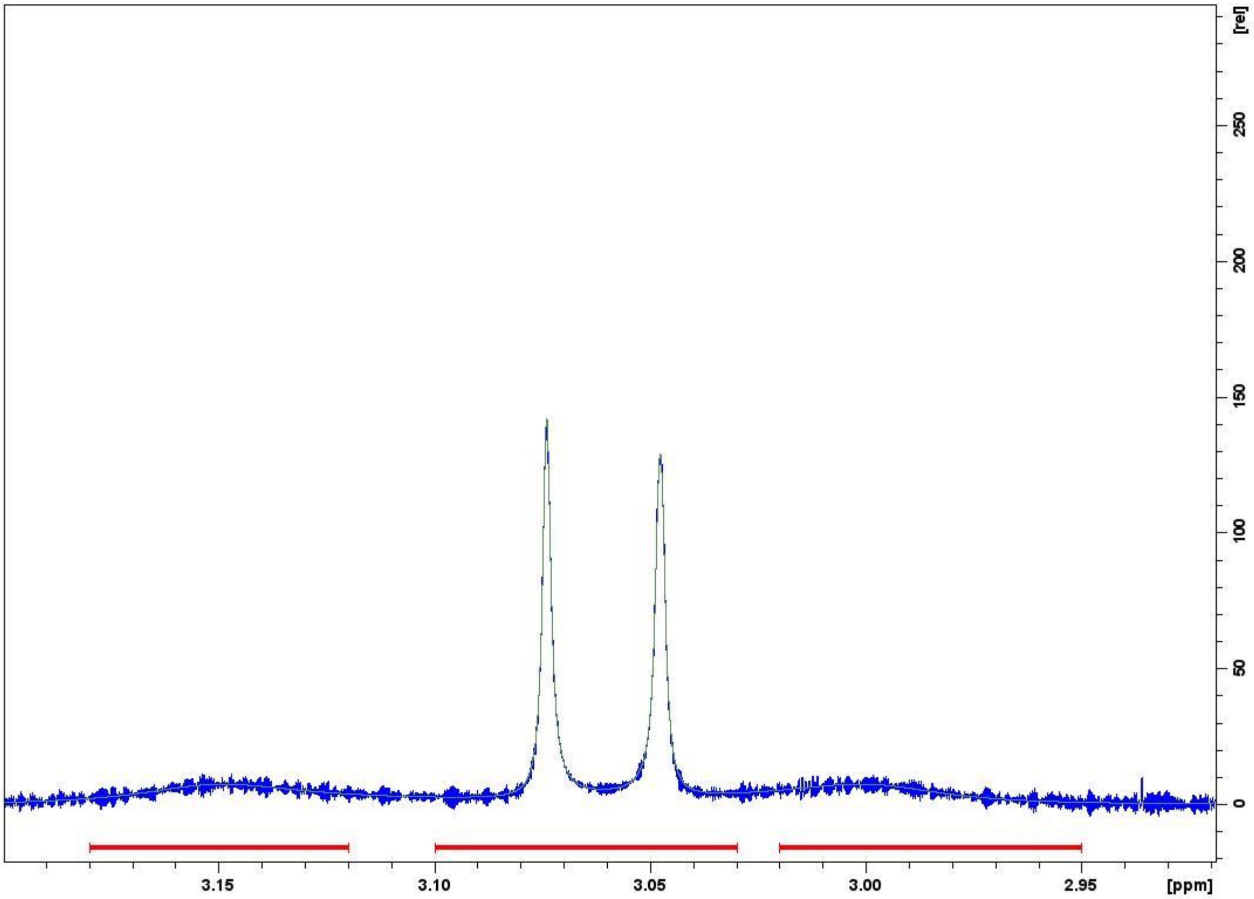

Figure S24. The experimental and the simulated spectra after the iterative parameter refinement of RD spectrum at 333 K of compound **1** in TCE-d2 using dnmr module v.1.1.2 of topspin program.

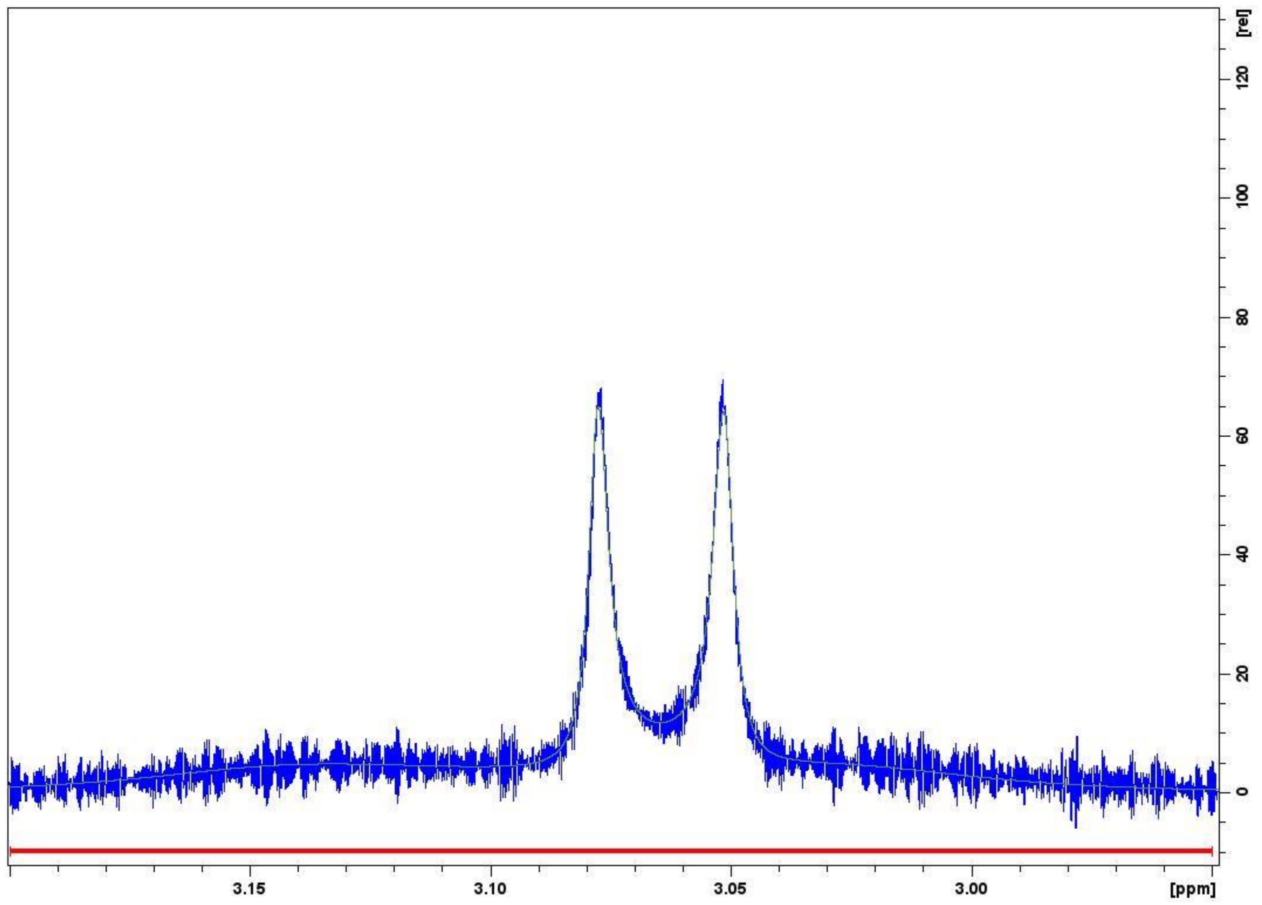

Figure S25. The experimental and the simulated spectra after the iterative parameter refinement of RD spectrum at 343 K of compound **1** in TCE-d2 using dnmr module v.1.1.2 of topspin program.

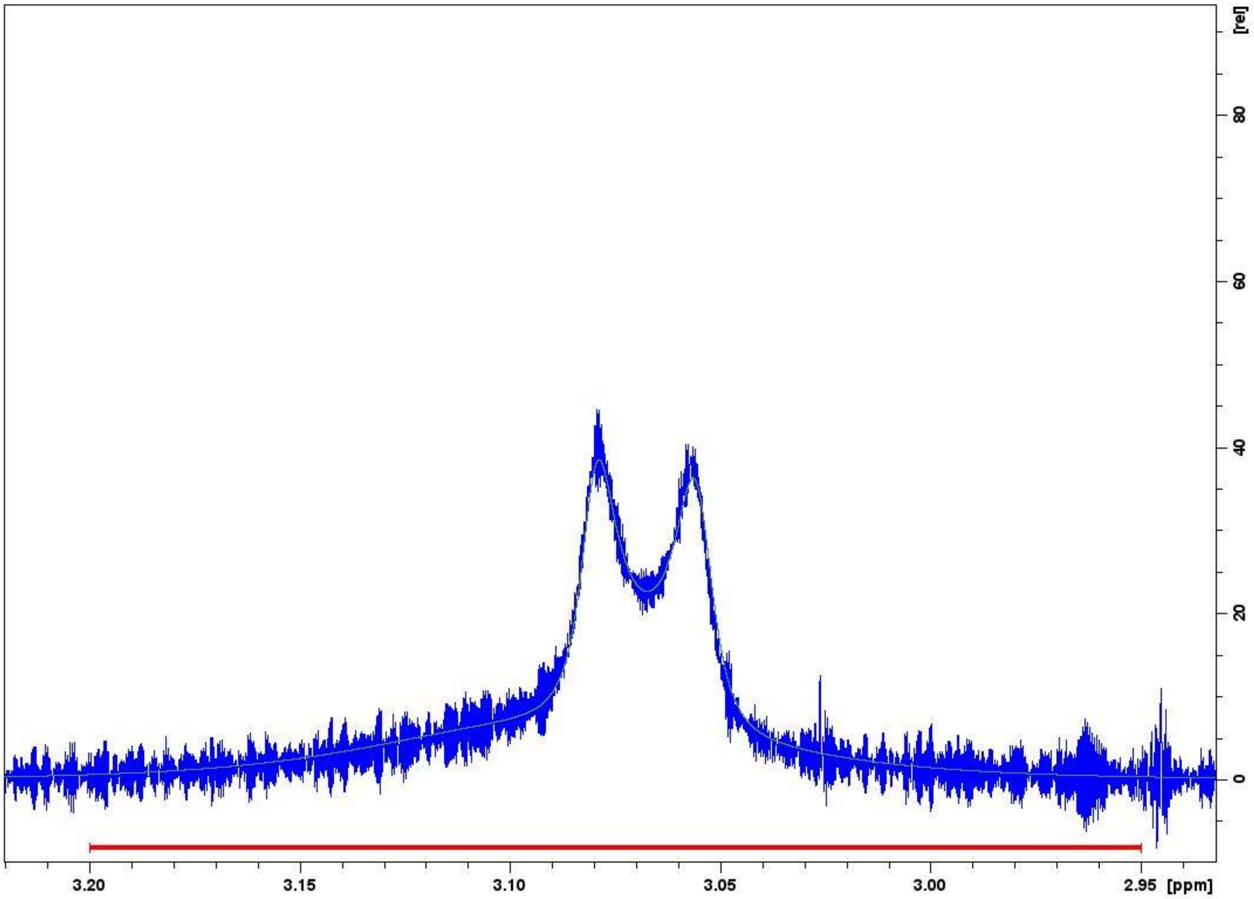

Figure S26. The experimental and the simulated spectra after the iterative parameter refinement of RD spectrum at 353 K of compound **1** in TCE-d2 using dnmr module v.1.1.2 of topspin program.

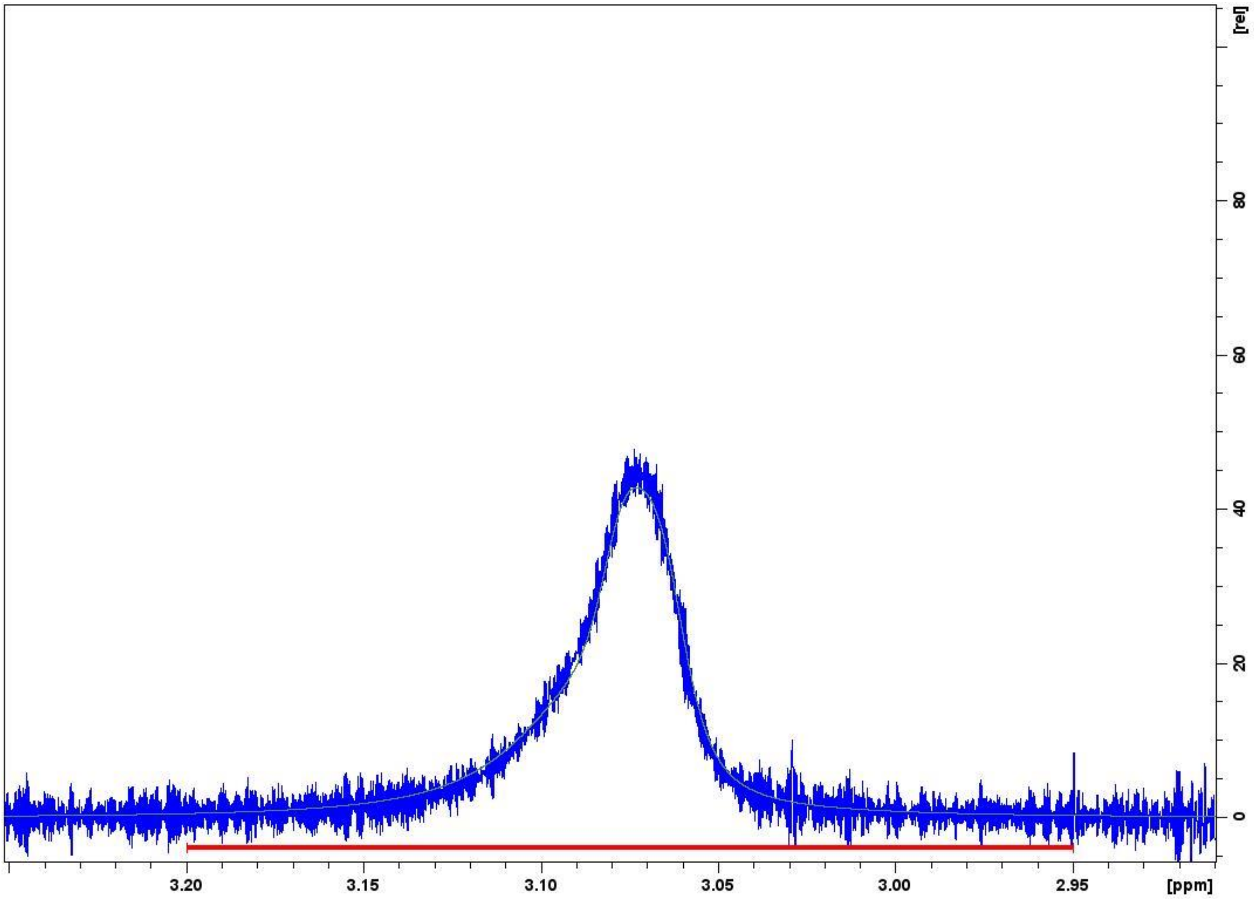

Figure S27. The experimental and the simulated spectra after the iterative parameter refinement of RD spectrum at 363 K of compound **1** in TCE-d2 using dnmr module v.1.1.2 of topspin program.

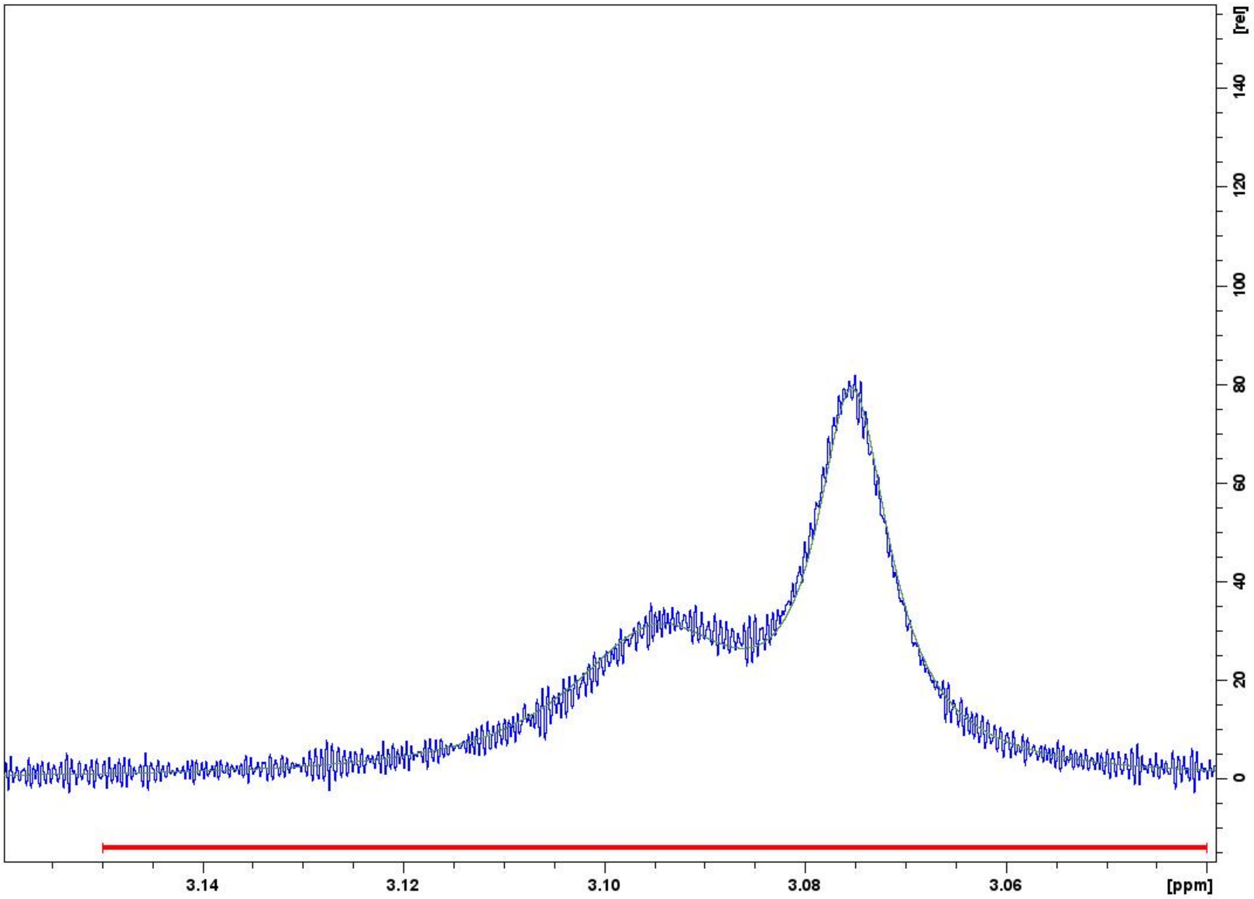

Figure S28. The experimental and the simulated spectra after the iterative parameter refinement of RD spectrum at 373 K of compound **1** in TCE-d2 using dnmr module v.1.1.2 of topspin program.

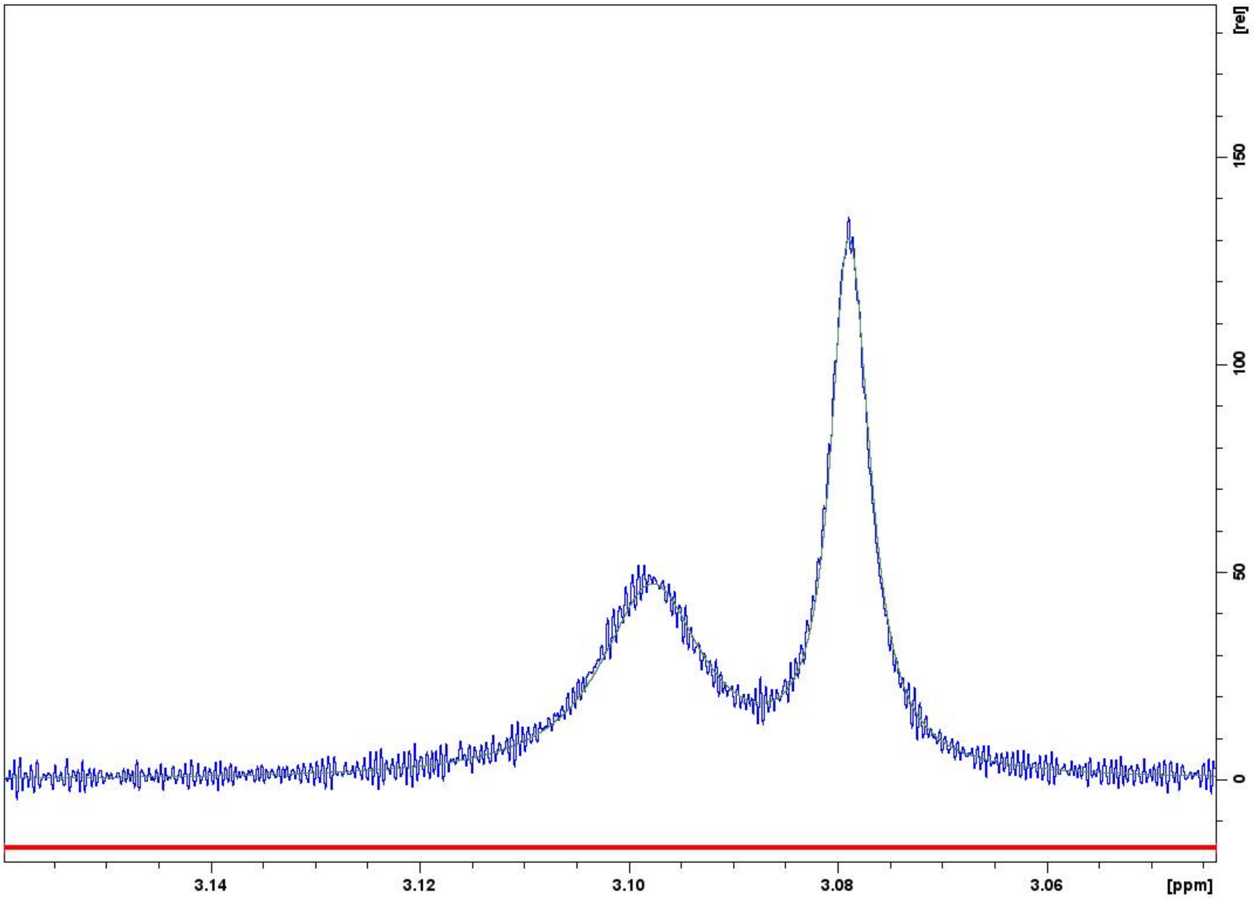

Figure S29. The experimental and the simulated spectra after the iterative parameter refinement of RD spectrum at 383 K of compound **1** in TCE-d2 using dnmr module v.1.1.2 of topspin program.

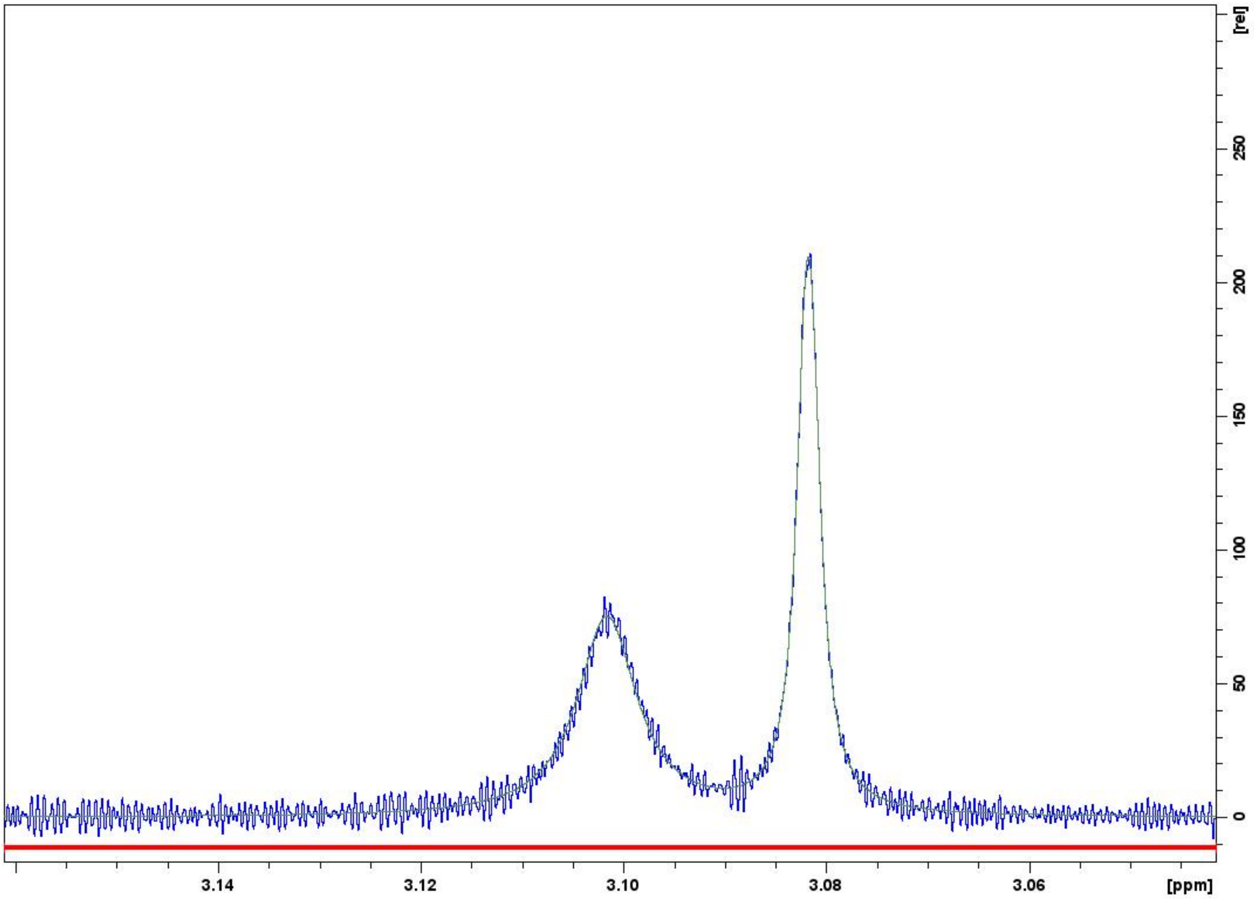

Figure S30. The experimental and the simulated spectra after the iterative parameter refinement of RD spectrum at 393 K of compound **1** in TCE-d2 using dnmr module v.1.1.2 of topspin program.

Table S6. Rate constants of compound **1** calculated from RD spectra in TCE-d2

T, K	*Amide (1* to *4)*,s^{-1}	*Enamine (2* to *3)*,s^{-1}
293	3.40706	
303	8.87147	
313	19.3724	
323	41.2255	
333	85.5686	2.55935
343	160.829	8.71379
353	243.14	19.6218
363	449.878	40.4488
373	971.28	94.1063
383	1617.52	175.018
393	2926.95	479.003

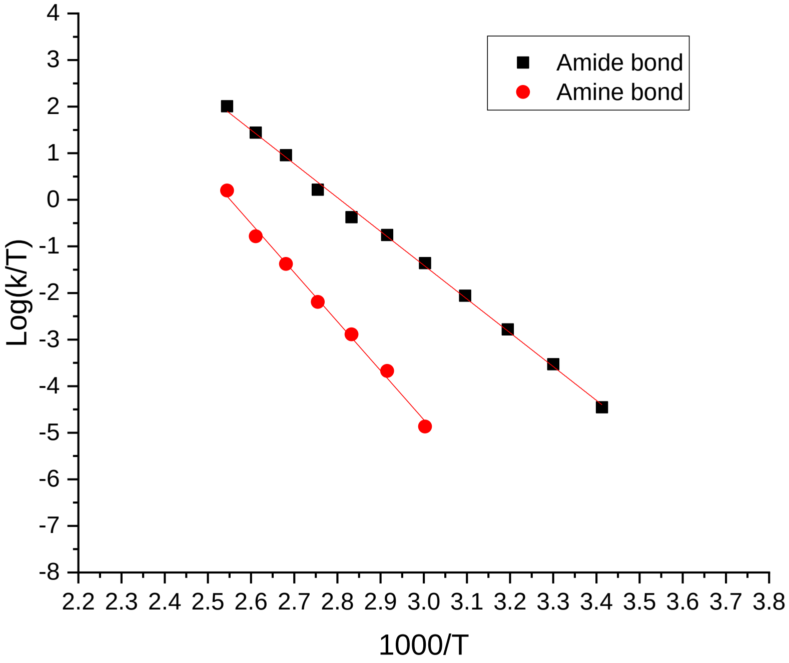

Figure S31. Eyring plot of rate constants of restricted rotation around amide and enamine bond in TCE-d2 using CLSA rate constants

Table S7. Experimental Activation Parameters for the Exchange Processes of complex **1** in TCE-d2 using CLSA rate constants

Exchange	$\Delta H^{\neq}$(298K)	$\Delta S^{\neq}$(298K)	$\Delta G^{\neq}$(298K)	R^2
Amide bond	14.4 ± 0.4	-6.7 ± 1.4	16.42 ± 0.08	0.9990
Enamine bond	20.9 ± 0.8	6.2 ± 2.0	19.07 ± 0.09	0.9976

$\Delta G^{\neq}$ and $\Delta H^{\neq}$ in kcal mol^{-1} and $\Delta S^{\neq}$ in cal mol^{-1} K^{-1}

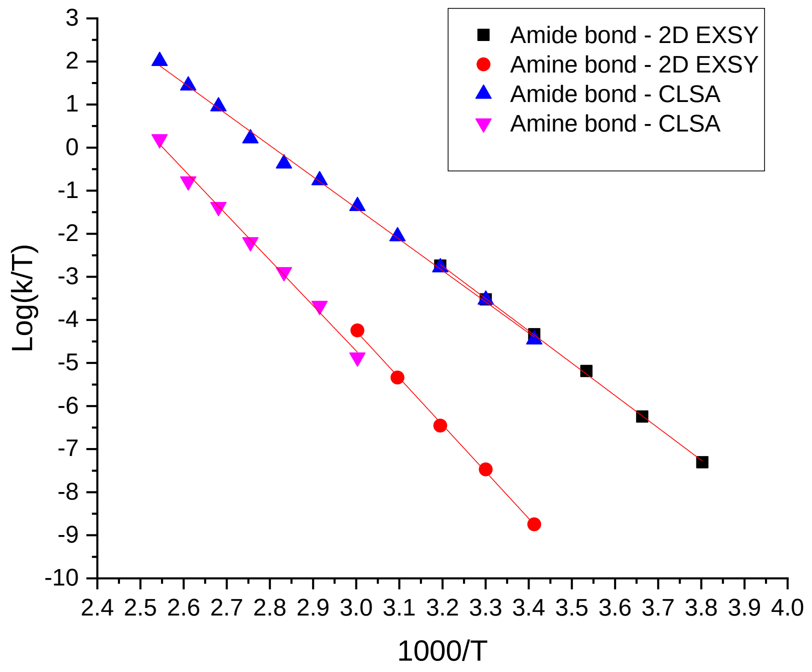

Figure S32. Eyring plot of rate constants of restricted rotation around amide and enamine bond in TCE-d2 using 2D EXSY and CLSA rate constants

Table S8. Experimental Activation Parameters for the Exchange Processes of complex **1** in TCE-d2 using 2D EXSY and CLSA rate constants

Exchange	$\Delta H^{\neq}$(298K)	$\Delta S^{\neq}$(298K)	$\Delta G^{\neq}$(298K)	R^2
Amide bond	14.4 ± 0.3	-6.7 ± 1.0	16.41 ± 0.08	0.9995
Enamine bond	19.8 ± 0.5	3.2 ± 1.2	18.84 ± 0.11	0.9982

$\Delta G^{\neq}$ and $\Delta H^{\neq}$ in kcal mol^{-1} and $\Delta S^{\neq}$ in cal mol^{-1} K^{-1}

III. DFT calculations

All calculations were performed by means of quantum chemical calculations at the density functional theory (DFT) level using Gaussian16 Rev. C.01 software package [1] with tight optimization criteria.

The geometries of all compounds have been fully optimized and the corresponding transition states were localized using B3LYP [2] or M06-2X [3,4] functional with 6-311++G(d,p) basis set [5]. Solvent effect was included implicitly to the optimizations via the SMD [6] model with the built in parameters for solvents $CHCl_3$ and acetone. As TCE is not by default parametrized within the SMD model implemented in Gaussian, the following parameters were used: eps = 8.42 (https://www.stenutz.eu/chem/dielectric_ri.php); epsinf = 2.028346 ($n^{D}{}_{20}$ = 1.495, epsinf = n^2 = 2.235025); HBondAcidity = 0.16 [7]; HBondBasicity = 0.12 [7]; SurfaceTensionAtInterface = 52.031418 (https://www.stenutz.eu/chem/dielectric_ri.php); ElectronegativeHalogenicity = 0.667; CarbonAromaticity = 0.00. The nature of all critical points was confirmed by means of the vibrational analysis.

The ΔH, ΔS and ΔG values were calculated at T = 298.15 K at the same level of theory including zero-point energy in the particular solvent environment (represented by relative permittivity) and vibrational, rotational and translational thermal energy corrections.

Literature:

[1] M.J. Frisch, G.W. Trucks, H.B. Schlegel, G.E. Scuseria, M.A. Robb, J.R. Cheeseman, G. Scalmani, V. Barone, G.A. Petersson, H. Nakatsuji, X. Li, M. Caricato, A.V. Marenich, J. Bloino, B.G. Janesko, R. Gomperts, B. Mennucci, H.P. Hratchian, J.V. Ortiz, A.F. Izmaylov, J.L. Sonnenberg, D. Williams-Young, F. Ding, F. Lipparini, F. Egidi, J. Goings, B. Peng, A. Petrone, T. Henderson, D. Ranasinghe, V.G. Zakrzewski, J. Gao, N. Rega, G. Zheng, W. Liang, M. Hada, M. Ehara, K. Toyota, R. Fukuda, J. Hasegawa, M. Ishida, T. Nakajima, Y. Honda, O. Kitao, H. Nakai, T. Vreven, K. Throssell, J.A. Montgomery, Jr., J.E. Peralta, F. Ogliaro, M.J. Bearpark, J.J. Heyd, E.N. Brothers, K.N. Kudin, V.N. Staroverov, T.A. Keith, R. Kobayashi, J. Normand, K. Raghavachari, A.P. Rendell, J.C. Burant, S.S. Iyengar, J. Tomasi, M. Cossi, J.M. Millam, M. Klene, C. Adamo, R. Cammi, J.W. Ochterski, R.L. Martin, K. Morokuma, O. Farkas, J.B. Foresman, D.J. Fox, Gaussian 16, Revision C.01, (2019).
[2] A.D. Becke, The Journal of Chemical Physics, 98 (1993) 5648-5652.
[3] Y. Zhao, D.G. Truhlar, Accounts of Chemical Research, 41 (2008) 157-167.
[4] Y. Zhao, D.G. Truhlar, Theoretical Chemistry Accounts, 120 (2008) 215-241.
[5] P.C. Hariharan, J.A. Pople, Theoretica Chimica Acta, 28 (1973) 213-222.
[6] A.V. Marenich, C.J. Cramer, D.G. Truhlar, Journal of Physical Chemistry B, 113 (2009) 6378-6396.
[7] M.H. Abraham, J. Andonian-Haftvan, G.S. Whiting, A. Leo, R.S. Taft, Journal of the Chemical Society, Perkin Transactions 2, (1994) 1777-1791.

Cartesian coordinates of the optimized GS geometries of **1**:

SMD($CDCl_3$)/B3LYP/6-311++G(d,p):

C	-1.07208600	-0.53762100	-0.03953100
O	-1.12818800	-1.77676800	-0.06132800
N	-2.20897100	0.22642600	-0.02855200
C	-2.22863900	1.67711700	0.09630900
H	-2.86311400	2.10775500	-0.68589500
H	-2.63863300	1.97511300	1.06966600
H	-1.22328100	2.07298800	-0.00017800
C	-3.51256300	-0.41769000	0.01320900
H	-4.17534700	0.03853400	-0.72905600
H	-3.40479700	-1.47711100	-0.20345900
H	-3.97552900	-0.30267100	1.00165000
N	0.11329500	0.20934400	-0.03703000
C	1.20665600	-0.49538600	-0.00345100
H	1.18107500	-1.58414000	0.05309900
N	2.43196600	0.03971400	-0.04166900
C	2.63795400	1.48200700	-0.09642200
H	3.54274800	1.69208600	-0.67034500
H	1.78474300	1.95645500	-0.57654700
H	2.75514500	1.89970800	0.91017200
C	3.61948000	-0.78895600	0.12482500
H	3.33733900	-1.84125400	0.15057700
H	4.31159300	-0.62856800	-0.70681800
H	4.13472700	-0.53997600	1.05887900

SMD($CDCl_3$)/M062X/6-311++G(d,p):

C	-1.06218600	-0.53569100	0.03636100
O	-1.12164000	-1.76664500	0.00331000
N	-2.19175900	0.22649500	0.09506300
C	-2.20819400	1.66919200	-0.07372600
H	-2.51163700	1.93718500	-1.09282800
H	-2.92795400	2.10351900	0.62444700
H	-1.22378700	2.08003200	0.12401300
C	-3.48306500	-0.42740600	-0.00907700
H	-3.82036700	-0.47799200	-1.05145900
H	-3.41964700	-1.43732600	0.38757100
H	-4.21682100	0.14152600	0.56520800
N	0.12243000	0.20899300	0.04349500
C	1.20425200	-0.50562800	-0.02947200
H	1.17545300	-1.59441400	-0.08532500
N	2.41747600	0.04010400	-0.04727200
C	2.57094000	1.48320800	0.05109300
H	3.59420000	1.74437300	-0.21624500
H	1.87796200	1.97763700	-0.62961300

H	2.36397900	1.82799500	1.06854700
C	3.62078500	-0.77445000	-0.02526800
H	3.35123400	-1.82773600	-0.09262300
H	4.26399600	-0.51719300	-0.86980400
H	4.17429500	-0.60892900	0.90316300

SMD(TCE)/B3LYP/6-311++G(d,p):

C	-1.07283500	-0.53366100	-0.05161700
O	-1.13353500	-1.77451600	-0.07179500
N	-2.20907100	0.23051400	-0.04886500
C	-2.22658200	1.67682300	0.12887300
H	-2.90792700	2.12707100	-0.59984200
H	-2.57908800	1.94095200	1.13422900
H	-1.23061500	2.08277500	-0.01354600
C	-3.51032000	-0.41891100	0.01263500
H	-4.21679300	0.12382600	-0.62137300
H	-3.42897300	-1.44519400	-0.33625300
H	-3.90416700	-0.42549400	1.03747100
N	0.11277900	0.20902700	-0.05080900
C	1.20730300	-0.49616500	0.00353100
H	1.18201700	-1.58319900	0.08350200
N	2.43115700	0.03724700	-0.03866500
C	2.64036500	1.47800100	-0.12533400
H	3.55326400	1.67151100	-0.69170800
H	1.79543000	1.94374000	-0.62816400
H	2.74609100	1.91807100	0.87275100
C	3.61833800	-0.78836900	0.15016000
H	3.33509200	-1.83920100	0.20338600
H	4.31052700	-0.64882700	-0.68485100
H	4.13174900	-0.51372100	1.07764600

SMD(TCE)/M062X/6-311++G(d,p):

C	-1.06350500	-0.53459600	0.06183800
O	-1.12767100	-1.76750800	0.05561200
N	-2.19151900	0.23125300	0.09029000
C	-2.19692200	1.66862200	-0.12647300
H	-2.42761200	1.90476000	-1.17213200
H	-2.96498800	2.11987600	0.50557400
H	-1.23014300	2.09048300	0.12777500
C	-3.48221900	-0.42191000	-0.03360800
H	-3.78160300	-0.51579300	-1.08454200
H	-3.44063000	-1.41402800	0.40850400
H	-4.23242900	0.17464300	0.48824400
N	0.12159000	0.20512600	0.06429000
C	1.20547000	-0.51134700	0.00250200
H	1.17803100	-1.59878400	-0.07308700
N	2.41677800	0.03334400	0.02876500

C	2.56673900	1.47873000	0.10740700
H	3.60953700	1.70980300	0.32000400
H	2.27751800	1.95025400	-0.83621600
H	1.93580100	1.87748300	0.90211500
C	3.61880200	-0.76749600	-0.13945800
H	3.35077000	-1.82161800	-0.19590400
H	4.13710400	-0.48238200	-1.05879200
H	4.29187700	-0.61472100	0.70690000

Cartesian coordinates of the optimized GS geometries of **2**:

SMD($CDCl_3$)/B3LYP/6-311++G(d,p):

C	-1.14367400	-0.52533400	0.00304600
O	-1.18472500	-1.76937000	-0.04857000
N	-2.30723500	0.20872400	0.08353400
C	-2.34577300	1.66780500	0.08097800
H	-2.11162700	2.09091000	-0.90383300
H	-3.35350700	1.98538300	0.35164300
H	-1.66185400	2.08972300	0.81924200
C	-3.59269000	-0.46440300	-0.04899000
H	-4.07521900	-0.20601700	-1.00022600
H	-3.43757200	-1.53947800	-0.01413800
H	-4.26053900	-0.17100900	0.76708100
C	1.29387000	-0.47891700	-0.03475000
H	1.23908600	-1.56237900	-0.00922300
N	2.54614100	0.02225300	-0.10845800
C	2.77368600	1.45859200	-0.08888200
H	3.81369000	1.66026700	-0.34668700
H	2.13466600	1.95516500	-0.82332000
H	2.56835300	1.88978800	0.90016800
C	3.69899400	-0.83537300	0.13750400
H	3.41362900	-1.87995700	0.01126200
H	4.49775300	-0.60402700	-0.57224300
H	4.08926500	-0.70126900	1.15461300
C	0.12057600	0.21684100	-0.00915400
H	0.11940000	1.29574800	-0.00983200

SMD($CDCl_3$)/M062X/6-311++G(d,p):

C	-1.14018000	-0.52278000	-0.06352100
O	-1.17502400	-1.75960200	-0.07396600
N	-2.29559400	0.21192800	-0.05313900
C	-2.31179900	1.66063900	0.08692000
H	-1.74943000	2.14738200	-0.71189100
H	-3.34554000	1.99741400	0.01631400
H	-1.91371500	1.98526700	1.05379300
C	-3.57417500	-0.46596700	0.07562500
H	-4.29113400	-0.04077900	-0.63056400

H	-3.44241600	-1.52262100	-0.13953100
H	-3.97577200	-0.35724400	1.08962900
C	1.28438000	-0.48319400	-0.06532900
H	1.22044100	-1.56720600	-0.04393000
N	2.53337400	0.01619300	-0.10518800
C	2.73664800	1.45030100	-0.05995300
H	3.79235300	1.66624100	-0.22021700
H	2.15786400	1.93832900	-0.84780800
H	2.43179600	1.86703700	0.90815200
C	3.68042000	-0.82879300	0.17997500
H	3.40336400	-1.87446700	0.04994400
H	4.49612200	-0.59393000	-0.50700900
H	4.03413900	-0.68314500	1.20692900
C	0.12303800	0.22536900	-0.06531200
H	0.12767000	1.30424100	-0.05422500

SMD(Ace)/B3LYP/6-311++G(d,p):

C	-1.14423800	-0.52500700	0.01908000
O	-1.18939000	-1.77014500	-0.03615100
N	-2.30800500	0.20998900	0.10451900
C	-2.34788000	1.66875700	0.03574500
H	-2.13976800	2.04420300	-0.97402800
H	-3.34816200	1.99836500	0.31914300
H	-1.64352100	2.12322700	0.73328200
C	-3.59053100	-0.46617400	-0.04794500
H	-4.00028600	-0.32098800	-1.05659300
H	-3.45973300	-1.53119100	0.12523300
H	-4.30793500	-0.06707500	0.67424900
C	1.29649500	-0.48111600	-0.03205200
H	1.24652000	-1.56521300	-0.02834000
N	2.54443200	0.02465600	-0.09834400
C	2.76994000	1.46193300	-0.05541500
H	3.80889800	1.66817600	-0.31266000
H	2.12673200	1.97067900	-0.77780000
H	2.56700500	1.87313600	0.94208700
C	3.70618800	-0.83626700	0.09339500
H	3.41654400	-1.87915900	-0.03662300
H	4.47916400	-0.59203200	-0.64009300
H	4.13015600	-0.71299200	1.09774200
C	0.12053600	0.21401400	0.01200000
H	0.12145800	1.29267100	0.03153600

SMD(Ace)/M062X/6-311++G(d,p):

C	-1.14065700	-0.52662100	0.01872600
O	-1.17887600	-1.76405800	-0.02903800
N	-2.29426600	0.20760300	0.09374200
C	-2.31287600	1.66170700	0.00640900

H	-1.95263300	2.01944600	-0.96357000
H	-3.34221500	1.99600400	0.13107900
H	-1.71607000	2.11847200	0.79769700
C	-3.57802200	-0.46303300	-0.02613900
H	-3.99413200	-0.34042500	-1.03301600
H	-3.45067500	-1.52295200	0.17577200
H	-4.28191300	-0.03990500	0.69365400
C	1.28711100	-0.48575600	-0.03532200
H	1.23032200	-1.57042400	-0.04357300
N	2.52844900	0.02107200	-0.08771200
C	2.72798300	1.45608400	-0.02497700
H	3.78301300	1.67591900	-0.18165500
H	2.14594800	1.95485800	-0.80435600
H	2.42168000	1.85609400	0.94896500
C	3.69827500	-0.82772100	0.06787900
H	3.40762000	-1.87187000	-0.04256300
H	4.44066400	-0.58430000	-0.69539800
H	4.15243600	-0.68906200	1.05468900
C	0.12169000	0.22044300	0.01219000
H	0.12666000	1.29926600	0.03976900

Cartesian coordinates of the optimized anti-TS geometry of **1** regarding rotation around **amide** bond:

SMD($CDCl_3$)/B3LYP/6-311++G(d,p):

O	1.17216400	1.58564000	-0.00026800
C	1.02889300	0.36669600	-0.00007000
N	2.17886600	-0.52186700	0.00008900
C	2.98532500	-0.32681600	1.21141500
C	2.98536300	-0.32720600	-1.21127400
N	-0.16526600	-0.32039000	0.00001600
C	-1.24772000	0.42490500	-0.00000400
N	-2.47729700	-0.07454900	0.00001500
C	-3.64847700	0.79629900	0.00001900
C	-2.74133600	-1.51082300	0.00003600
H	2.36589900	-0.48297300	2.09820100
H	3.78500400	-1.07166800	1.22292300
H	3.43872200	0.67461000	1.27094900
H	2.36596600	-0.48365800	-2.09802900
H	3.78504700	-1.07205700	-1.22251200
H	3.43875400	0.67420400	-1.27112100
H	-1.19224900	1.51423100	-0.00002700
H	-3.33838400	1.84062200	-0.00000700
H	-4.25675700	0.60375300	0.88842500
H	-4.25678900	0.60371800	-0.88835600
H	-3.31997200	-1.77759400	0.88896000

H	-3.31996400	-1.77762200	-0.88888600
H	-1.79900300	-2.05136900	0.00004800

SMD($CDCl_3$)/M062X/6-311++G(d,p):

O	-1.16825700	1.55340400	0.03440900
C	-1.02398000	0.34255300	0.00719500
N	-2.17000700	-0.53919900	-0.01400200
C	-2.97378200	-0.33277100	1.19218000
C	-2.97124100	-0.27959300	-1.21151100
N	0.16976000	-0.34271700	-0.00816800
C	1.23714000	0.41914000	0.01314300
N	2.46612500	-0.06571000	0.02305700
C	3.63061600	0.80553700	-0.01509800
C	2.73404800	-1.49653500	-0.00766000
H	-2.36034100	-0.50873300	2.07841200
H	-3.79168600	-1.05587800	1.19332200
H	-3.39551400	0.68051600	1.24646500
H	-2.35566100	-0.41496200	-2.10334200
H	-3.78841900	-1.00265300	-1.24701400
H	-3.39395200	0.73471100	-1.22120200
H	1.16588600	1.50775600	0.02659400
H	3.31548900	1.84773700	-0.02182200
H	4.25728700	0.62285700	0.86058200
H	4.21440000	0.60041600	-0.91591800
H	1.83805200	-2.04059600	0.27776400
H	3.54417900	-1.71749500	0.68914200
H	3.03838700	-1.79751000	-1.01397400

SMD(TCE)/B3LYP/6-311++G(d,p):

O	-1.17353400	1.58524400	0.00036400
C	-1.02806500	0.36524300	0.00009400
N	-2.17914500	-0.52326000	-0.00012100
C	-2.98664300	-0.32699800	1.21104400
C	-2.98659300	-0.32646900	-1.21123400
N	0.16393000	-0.32094900	-0.00002300
C	1.24943200	0.42485400	0.00000200
N	2.47664800	-0.07415600	-0.00002300
C	3.64859800	0.79718100	-0.00002400
C	2.74478100	-1.50997300	-0.00004800
H	-2.36791100	-0.48093400	2.09866000
H	-3.78648700	-1.07153700	1.22259400
H	-3.44084700	0.67406700	1.26813000
H	-2.36782100	-0.48000600	-2.09889100
H	-3.78642900	-1.07101000	-1.22314900
H	-3.44080400	0.67461600	-1.26789700
H	1.19439000	1.51377900	0.00003100
H	3.33856800	1.84136100	0.00001300

H	4.25562500	0.60237600	0.88839600
H	4.25558800	0.60242600	-0.88847900
H	1.80546100	-2.05541500	-0.00010900
H	3.32488000	-1.77309700	0.88871600
H	3.32496800	-1.77304500	-0.88876800

SMD(TCE)/M062X/6-311++G(d,p):

O	-1.16818300	1.55455800	-0.07066900
C	-1.02041900	0.34393400	-0.01661800
N	-2.16607300	-0.53850900	0.02755400
C	-2.96522200	-0.25230200	1.22104500
C	-2.97453700	-0.35923500	-1.18050800
N	0.17260500	-0.33839000	0.01034500
C	1.24203600	0.42551300	-0.02002300
N	2.46565700	-0.06526000	-0.02945500
C	3.63624200	0.79896200	0.02329000
C	2.71395600	-1.50054100	0.01360200
H	-2.34728800	-0.36358600	2.11458400
H	-3.78071400	-0.97597700	1.27562400
H	-3.39122300	0.76045700	1.20667300
H	-2.36413600	-0.55251500	-2.06523800
H	-3.79172400	-1.08283000	-1.16280500
H	-3.39845400	0.65179300	-1.25416500
H	1.17277200	1.51369600	-0.04045000
H	3.32656700	1.84272300	0.02344500
H	4.20451300	0.59107700	0.93312200
H	4.27285900	0.60877700	-0.84299800
H	1.89266200	-2.02985100	-0.46294600
H	2.80637100	-1.83669500	1.05006100
H	3.64558800	-1.70640700	-0.51338200

<u>Cartesian coordinates of the optimized syn-TS geometry of **1** regarding rotation around **amide** bond:</u>

SMD($CDCl_3$)/B3LYP/6-311++G(d,p):

O	-1.09839800	1.98072500	0.18366500
C	-1.11731000	0.76215600	0.07811100
N	-2.38824600	0.07152300	-0.01031300
C	-2.60177200	-0.84378200	1.11602900
C	-2.55119000	-0.61267500	-1.29775000
N	-0.01671600	-0.07500300	0.03876200
C	1.15356700	0.51377400	-0.00465600
N	2.30975100	-0.14131400	-0.00608800
C	3.57878500	0.57283900	-0.09744700
C	2.38942100	-1.59679200	0.07051200
H	-2.47122400	-0.30900100	2.05979300

H	-1.92301700	-1.71017300	1.10510000
H	-3.63172700	-1.20862700	1.07671400
H	-2.39334900	0.09332300	-2.11666200
H	-1.86289600	-1.46229600	-1.42667800
H	-3.57727000	-0.98352800	-1.36779900
H	1.24434100	1.59944900	-0.05015000
H	3.39731300	1.64523400	-0.16020200
H	4.19193600	0.36386400	0.78393800
H	4.12618500	0.25128000	-0.98830200
H	1.38786300	-2.01036000	0.14922800
H	2.97718400	-1.88636300	0.94623500
H	2.87931800	-1.98816000	-0.82585600

SMD($CDCl_3$)/M062X/6-311++G(d,p):

O	-1.08867500	1.98385700	0.09995500
C	-1.11644000	0.77073100	0.04360800
N	-2.38285200	0.08350700	-0.01174500
C	-2.55673900	-0.79636100	1.14429100
C	-2.51573900	-0.67215200	-1.25783200
N	-0.02054500	-0.07546200	0.02455800
C	1.14243500	0.52200700	0.01941000
N	2.28991700	-0.13629600	0.03382400
C	3.56379300	0.55599400	-0.08194800
C	2.33403400	-1.59129800	0.04505400
H	-2.41813900	-0.22856300	2.06644500
H	-1.85732900	-1.64373100	1.13841300
H	-3.57831500	-1.18241700	1.13307900
H	-2.35428500	-0.01051700	-2.11121800
H	-1.80954800	-1.51183000	-1.31834700
H	-3.53406500	-1.06219000	-1.31795800
H	1.23116100	1.60903000	0.00302400
H	3.39453500	1.63034900	-0.13410000
H	4.19089200	0.32932400	0.78298400
H	4.08082400	0.23106300	-0.98850300
H	1.43940300	-1.98010500	0.52523800
H	3.21987700	-1.90898400	0.59595500
H	2.39068900	-1.97806200	-0.97661700

SMD(TCE)/B3LYP/6-311++G(d,p):

O	-1.09988700	1.98311300	0.17310700
C	-1.11570700	0.76204800	0.07285200
N	-2.38870300	0.07235900	-0.00923800
C	-2.59972400	-0.83805200	1.12253500
C	-2.55395900	-0.62131900	-1.29219500
N	-0.01799800	-0.07301300	0.03444800
C	1.15579300	0.51514700	-0.00552200
N	2.30860300	-0.14106800	-0.00561500

C	3.57976000	0.57164600	-0.09118500
C	2.38926300	-1.59710100	0.06668900
H	-2.46339700	-0.30018200	2.06372000
H	-1.92322100	-1.70591100	1.11149200
H	-3.63025100	-1.20154100	1.08861800
H	-2.39413900	0.07765000	-2.11670800
H	-1.86792400	-1.47342400	-1.41420900
H	-3.58059100	-0.99104800	-1.35860600
H	1.24871300	1.60031400	-0.04929000
H	3.40010800	1.64430300	-0.15148100
H	4.18839600	0.35724400	0.79168400
H	4.12786200	0.24965700	-0.98110400
H	2.87966200	-1.98370000	-0.83119800
H	1.38906400	-2.01363400	0.14524800
H	2.97893500	-1.88679100	0.94076800

SMD(TCE)/M062X/6-311++G(d,p):

O	-1.09040600	1.98430600	-0.12213200
C	-1.11420600	0.76992400	-0.05181100
N	-2.38191100	0.08390900	0.01205100
C	-2.51395000	-0.65925700	1.26637900
C	-2.55738200	-0.80967600	-1.13400600
N	-0.02059200	-0.07298800	-0.02579800
C	1.14546000	0.52474100	-0.01578500
N	2.28886400	-0.13558800	-0.03135300
C	3.56523300	0.55267400	0.09115300
C	2.32924600	-1.59119400	-0.05386800
H	-2.34846800	0.00986400	2.11316100
H	-1.80989600	-1.50003900	1.33294400
H	-3.53267000	-1.04716500	1.33227700
H	-2.41539300	-0.25419900	-2.06314500
H	-1.86090200	-1.65903900	-1.11624200
H	-3.57978900	-1.19323800	-1.11879700
H	1.23634200	1.61112300	0.00788100
H	3.39839900	1.62694500	0.14922000
H	4.07797000	0.21916300	0.99671600
H	4.19207900	0.32744600	-0.77400700
H	1.45410800	-1.97465300	-0.57298900
H	2.34518100	-1.98643900	0.96582200
H	3.23535800	-1.90481800	-0.57245500

<u>Cartesian coordinates of the optimized anti-TS geometry of **1** regarding rotation around **enamine** bond:</u>

SMD($CDCl_3$)/B3LYP/6-311++G(d,p):

C	1.11607100	-0.57934800	-0.04403500

O	1.15676600	-1.76079700	0.29896200
N	2.20625300	0.22752700	-0.10950300
C	2.15281900	1.65105400	-0.42798000
H	2.22584800	2.25885500	0.48169600
H	2.99396500	1.90318900	-1.07922300
H	1.22377500	1.88845100	-0.93894000
C	3.50155500	-0.27669500	0.32896100
H	3.79614700	0.18513400	1.27837100
H	3.44932700	-1.35441200	0.45905000
H	4.26018800	-0.03829200	-0.42194300
N	-0.08776200	0.04712700	-0.48575900
C	-1.14610900	-0.22744200	0.15317200
H	-1.13737400	-0.87517900	1.04417900
N	-2.40887100	0.28443700	-0.25151100
C	-3.32379500	-0.82661100	-0.56038200
H	-2.90629700	-1.43821600	-1.36222100
H	-4.27239200	-0.40961100	-0.90471900
H	-3.52269400	-1.47229800	0.31097400
C	-2.96482200	1.15273400	0.79815900
H	-2.28996500	1.99079300	0.98247400
H	-3.13675600	0.61991500	1.74836700
H	-3.91955900	1.55226100	0.45028000

SMD($CDCl_3$)/M062X/6-311++G(d,p):

C	1.10142800	-0.56084300	-0.05259700
O	1.11208300	-1.72596900	0.31987000
N	2.20715100	0.21183300	-0.13754000
C	2.18427400	1.64106500	-0.41155900
H	2.26528200	2.21397000	0.51829300
H	3.03455000	1.89099400	-1.04915300
H	1.26458000	1.91319700	-0.92081500
C	3.47257700	-0.32159900	0.33854100
H	3.66521100	-0.00870800	1.37041000
H	3.45306500	-1.40733600	0.29445300
H	4.27637200	0.05403800	-0.29691300
N	-0.08674800	0.09083200	-0.50316300
C	-1.14252700	-0.21703600	0.11778400
H	-1.13587300	-0.91129800	0.97296100
N	-2.40260500	0.31232600	-0.25924200
C	-3.30442600	-0.79611900	-0.59142000
H	-2.89424400	-1.36946000	-1.42359800
H	-4.26583700	-0.38019600	-0.89615800
H	-3.46817400	-1.47200500	0.26217800
C	-2.94910700	1.09727800	0.85230400
H	-2.28232400	1.93007300	1.07988600
H	-3.09085000	0.49240600	1.76157800
H	-3.91632500	1.50066700	0.54920700

SMD(TCE)/B3LYP/6-311++G(d,p):

C	1.12225700	-0.59505000	-0.06392100
O	1.18108800	-1.77843300	0.27509500
N	2.19637800	0.23125700	-0.11594300
C	2.11034500	1.66032400	-0.40325500
H	2.14155000	2.24867800	0.52126700
H	2.96039900	1.94994600	-1.02593800
H	1.18890900	1.88433100	-0.93425800
C	3.49507300	-0.24845300	0.34165700
H	3.73267500	0.15427800	1.33310800
H	3.48872900	-1.33416600	0.39227400
H	4.26809800	0.07759800	-0.35893300
N	-0.08942700	-0.00059200	-0.52270800
C	-1.13603500	-0.21823800	0.15621000
H	-1.11371700	-0.78925900	1.09758900
N	-2.40519600	0.26325000	-0.26517200
C	-3.32700000	-0.86858800	-0.46328400
H	-2.92753500	-1.54364000	-1.22232100
H	-4.28333300	-0.47886500	-0.81758500
H	-3.50457500	-1.44031300	0.46214900
C	-2.94050800	1.21125200	0.72623100
H	-2.26371900	2.06139300	0.83021400
H	-3.08935500	0.75204200	1.71731100
H	-3.90392000	1.58055200	0.36928400

SMD(TCE)/M062X/6-311++G(d,p):

C	1.10715500	-0.57363600	-0.07075400
O	1.13633800	-1.73839500	0.30639100
N	2.19849000	0.21747400	-0.14572900
C	2.14535200	1.65115900	-0.39427200
H	2.20083000	2.20845700	0.54668800
H	2.99608700	1.93262100	-1.01724800
H	1.22529000	1.91107100	-0.90982600
C	3.46379400	-0.29875300	0.35184900
H	3.58132000	-0.09115900	1.42083400
H	3.51299800	-1.37315400	0.19219200
H	4.27588000	0.18589700	-0.19106400
N	-0.08877300	0.04426800	-0.54195200
C	-1.13276000	-0.20869800	0.12243300
H	-1.11250600	-0.82921100	1.03202200
N	-2.39872800	0.29251300	-0.27234900
C	-3.30450700	-0.83845600	-0.50797500
H	-2.90854400	-1.46963400	-1.30449400
H	-4.27244900	-0.44597700	-0.82267600
H	-3.44945500	-1.45012200	0.39551800
C	-2.92912000	1.15269900	0.79168100

H	-2.25956600	1.99886300	0.95173700
H	-3.05546300	0.61032600	1.74122300
H	-3.90152800	1.53349800	0.47641000

Cartesian coordinates of the optimized syn-TS geometry of **1** regarding rotation around **enamine** bond:

SMD($CDCl_3$)/B3LYP/6-311++G(d,p):

C	1.08420500	-0.61813800	-0.00580500
O	1.28424900	-1.80899700	0.23121300
N	2.07432800	0.29963800	-0.15347600
C	1.84365600	1.72994400	-0.32907600
H	1.97989000	2.26893700	0.61605400
H	2.56194200	2.12013000	-1.05487700
H	0.83644400	1.90698400	-0.69557800
C	3.45893800	-0.09711000	0.07138000
H	3.82290800	0.28528500	1.03221300
H	3.53389400	-1.18137300	0.07037400
H	4.08776400	0.31125600	-0.72440800
N	-0.22618000	-0.09603200	-0.22096400
C	-1.14980900	-0.52678700	0.53442900
H	-0.94835200	-1.22277100	1.35392900
N	-2.51804100	-0.14942000	0.36742100
C	-3.06698100	-0.63055600	-0.90940300
H	-2.90982800	-1.70773700	-0.99822100
H	-2.61413500	-0.13689700	-1.78193600
H	-4.14306400	-0.44192000	-0.91726400
C	-2.73375200	1.29233000	0.55047200
H	-2.32960700	1.60894200	1.51467900
H	-3.80927400	1.48442000	0.55412200
H	-2.27087900	1.89931700	-0.24164400

SMD($CDCl_3$)/M062X/6-311++G(d,p):

C	1.07182800	-0.62064000	-0.02209100
O	1.27526600	-1.79817800	0.23698200
N	2.05135800	0.29734700	-0.18424200
C	1.80825700	1.72701200	-0.30590600
H	1.91196500	2.22368800	0.66477900
H	2.54374600	2.14983700	-0.99280800
H	0.81081700	1.90869500	-0.69593300
C	3.42780600	-0.09201800	0.07347400
H	3.72573300	0.17660600	1.09273700
H	3.53569600	-1.16557900	-0.05684100
H	4.08010000	0.42725000	-0.63073600
N	-0.23946200	-0.10636000	-0.24240400
C	-1.13966400	-0.54380900	0.53085900

H	-0.91767800	-1.24119600	1.34300600
N	-2.50613500	-0.16137900	0.38680900
C	-3.03912900	-0.60463100	-0.90572100
H	-2.89790400	-1.68116300	-1.01474800
H	-2.55578200	-0.09602000	-1.75072000
H	-4.10967400	-0.39347600	-0.92759800
C	-2.67531600	1.28488600	0.55274900
H	-2.26762600	1.59810600	1.51551800
H	-3.74297800	1.51094500	0.54173700
H	-2.18156400	1.85566600	-0.24557200

SMD(TCE)/B3LYP/6-311++G(d,p):

C	1.08564800	-0.62630600	-0.01828600
O	1.29158000	-1.81926200	0.21156700
N	2.06912700	0.29722900	-0.15827400
C	1.82532600	1.72864100	-0.31014800
H	1.93927300	2.25048900	0.64721700
H	2.55159000	2.13918800	-1.01592500
H	0.82269800	1.90288300	-0.69109000
C	3.45618100	-0.08997300	0.07124300
H	3.80099900	0.25570200	1.05276400
H	3.54864700	-1.17192400	0.02428500
H	4.08846100	0.36215300	-0.69710700
N	-0.22757200	-0.11934400	-0.23960900
C	-1.13872900	-0.48890200	0.56152200
H	-0.92523500	-1.11967600	1.42932300
N	-2.50913900	-0.12131400	0.38977600
C	-3.07981000	-0.69467800	-0.83906800
H	-2.92518600	-1.77579400	-0.85063700
H	-2.64089500	-0.26720500	-1.75259000
H	-4.15540200	-0.50367400	-0.84373300
C	-2.71718100	1.33194700	0.46438000
H	-2.29448500	1.72057400	1.39360100
H	-3.79179600	1.52840200	0.47120900
H	-2.26683600	1.87261000	-0.38096100

SMD(TCE)/M062X/6-311++G(d,p):

C	1.07364200	-0.62825700	-0.03438200
O	1.28647700	-1.80616800	0.22241700
N	2.04503800	0.29694700	-0.19172300
C	1.78659400	1.72648500	-0.28900700
H	1.87784700	2.20574700	0.69144600
H	2.52137500	2.16968900	-0.96317800
H	0.78956700	1.90466200	-0.68248100
C	3.42297200	-0.08444200	0.07340100
H	3.69292300	0.11858700	1.11530200
H	3.55712300	-1.14418300	-0.12820100

H	4.07836100	0.49493500	-0.57830900
N	-0.24106000	-0.13104400	-0.26143600
C	-1.12865000	-0.51226400	0.55469200
H	-0.89488600	-1.15110300	1.41057600
N	-2.49642100	-0.13546600	0.40827300
C	-3.05266800	-0.66185800	-0.84304900
H	-2.91732600	-1.74396200	-0.88207200
H	-2.58161200	-0.21264500	-1.72768000
H	-4.12233200	-0.44629300	-0.86206200
C	-2.65554500	1.32053600	0.47706500
H	-2.22661400	1.69687100	1.40748400
H	-3.72204700	1.55186300	0.47045700
H	-2.17517000	1.83091400	-0.36874200

Cartesian coordinates of the optimized anti-TS geometry of **2** regarding rotation around **amide** bond:

SMD($CDCl_3$)/B3LYP/6-311++G(d,p):

O	-1.26070100	-1.60818300	0.00001200
C	-1.09927700	-0.38718400	0.00000400
N	-2.25410900	0.51599600	-0.00000500
C	-3.06218700	0.32316600	-1.21060900
C	-3.06218900	0.32318600	1.21060000
C	1.33980000	-0.42860000	0.00000700
N	2.58475000	0.05833300	0.00000900
C	3.74896600	-0.82168500	-0.00000800
C	2.82567600	1.49738700	-0.00000300
H	-2.43996800	0.46854500	-2.09753500
H	-3.85786500	1.07278400	-1.22783100
H	-3.52220800	-0.67520500	-1.26676100
H	-2.43997200	0.46857800	2.09752500
H	-3.85786800	1.07280300	1.22780800
H	-3.52221000	-0.67518500	1.26676700
H	1.26970300	-1.51210000	0.00001200
H	3.42399100	-1.86158600	0.00001000
H	4.36255300	-0.64452700	-0.88882300
H	4.36259100	-0.64450800	0.88877700
H	2.39032100	1.96469300	0.88880500
H	2.39038600	1.96467000	-0.88885600
H	3.89911600	1.67975600	0.00003500
C	0.16821700	0.29263500	0.00000000
H	0.15851600	1.37300200	-0.00000900

SMD($CDCl_3$)/M062X/6-311++G(d,p):

O	-1.25321200	-1.57522700	0.00000200
C	-1.09421400	-0.36271000	0.00000100

N	-2.24465400	0.53149000	-0.00000100
C	-3.04784400	0.30065500	-1.20205200
C	-3.04784600	0.30065800	1.20205000
C	1.32932800	-0.42430500	0.00000200
N	2.57353200	0.04920600	0.00000200
C	3.73344000	-0.82791400	-0.00000200
C	2.80780400	1.48331300	-0.00000000
H	-2.42950600	0.44647400	-2.09067500
H	-3.86172900	1.02837000	-1.22636500
H	-3.47613600	-0.71070600	-1.23148600
H	-2.42950900	0.44647800	2.09067300
H	-3.86173100	1.02837300	1.22636000
H	-3.47613800	-0.71070300	1.23148500
H	1.24180800	-1.50757200	0.00000200
H	3.40520300	-1.86618800	0.00000300
H	4.34383500	-0.64816600	-0.88880800
H	4.34384400	-0.64816000	0.88879600
H	2.36733900	1.94436900	0.88882100
H	2.36734300	1.94436600	-0.88882500
H	3.88043500	1.66816000	0.00000200
C	0.17497800	0.31765000	0.00000100
H	0.17461700	1.39777200	0.00000000

SMD(Ace)/B3LYP/6-311++G(d,p):

O	-1.26517800	-1.61593000	-0.00000400
C	-1.09844300	-0.39448400	-0.00000100
N	-2.25173300	0.51342500	0.00000200
C	-3.06174100	0.32919100	-1.21065500
C	-3.06174100	0.32918300	1.21065700
C	1.34473400	-0.43456400	-0.00000100
N	2.58377800	0.05899100	-0.00000100
C	3.75472000	-0.81397000	0.00000100
C	2.81814300	1.50014000	0.00000100
H	-2.43830500	0.46754200	-2.09796500
H	-3.85048300	1.08616000	-1.22728100
H	-3.53339800	-0.66398200	-1.26626700
H	-2.43830400	0.46752900	2.09796800
H	-3.85048200	1.08615300	1.22728900
H	-3.53339700	-0.66398900	1.26626400
H	1.28264900	-1.51854600	-0.00000300
H	3.43667600	-1.85595100	-0.00000200
H	4.36563400	-0.62874200	-0.88856800
H	4.36562900	-0.62874500	0.88857400
H	2.37988200	1.96385600	0.88901300
H	2.37987400	1.96385900	-0.88900600
H	3.89071900	1.68586000	-0.00000300
C	0.16767100	0.28370800	0.00000000

H	0.16035700	1.36429200	0.00000200

SMD(Ace)/M062X/6-311++G(d,p):

O	-1.25749700	-1.58021600	-0.00003000
C	-1.09345400	-0.36747000	-0.00000900
N	-2.24300600	0.53044300	0.00001100
C	-3.04807100	0.30474700	-1.20205400
C	-3.04806100	0.30470300	1.20207500
C	1.33394900	-0.42908700	-0.00000900
N	2.57275300	0.04945300	-0.00000900
C	3.73809200	-0.82257700	0.00001100
C	2.80297500	1.48529300	0.00000400
H	-2.42969300	0.44772700	-2.09131100
H	-3.85923200	1.03563800	-1.22480100
H	-3.48192300	-0.70434400	-1.23107600
H	-2.42967500	0.44764900	2.09133200
H	-3.85922100	1.03559400	1.22485700
H	-3.48191400	-0.70438900	1.23106300
H	1.25343600	-1.51296500	-0.00001600
H	3.41503600	-1.86244400	-0.00000900
H	4.34613900	-0.63572900	-0.88856800
H	4.34609600	-0.63575000	0.88862400
H	2.36048500	1.94369900	0.88899900
H	2.36043900	1.94372000	-0.88895500
H	3.87511700	1.67164100	-0.00002200
C	0.17445800	0.31119600	-0.00000200
H	0.17733800	1.39157800	0.00001100

Cartesian coordinates of the optimized syn-TS geometry of **2** regarding rotation around **amide** bond:

SMD($CDCl_3$)/B3LYP/6-311++G(d,p):

O	1.16634800	1.98917800	0.05179200
C	1.17539500	0.76144200	0.02269900
N	2.46741100	0.07893300	-0.01067200
C	2.68280900	-0.67551700	-1.24649900
C	2.71448400	-0.73415100	1.18113500
C	-1.25347900	0.49959400	0.02711000
N	-2.43651900	-0.12765300	0.04197700
C	-3.68710900	0.61652100	-0.06200300
C	-2.51824800	-1.58301000	0.02425000
H	2.47776000	-0.03952600	-2.11062900
H	2.06062200	-1.58329600	-1.32541900
H	3.73177300	-0.98189800	-1.29576700
H	2.53446000	-0.14037700	2.08014100
H	2.09284500	-1.64423200	1.23334300

H	3.76372000	-1.04344700	1.18694300
H	-1.31355700	1.58315500	0.02847600
H	-3.48070800	1.68598100	-0.03245000
H	-4.19915000	0.38406000	-1.00206300
H	-4.35184500	0.36194200	0.76859000
H	-1.88880100	-2.01184000	0.80818600
H	-2.19808400	-1.98681400	-0.94310500
H	-3.54954200	-1.88470800	0.20417800
C	-0.00481100	-0.07229300	0.01559200
H	0.11923400	-1.14689700	-0.00761200

SMD($CDCl_3$)/M062X/6-311++G(d,p):

O	1.15412100	1.98431000	0.02824300
C	1.17567000	0.76595200	0.01188800
N	2.46287900	0.09303300	-0.00841100
C	2.65016600	-0.69454400	-1.22378300
C	2.67512300	-0.71802900	1.18720100
C	-1.24050800	0.50423600	0.01277300
N	-2.41696600	-0.12391700	0.02042500
C	-3.67467500	0.60306500	-0.03482400
C	-2.46973100	-1.57490600	0.01884300
H	2.43950800	-0.07840500	-2.09990800
H	2.01338100	-1.59192100	-1.26154700
H	3.69335000	-1.01524200	-1.27568000
H	2.48467700	-0.11878300	2.07948600
H	2.03767000	-1.61511800	1.22129400
H	3.71854400	-1.04127700	1.21018100
H	-1.29508000	1.58905200	0.01021400
H	-3.47544800	1.67375000	-0.02855300
H	-4.22173600	0.34994100	-0.94725600
H	-4.29581700	0.35220900	0.82876900
H	-1.92728800	-1.97786800	0.87878500
H	-2.02548900	-1.97855500	-0.89660100
H	-3.50900000	-1.89378200	0.07757200
C	-0.00148000	-0.07885900	0.00995900
H	0.12098200	-1.15379100	0.00086100

SMD(Ace)/B3LYP/6-311++G(d,p):

O	1.17668000	1.99783500	0.05187000
C	1.17619600	0.76800200	0.02288300
N	2.46681700	0.07803700	-0.00905100
C	2.67536100	-0.68185600	-1.24497800
C	2.70355200	-0.74325100	1.18165300
C	-1.25658900	0.50749900	0.02251500
N	-2.43252000	-0.12683000	0.03377700
C	-3.69031300	0.60873400	-0.05585300
C	-2.50569600	-1.58395900	0.02365000

H	2.47742200	-0.04573100	-2.11098300
H	2.04262400	-1.58159900	-1.32018200
H	3.72069900	-1.00084700	-1.29175400
H	2.52737000	-0.15172800	2.08325300
H	2.07182400	-1.64571000	1.22669100
H	3.74928800	-1.06426000	1.18717600
H	-1.32562500	1.59055500	0.02171700
H	-3.49027000	1.67948900	-0.03661500
H	-4.21234100	0.36294500	-0.98643900
H	-4.33966700	0.35199800	0.78595000
H	-1.88605400	-2.00436400	0.81989900
H	-2.16634200	-1.98870900	-0.93625500
H	-3.53833100	-1.88908500	0.18722400
C	-0.00323800	-0.06148200	0.01483100
H	0.12023800	-1.13620600	-0.00593500

SMD(Ace)/M062X/6-311++G(d,p):

O	1.16172200	1.99112200	0.01583500
C	1.17582600	0.77078700	0.00679700
N	2.46247200	0.09259100	-0.00351600
C	2.65059800	-0.70429600	-1.21477300
C	2.66165800	-0.72001900	1.19545800
C	-1.24352100	0.51033600	0.00538000
N	-2.41356800	-0.12326500	0.00809700
C	-3.67716400	0.59715400	-0.01647900
C	-2.46059600	-1.57566800	0.00921200
H	2.44987800	-0.09209000	-2.09631300
H	2.00591800	-1.59544000	-1.24868700
H	3.69106700	-1.03519900	-1.25765900
H	2.46974000	-0.11930900	2.08680600
H	2.01690900	-1.61131200	1.22396000
H	3.70228300	-1.05194400	1.22411000
H	-1.30563300	1.59486000	0.00379000
H	-3.48322000	1.66869600	-0.01542100
H	-4.24258900	0.33678400	-0.91502100
H	-4.27439400	0.34012800	0.86191500
H	-1.94050300	-1.97464400	0.88484400
H	-1.98810500	-1.97721400	-0.89238000
H	-3.50017300	-1.89668500	0.03799400
C	-0.00001700	-0.07078800	0.00492400
H	0.12200600	-1.14592200	0.00020500

Cartesian coordinates of the optimized anti-TS geometry of **2** regarding rotation around **enamine** bond:

SMD($CDCl_3$)/B3LYP/6-311++G(d,p):

C	1.16696800	-0.48053700	0.06442200
O	1.16068800	-1.69521000	0.30254500
N	2.33216000	0.22624000	-0.02652200
C	2.42857800	1.67330900	-0.21448300
H	3.33967000	2.02427800	0.27427200
H	2.48429300	1.94305400	-1.27551300
H	1.59238900	2.19565500	0.24525700
C	3.60669800	-0.48094800	0.03835700
H	4.13474500	-0.24680100	0.96937600
H	3.43007700	-1.55227800	-0.00797300
H	4.23752300	-0.17956900	-0.80364200
C	-1.28613500	-0.30041500	0.09197300
H	-1.33154600	-1.32192300	0.48205000
N	-2.52698100	0.37206500	-0.13059700
C	-3.33874300	-0.36257900	-1.11067500
H	-2.79738300	-0.44025600	-2.05551200
H	-4.26631800	0.18696900	-1.28862400
H	-3.59758500	-1.38015700	-0.77109800
C	-3.25735200	0.53485600	1.13395700
H	-2.65721900	1.11709600	1.83587700
H	-3.51028900	-0.42993200	1.60627000
H	-4.18629900	1.07725900	0.94175500
C	-0.10454100	0.26709600	-0.15749700
H	-0.08665400	1.27546000	-0.54934600

SMD($CDCl_3$)/M062X/6-311++G(d,p):

C	1.16436800	-0.48446400	0.05400500
O	1.14723700	-1.68022900	0.34089300
N	2.32081700	0.21783000	-0.04784300
C	2.39037200	1.66014700	-0.25167400
H	3.34413800	2.01212300	0.14225000
H	2.33649900	1.92429100	-1.31239100
H	1.60071600	2.17679000	0.29113400
C	3.59313300	-0.47473100	0.08057400
H	4.05006200	-0.27412100	1.05474200
H	3.43512100	-1.54493800	-0.02306500
H	4.27130100	-0.12959500	-0.70318100
C	-1.27268000	-0.27541200	0.09633400
H	-1.31432800	-1.25615500	0.58155200
N	-2.51374500	0.37499200	-0.16474000
C	-3.33841500	-0.47268700	-1.02852100
H	-2.82317700	-0.64724100	-1.97403600
H	-4.28037800	0.03972500	-1.23339000
H	-3.56229600	-1.44578300	-0.56286400
C	-3.20930800	0.63276800	1.09788400
H	-2.59933100	1.27999800	1.72954300
H	-3.42689200	-0.29627300	1.64885000

H	-4.15259900	1.13988800	0.88602000
C	-0.09922500	0.26167600	-0.22443500
H	-0.07571000	1.22959400	-0.70923200

SMD(Ace)/B3LYP/6-311++G(d,p):

C	1.16874000	-0.48235700	-0.06520000
O	1.16460700	-1.69399800	-0.32535300
N	2.33044100	0.22474800	0.03713000
C	2.42622300	1.67296300	0.22381000
H	3.31641100	2.02906500	-0.29878100
H	1.56951500	2.19061300	-0.20123800
H	2.52116300	1.93754400	1.28298400
C	3.60771700	-0.47646000	-0.04338800
H	4.26094500	-0.13441400	0.76451500
H	3.44364900	-1.54673900	0.05178700
H	4.10446500	-0.27539600	-0.99928100
C	-1.28637700	-0.29578100	-0.10278400
H	-1.33601900	-1.29957100	-0.53573000
N	-2.52717600	0.37005200	0.14064400
C	-3.25690200	0.57220000	-1.11973600
H	-2.65762300	1.17934200	-1.80124900
H	-4.18844500	1.10355500	-0.91022700
H	-3.50446800	-0.37840700	-1.62197200
C	-3.34098500	-0.39841000	1.09433500
H	-2.80283300	-0.50533400	2.03836300
H	-3.59465000	-1.40433300	0.71900100
H	-4.27103100	0.14258900	1.28493600
C	-0.10449300	0.25775000	0.17607100
H	-0.08433200	1.25044600	0.60665200

SMD(Ace)/M062X/6-311++G(d,p):

C	1.16683100	-0.48747600	-0.05213200
O	1.15182300	-1.68347400	-0.34372600
N	2.31931200	0.21770200	0.05103500
C	2.38353000	1.66206100	0.25121200
H	3.33502400	2.01566300	-0.14625300
H	1.58929400	2.17318500	-0.28998800
H	2.33107200	1.92634900	1.31167600
C	3.59485700	-0.46862200	-0.08280900
H	4.28282600	-0.09562200	0.67882200
H	3.44900400	-1.53715600	0.05256200
H	4.03335800	-0.28921900	-1.06960100
C	-1.27245000	-0.27433100	-0.09995700
H	-1.31898500	-1.24710500	-0.60033800
N	-2.51296800	0.37556100	0.16635500
C	-3.21003700	0.63915100	-1.09545600
H	-2.60180400	1.29162000	-1.72372800

H	-4.15514500	1.14125100	-0.87964500
H	-3.42400000	-0.28846700	-1.64968900
C	-3.33829100	-0.47875600	1.02464700
H	-2.82522800	-0.65668600	1.97098200
H	-3.55735500	-1.44918600	0.55176300
H	-4.28242500	0.03034000	1.22790400
C	-0.09829400	0.25509600	0.23200100
H	-0.07150200	1.21725000	0.72857600

Cartesian coordinates of the optimized syn-TS geometry of **2** regarding rotation around **enamine** bond:

SMD($CDCl_3$)/B3LYP/6-311++G(d,p):

C	-1.14910200	-0.55804800	-0.06962100
O	-1.33679000	-1.75610700	-0.31388800
N	-2.19179100	0.30899100	0.09050500
C	-2.07745300	1.76252800	0.18054200
H	-2.86596800	2.21580200	-0.42611500
H	-2.19612900	2.11196400	1.21261400
H	-1.12599700	2.11415600	-0.21001100
C	-3.55601300	-0.20783600	0.07643400
H	-4.02787100	-0.05564200	-0.90143700
H	-3.54761900	-1.27226200	0.29800100
H	-4.14613600	0.31693300	0.83250400
C	1.29213500	-0.69911000	-0.33629900
H	1.14039700	-1.65190200	-0.83879700
N	2.66125000	-0.28411600	-0.19355600
C	3.10173300	-0.22429100	1.20276800
H	2.88576300	-1.17267600	1.69938000
H	2.62092100	0.58492800	1.77772900
H	4.18318800	-0.06440700	1.22777400
C	2.97567400	0.94398400	-0.92740000
H	2.66646600	0.84159700	-1.96990700
H	4.05719200	1.10405000	-0.90701500
H	2.48862700	1.84050400	-0.50784900
C	0.23083800	-0.01289000	0.09748800
H	0.36839900	0.93567300	0.60211700

SMD($CDCl_3$)/M062X/6-311++G(d,p):

C	-1.14533000	-0.56828200	-0.05795600
O	-1.33673500	-1.75863500	-0.29666800
N	-2.16809400	0.31136300	0.08884400
C	-2.00282400	1.75617200	0.18387500
H	-2.87733600	2.22998000	-0.26425000
H	-1.92477700	2.08856600	1.22390700
H	-1.12894900	2.09125100	-0.37160000

C	-3.53730800	-0.17770500	0.06524100
H	-3.99212600	-0.02720600	-0.91921000
H	-3.54900400	-1.23894700	0.30060600
H	-4.12202800	0.36615600	0.80997600
C	1.28053600	-0.71365500	-0.34747900
H	1.11612700	-1.65908900	-0.85966000
N	2.64758600	-0.29777500	-0.21685700
C	3.07012300	-0.23166400	1.18108000
H	2.87221000	-1.18591600	1.67167300
H	2.55604700	0.56412500	1.74207500
H	4.14497000	-0.04060500	1.21640000
C	2.91214900	0.96252400	-0.90765000
H	2.59861200	0.88495600	-1.94991900
H	3.98618100	1.15984600	-0.88382700
H	2.39179500	1.81690300	-0.44733300
C	0.23646500	-0.02608200	0.11164500
H	0.38284400	0.91610200	0.62805500

SMD(Ace)/B3LYP/6-311++G(d,p):

C	-1.15210300	-0.56211500	-0.06779700
O	-1.34065700	-1.77011100	-0.26820900
N	-2.18871300	0.31431300	0.05981800
C	-2.05306800	1.76814000	0.15639200
H	-2.91708400	2.22721100	-0.32810400
H	-2.02606200	2.10368100	1.19920000
H	-1.16332600	2.12179800	-0.35969400
C	-3.55825900	-0.18903900	0.07665800
H	-4.06231500	0.01214200	-0.87554800
H	-3.55149900	-1.26181800	0.25241900
H	-4.11613900	0.30756800	0.87528400
C	1.29380500	-0.73337100	-0.28305100
H	1.14821700	-1.72752400	-0.70013300
N	2.66195800	-0.30659300	-0.16015200
C	3.07917900	-0.11502200	1.23325100
H	2.86136800	-1.01598600	1.81132100
H	2.58205100	0.73954300	1.72073600
H	4.15876500	0.05655800	1.25987600
C	2.98032000	0.85707000	-0.99444800
H	2.69078000	0.66018600	-2.02920800
H	4.05994400	1.02837400	-0.96764100
H	2.47688200	1.77957600	-0.66190900
C	0.22805300	-0.00978100	0.07257100
H	0.36340100	0.98024600	0.48995300

SMD(Ace)/M062X/6-311++G(d,p):

C	-1.14643100	-0.57033200	-0.05757100
O	-1.33840700	-1.76456200	-0.28513400

N	-2.16543800	0.31213600	0.08051100
C	-1.99368900	1.75742700	0.18023100
H	-2.88002200	2.23354500	-0.24048800
H	-1.88651900	2.08094900	1.22010700
H	-1.13381900	2.09375800	-0.39597700
C	-3.53772100	-0.16939700	0.06467400
H	-4.00078600	-0.00151700	-0.91302700
H	-3.55343700	-1.23335900	0.28692600
H	-4.11198000	0.36920800	0.82118200
C	1.28153700	-0.72463800	-0.33601000
H	1.11961500	-1.68059400	-0.82930100
N	2.64854000	-0.30501800	-0.21080600
C	3.06386800	-0.20592400	1.18888800
H	2.87156400	-1.15165700	1.69852300
H	2.53899100	0.59735000	1.72770800
H	4.13672400	-0.00392600	1.22479600
C	2.90922900	0.94600000	-0.92345700
H	2.60253200	0.84719700	-1.96618600
H	3.98177800	1.15079900	-0.89499600
H	2.37884100	1.80228400	-0.48003700
C	0.23630600	-0.02708500	0.10544500
H	0.38346200	0.92632100	0.60070500

Activation parameters for amide and enamine rotations of studied compounds:

Table S9. Activation parameters for amide rotation of compound **1**

Method	Energy barriers	ΔH$^{\neq}$(298K)	ΔS$^{\neq}$(298K)	ΔG$^{\neq}$(298K)	ΔG$^{\neq}$(298K)^{eff}
A	anti-TS – GS	15.6	-6.0	17.3	17.2
	syn-TS – GS	15.6	-7.9	17.9	
B	anti-TS – GS	15.2	-7.5	17.4	
	syn-TS – GS	14.7	-6.4	16.6	16.5
C	anti-TS – GS	15.1	-5.6	16.8	16.6
	syn-TS – GS	15.1	-7.1	17.2	
D	anti-TS – GS	14.8	-9.4	17.6	
	syn-TS – GS	14.3	-9.1	17.0	16.8
	2D EXSY in $CDCl_3$	15.3 ± 1.1	-3.9 ± 3.7	16.4 ± 0.1	
	2D EXSY in TCE-d2	14.9 ± 0.5	-5.0 ± 1.9	16.4 ± 0.1	
	CLSA in TCE-d2	14.4 ± 0.4	-6.7 ± 1.4	16.4 ± 0.1	
	All data in TCE-d2	14.4 ± 0.3	-6.7 ± 1.0	16.4 ± 0.1	

ZPE, thermal and entropy corrections are calculated at the corresponding level of theory for 298K. A: SMD($CDCl_3$)/B3LYP/6-311++G(d,p); B: SMD($CDCl_3$)/M062X/6-311++G(d,p); C: SMD(TCE)/B3LYP/6-311++G(d,p) and D: SMD(TCE)/M062X/6-311++G(d,p).

Table S10. Activation parameters for enamine rotation of compound **1**

Method	Energy barriers	ΔH$^{\neq}$(298K)	ΔS$^{\neq}$(298K)	ΔG$^{\neq}$(298K)	ΔG$^{\neq}$(298K)^{eff}
A	anti-TS – GS	19.6	-3.8	20.7	20.7
	syn-TS – GS	21.4	-5.4	23.0	
B	anti-TS – GS	19.0	-4.6	20.4	20.3
	syn-TS – GS	20.0	-4.9	21.5	
C	anti-TS – GS	19.5	-5.1	21.0	21.0
	syn-TS – GS	21.6	-5.3	23.1	
D	anti-TS – GS	19.0	-8.1	21.4	21.3
	syn-TS – GS	20.2	-7.4	22.4	
	2D EXSY in $CDCl_3$	19.3 ± 1.0	2.2 ± 2.4	18.6 ± 0.1	
	2D EXSY in TCE-d2	21.6 ± 0.8	9.1 ± 2.5	18.9 ± 0.1	
	CLSA in TCE-d2	20.9 ± 0.8	6.2 ± 2.0	19.1 ± 0.1	
	All data in TCE-d2	19.8 ± 0.5	3.2 ± 1.2	18.8 ± 0.1	

ZPE, thermal and entropy corrections are calculated at the corresponding level of theory for 298K. A: SMD($CDCl_3$)/B3LYP/6-311++G(d,p); B: SMD($CDCl_3$)/M062X/6-311++G(d,p); C: SMD(TCE)/B3LYP/6-311++G(d,p) and D: SMD(TCE)/M062X/6-311++G(d,p).

Table S11. Activation parameters for amide rotation of compound **2**

Method	Energy barriers	ΔH≠(298K)	ΔS≠(298K)	ΔG≠(298K)	ΔG≠(298K)eff
A	anti-TS – GS<br>syn-TS – GS	10.6<br>13.0	-6.6<br>-7.5	12.5<br>15.2	12.5
B	anti-TS – GS<br>syn-TS – GS	10.5<br>12.6	-7.8<br>-6.4	12.8<br>14.5	12.8
C	anti-TS – GS<br>syn-TS – GS	10.4<br>12.3	-7.0<br>-7.6	12.5<br>14.5	12.5
D	anti-TS – GS<br>syn-TS – GS	10.5<br>11.9	-12.9<br>-9.9	14.3<br>14.8	14.1
	Experiment in Acetone-d6			12.4 (Tc=253K)	

ZPE, thermal and entropy corrections are calculated at the corresponding level of theory for 298K. A: SMD($CDCl_3$)/B3LYP/6-311++G(d,p); B: SMD($CDCl_3$)/M062X/6-311++G(d,p); C: SMD(Ace)/B3LYP/6-311++G(d,p) and D: SMD(Ace)/M062X/6-311++G(d,p).

Table S12. Activation parameters for enamine rotation of compound **2**

Method	Energy barriers	ΔH≠(298K)	ΔS≠(298K)	ΔG≠(298K)	ΔG≠(298K)eff
A	anti-TS – GS<br>syn-TS – GS	11.6<br>15.6	-2.7<br>-4.2	12.4<br>16.9	12.4
B	anti-TS – GS<br>syn-TS – GS	10.9<br>14.4	-3.8<br>-4.7	12.1<br>15.8	12.1
C	anti-TS – GS<br>syn-TS – GS	11.9<br>15.8	0.5<br>-4.2	11.8<br>17.0	11.8
D	anti-TS – GS<br>syn-TS – GS	11.3<br>14.5	-8.6<br>-9.5	13.9<br>17.4	13.8
	Experiment in Acetone-d6			11.7 (Tc=253K)	

ZPE, thermal and entropy corrections are calculated at the corresponding level of theory for 298K. A: SMD($CDCl_3$)/B3LYP/6-311++G(d,p); B: SMD($CDCl_3$)/M062X/6-311++G(d,p); C: SMD(Ace)/B3LYP/6-311++G(d,p) and D: SMD(Ace)/M062X/6-311++G(d,p).
